# YouTube’s recommendation algorithm is left-leaning in the United States

**DOI:** 10.1093/pnasnexus/pgad264

**Published:** 2023-08-14

**Authors:** Hazem Ibrahim, Nouar AlDahoul, Sangjin Lee, Talal Rahwan, Yasir Zaki

**Affiliations:** Department of Computer Science, New York University Abu Dhabi, Abu Dhabi 129188, UAE; Department of Computer Science, New York University Abu Dhabi, Abu Dhabi 129188, UAE; Department of Computer Science, New York University Abu Dhabi, Abu Dhabi 129188, UAE; Department of Computer Science, New York University Abu Dhabi, Abu Dhabi 129188, UAE; Department of Computer Science, New York University Abu Dhabi, Abu Dhabi 129188, UAE

**Keywords:** recommendation systems, political radicalization, algorithmic bias

## Abstract

With over two billion monthly active users, YouTube currently shapes the landscape of online political video consumption, with 25% of adults in the United States regularly consuming political content via the platform. Considering that nearly three-quarters of the videos watched on YouTube are delivered via its recommendation algorithm, the propensity of this algorithm to create echo chambers and deliver extremist content has been an active area of research. However, it is unclear whether the algorithm may exhibit political leanings toward either the Left or Right. To fill this gap, we constructed archetypal users across six personas in the US political context, ranging from Far Left to Far Right. Utilizing these users, we performed a controlled experiment in which they consumed over eight months worth of videos and were recommended over 120,000 unique videos. We find that while the algorithm pulls users away from political extremes, this pull is asymmetric, with users being pulled away from Far Right content stronger than from Far Left. Furthermore, we show that the recommendations made by the algorithm skew left even when the user does not have a watch history. Our results raise questions on whether the recommendation algorithms of social media platforms in general, and YouTube, in particular, should exhibit political biases, and the wide-reaching societal and political implications that such biases could entail.

**Significance Statement**

We analyze YouTube’s recommendation algorithm by constructing archetypal users with varying political personas, and examining videos recommended during four stages of each user’s life cycle: (i) after their account is created; (ii) as they build a political persona through watching videos of a particular political leaning; (iii) as they try to escape their political persona by watching videos of a different leaning; (iv) as they watch videos suggested by the recommendation algorithm. We find that while the algorithm pulls users away from political extremes, this pull is asymmetric, with users being pulled away from Far-Right content faster than from Far-Left. These findings raise questions on whether recommendation algorithms should exhibit political biases, and the societal implications that such biases could entail.

The proliferation of online media consumption via YouTube has become a significant cultural phenomenon due to its position as a source of information and entertainment with more than 2 billion monthly active users worldwide (1). YouTube’s content continues to grow at a rapid scale, with over 500 h worth of content being uploaded each minute, covering a wide array of topics, including news and politics (2). According to a recent survey conducted by the Pew Research Center, 25% of adults in the United States regularly get their news from YouTube, making it the second most popular online news source (3). The study also found that 60% of adults who use YouTube claim that they use the platform to keep up with current events regularly. Importantly, the means by which individuals consume content on YouTube differ, as users can either search for a particular video or watch videos recommended to them. According to YouTube’s CPO, 70% of videos watched on YouTube come via its recommendation algorithm (4). As such, this algorithm has been a subject of much discussion in recent years. While it is designed to personalize recommendations based on a user’s viewing history, it has also been criticized for contributing to filter bubbles and echo chambers (5).

The existence of echo chambers online, and the means by which one may enter or escape them, have been a prevalent area of research over the last several decades. Defined as a situation or space in which preexisting beliefs are repeated and reinforced, echo chambers, particularly in the context of social media, have been analyzed through the lenses of homophily (6), selective exposure (7), and confirmation bias (8). In the context of politics, echo chambers have been studied across a multitude of platforms, including Twitter (9–14), Facebook (8, 14–17), Reddit (14, 18, 19), and more recently YouTube (20). Furthermore, studies have examined the impact of recommendation algorithms, and their subsequent creation of echo chambers, on reinforcing political opinions (21).

While prior literature on echo chambers is relatively extensive, fewer studies have examined the interplay between YouTube’s recommendation algorithm and political radicalization. Nonetheless, there have been several influential studies which aimed to classify YouTube videos into distinct categories (22–25), as well as understand potential radicalization pathways influenced by the recommendation algorithm (21, 26–28). Most relevant to our line of inquiry, Ledwich et al. classified the videos of over 800 channels into different political categories, and through an analysis of video recommendations, found little evidence supporting the algorithm recommending radicalized content (29). Supporting these findings, Hosseinmardi et al. also found no evidence that engagement with Far-Right content is caused by YouTube recommendations through a longitudinal study which involved content consumption both on and external to YouTube (30). The results of these studies, while interesting, cannot be interpreted causally due to their observational nature.

Despite the above research, the literature still lacks a controlled experiment that examines the rate at which the YouTube recommendation algorithm adapts to a user’s political preference through the process of “personalization.” Furthermore, no prior study has examined the feasibility and speed by which one may escape a political persona on YouTube. To fill this gap, we start off by examining the distribution of videos recommended to a new YouTube user based in the United States with regard to their category as labeled by YouTube, and their political orientation for news and political videos specifically. Moreover, we explore the rate at which the recommendation algorithm adapts to a user’s political preference by having them watch a sequence of videos that fall under a specific political class, and collecting the top recommendations after each video is watched. Furthermore, we investigate how quickly a user may escape their political persona by having them watch yet another sequence of videos that fall under a different political class, and collecting the recommendations made by the algorithm after each video watched. Our results reveal that, in the US political context, YouTube tends to recommend left-leaning videos by default, while also enabling a user to fall into a left-leaning political persona more quickly. Furthermore, we observe that it is more difficult to escape left-leaning political personas than their right-leaning counterparts. We also find that users are pulled towards the ideological center and away from the extremes. However, this pull is asymmetric, with users being pulled away from Far Right content more aggressively than Far Left. Finally, we show that this asymmetric pull is not motivated by a concentration of videos containing fake news claims or misinformation on the Right end of the political spectrum but rather that such videos are uniformly distributed across all political classes. These findings reveal, for the first time, that Youtube’s recommendation algorithm is left-leaning in the context of US politics.

## Methods

### Experimental design

We employ 360 bots to simulate YouTube users by performing both predefined and real-time sequences of video watches. In order to isolate the “personalization” process undertaken by the YouTube recommendation algorithm, we create a new Google and YouTube account for each bot used in the experiment. Note that YouTube may still recommend videos to a user despite them not having a YouTube account, implying that such recommendations are independent of the user’s watch history. However, since we are interested in personalized recommendations, i.e. those dependent on the watch history, we create new accounts for each bot. For the remainder of this article, we will use the terms user and bot interchangeably. Our experiment consists of three stages, each of which requires the user to watch one or more videos while recording the recommended videos offered by YouTube after each video is watched. Utilizing the classification mechanism employed by Hosseinmardi et al. (30), we split our users into an initial set of six groups, namely: Far Left, Left, Center, Anti-Woke, Right, and Far Right. Each group includes a set of 60 users, with 360 users in total used in the experiment.

To avoid being detected by Google as a bot, accounts were made using the most common American first names and surnames as listed in (31). Furthermore, all accounts were created as males between the ages of 30 and 40, and each account was linked to a distinct phone number. After a new Google account is made, we collect the top 20 recommended videos on the YouTube homepage in order to obtain a baseline of the distribution of content recommended by the algorithm without any watch history. It should be noted that YouTube occasionally asks a new user to watch one video before recommendations are made by the algorithm. In our case, this happened with 88 users, leaving us with 272 users for which the baseline recommendations were collected.

**Fig. 1.**
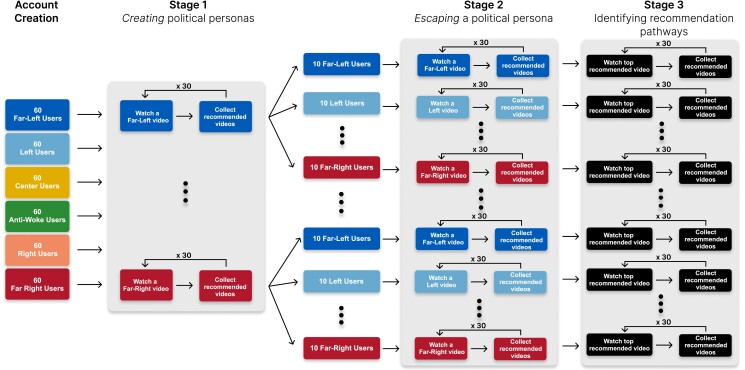
Experiment setup. An illustration of the different stages undertaken during our experiment. In the account creation stage, users are designated one of six political classes which denote the class of videos they will watch in Stage 1. In Stage 1, each user watches 30 videos of a given political class. After each video is watched, the videos recommended to them are collected. After Stage 1, each user is designated a new political class, which will denote the class of videos which they will watch in Stage 2. Similarly, in Stage 2, users watch 30 videos of a given political class, and the videos recommended to them are collected. Finally, in Stage 3, each user will watch the top recommended video to them and subsequently, the set of recommended videos after each video is watched is collected.

The experimental setup is illustrated in Fig. 1. In particular, the first stage involves having each user watch 30 videos whose political leaning matches that of the user’s designated group. After each video is watched, we collect the top 20 recommended videos from the YouTube homepage. This is done to estimate how quickly the recommendation algorithm adapts to the user’s preferences. After this stage, each group of 60 users is further split into 6 subgroups, each of which corresponds to one of the 6 aforementioned political classes. These subgroups specify the set of videos to be watched in the second stage of the experiment. Here, the users watch 30 videos from their newly designated subgroup, and the top 20 recommendations made by YouTube are collected after each video is watched. This allows us to determine the speed by which YouTube adjusts its recommendations to the new content that the user consumes. In the third and final stage of the experiment, each user watches the top recommended video on their YouTube homepage while collecting the recommendations made by the algorithm. After the recommendations are collected, the user restarts the YouTube app and repeats this process 30 times. This stage of the experiment aims to determine the recommendation pathways through which the algorithm may lead a particular user.

This experiment was conducted on 36 identical mobile phones, namely the Xiaomi Redmi Go, running Android 8.1. These phones were programmed to execute ADB commands (32) which allowed for the control of button clicks, opening YouTube videos, and scraping the recommendations made to a user. To control for the effect of having multiple users share an IP address, we uniformly distributed the profiles to be used in the experiment across all 36 phones, with each phone completing 10 users in total, each of which fell under a different group/subgroup pair. Furthermore, after completing the experiment with any given user, the Google account associated with that user was wiped from the phone, and the phone was factory-reset. The experiment was conducted at the authors’ institution in the United Arab Emirates. However, each phone was connected via VPN to the United States, since our experimental focus is primarily with regard to the US political context. On average, each experiment run took roughly 28 to 30 h to complete, and the entire experiment took 3 weeks to complete, between 13 December 2022 and 3 January 2023. In total, through utilizing 360 users to watch different sets of videos, over 8 months worth of YouTube content was watched and 120,880 distinct videos were recommended by the platform.

### Fake news detection

To determine whether a given video contains fake news, we adopt a similar methodology to that employed by Vosoughi et al. (33) in their study on the spread of Fake News on Twitter. First, we utilize the political fact-checking websites snopes.com, as well as politifact.com, to collect all news headlines labeled as “False,” “Mostly False,” “Unproven,” or “Unfounded” in the case of Snopes, and “False,” “Mostly False,” or “Pants on fire” in the case of Politifact, amounting to a total of 17,689 unique false headlines. Next, we collect all available titles and transcripts of videos in the dataset described in the following section, amounting to over 11 million titles and 9 million transcripts. The difference between the number of titles and transcripts is due to the subset of videos that do not include a transcript. We then utilize MPNet, a state-of-the-art pretrained language model developed by Microsoft (34) to convert the headline, the video title and the video transcript to vectors that capture their semantic context. We then used cosine similarity to measure the distance between the vectors, allowing us to quantify what proportion of videos in the dataset mention content deemed to be “Fake News.”

### Data

In this experiment, the videos watched by the users fall under two categories, namely, those with a known political classification (pre-labeled) according to previous literature, and those that were not previously classified (unlabeled). The pre-labeled videos are taken from two previous studies on the YouTube recommendation algorithm (29, 35). Specifically, Ledwich and Zaitsev (29) utilized a snowball sampling methodology, where they started by collecting all videos made by YouTube accounts which had over 10,000 subscribers, or over 10,000 views per month, and where 30% or more of their videos were related to politics. Next, they recursively added emerging channels which fit their selection criteria based on recommendations made by the algorithm when watching videos of a known channel. Next, they categorize the videos collected into different categories, which included “Conspiracy,” “Anti-SJW,” “Late Night Talk shows,” “Socialist,” among others. On the other hand, Riberio’s work (35) primarily focused on three specific communities on YouTube, namely the “Intellectual Dark Web,” “Alt-lite,” and “Alt-right.” They adopt a similar snowball sampling methodology where they begin with known channels which fall under a particular community and build their dataset by sampling recommended channels stemming from a particular seed. The resulting videos from these two datasets were then grouped into the six aforementioned political classes (Far Left, Left, etc.) in accordance with (30), amounting to 11.5 million videos in total. Since each of the two aforementioned studies do not share the same categories for classifying a particular YouTube video, Hosseinmardi et al. group the sets of videos into the six political classifications used in our study; the exact mapping can be seen in Appendix Table S1 of (30). It should be noted that the vast majority of videos collected in these datasets are primarily concerned with the US political zeitgeist, and as such, our experimental focus is also primarily concerned with any asymmetric political recommendations made by YouTube’s algorithm in the context of US politics.

Out of 11.5 million videos, we sample a subset of 30 videos for each user in the first stage of the experiment, and another 30 in the second stage, weighted by the number of views that each video has received as of 15 November 2022. Moreover, for the initial two stages of the experiment, we split the 30 videos watched into three types: (i) short videos whose length is under 2 min, (ii) medium videos whose length is between 2 and 10 min, and (iii) long videos whose length is greater than 10 min. In the first and second stages of the experiment, each user watched 10 videos of each type of length in order to control for total watch time.

## Results

### Baseline recommendations

**Fig. 2.**
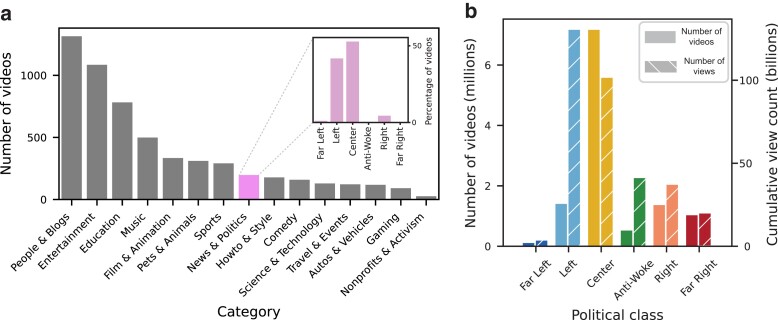
Baseline recommendations and dataset description. (a) A summary of the categories and political classes of the videos recommended to new users. The main plot illustrates the number of videos from each category for the videos recommended to new users. The inset illustrates the distribution of political classifications for videos falling under the “News & Politics” category. (b) The number of videos (left *y*-axis) and cumulative number of views (right *y*-axis) across political classes for all videos in the dataset.

We start with an exploratory analysis of the types of videos recommended to a new account, i.e. one that does not have a watch history. To this end, we examine the distribution of categories of the recommendations. Moreover, for those whose category happens to be “News & Politics,” we examine the distribution of their political classes. The results of this analysis are summarized in Fig. 2a. As can be seen, the majority of videos recommended to users fell under the categories of “People & Blogs,” “Entertainement,” “Education,” and “Music,” indicating that users are not typically exposed to videos relating to “News & Politics” without a prior watch history. Indeed, videos relating to “News & Politics” amounted to 3% of all videos recommended to new accounts. However, for those videos, the distribution of political classes, which can be seen in the inset of Fig. 2a, indicates that a vast majority of such videos fall under both the Center (51%) and Left (42%) political classes, with a few videos falling under the Right class (6%). In contrast, very few, if any videos fell under the Far Left (1%), Anti-Woke (0%), or Far Right (0%) classes. Fig. 2b illustrates the distribution of the number of videos (left *y*-axis), as well as the view count of videos (right *y*-axis) for each political class within our dataset. As can be seen, while the Center class constituted the majority of videos, those within the Left class garnered the largest number of views. This is in line with previous research showing that “Right-wing YouTube” has fewer videos on the platform, and fewer views on average compared to their Left-wing counterparts (28). Performing a chi-squared test between the distribution of labeled videos recommended during the baseline stage against that of our entire dataset indicates a statistically significant (P<0.0001) difference, thus indicating a disproportionate skew towards left-leaning videos when accounting for the distribution of videos in the pre-labeled dataset. Detailed counts of the number of videos falling under each category and political classification can be found in Supplementary Table 1 and details of the chi-squared test can be seen in Supplementary Table 2.

### Building a political persona

**Fig. 3.**
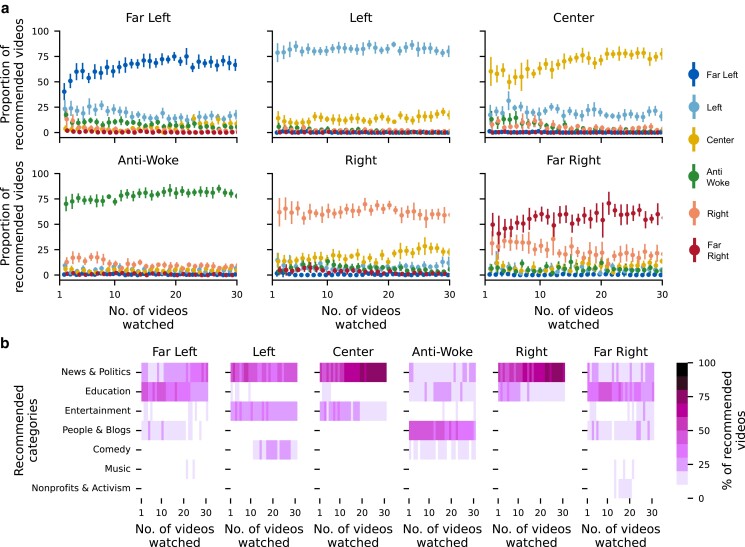
Distribution of recommendations made after watching videos of a particular political class. (a) Proportion of recommended videos falling under each political class after each video is watched, with error bars representing 95% confidence intervals. The proportions of videos falling under each political class after each video watched sum up to 100%. (b) Category distribution of recommended videos after each video is watched, where the proportion of videos falling under each category sum up to 100%. The title of each plot indicates the political classification of the videos being watched.

We continue our analysis by looking at the first stage of the experiment where each user watches 30 videos of a particular political class. After each video is watched, the top 20 recommendations on the user’s YouTube homepage are collected. In this stage, our goal is to understand the distribution of recommendations offered to the user after each video is watched, both with respect to their political classification (Far Left, Left, etc.). The results of this analysis are depicted in Fig. 3a, where the *x*-axis corresponds to the time steps in the first stage (i.e. the 30 videos being watched), while the *y*-axis corresponds to the proportion of recommendations falling under each political class after each video is watched. It should be noted that not all videos recommended to the user are related to politics. As such, in this analysis, we only consider instances in which 50% or more of the 20 recommendations are labeled in our dataset. As can be seen, for all political classes, the majority of recommendations match the video being watched in terms of their political classification. After only a single video is watched (the left-most point in each plot), the proportion of recommendations matching the watched video was highest for the Left (78%) and Anti-Woke (70%) classes and lowest for the Far Left (43%) and Far Right (50%) classes. In the cases of both extremes, namely, Far Left and Far Right, a proportion of the videos recommended fell under the classification of their less extreme counterparts (i.e. Left and Right, respectively). However, there were very few, if any, instances of recommendations in the opposite direction—almost no Far Left or Far Right videos were recommended after watching Left and Right videos, respectively. Furthermore, we account for the distribution of videos in our dataset by computing the Bhattacharyya distance (36) between the distribution of labeled recommendations against that of our dataset. The Bhattacharyya coefficient quantifies the “closeness” of two random statistical samples, which in turn allows us to quantify the distance between the distribution of videos recommended for each user against that of the pre-labeled dataset which includes over 11.5 million videos. This analysis allows us to control for the numerical asymmetry with regard to the number of videos available in each political class in the pre-labeled dataset. Thus, if the left-leaning signal seen in this analysis were to stem from the distribution of videos from the pre-labeled dataset, the “closeness” of the videos recommended when watching Left videos would be closer to that of the pre-labeled dataset than their Right-leaning counterparts. However, our results indicate that this is not the case, as users watching Far Left and Left videos are recommended a disproportionately larger proportion of videos falling under the same category when compared to those on the Right and Far Right. In other words, even when accounting for the fact that the Left political class contains more videos than the Right, we still find a statistically significant difference in the proportion of videos recommended to each political class. Supplementary Tables 4–9 specify the proportion of labeled recommendations across political classifications during this stage of the experiment, while Supplementary Table 3 provides the Bhattacharyya distance values.

Fig. 3b illustrates the distribution of video categories (Entertainment, News & Politics, etc.) for the recommendations during the first stage. Here, we are interested in understanding whether YouTube continues to recommend nonpolitical videos as a user exclusively watches videos related to politics. As can be seen, in the case of Center and Right, as the number of videos watched increased, so does the proportion of recommendations which fall under the News & Politics category. However, in the case of Left, a larger proportion of recommendations fall under the Entertainment and Comedy categories, due to talk shows being labeled as Left in the original dataset. For Anti-Woke users, the largest proportion of recommendations fall under the People & Blogs category, due to many popular podcasts such as the “Joe Rogan Podcast” falling both under the Anti-Woke political classification, as well as the People & Blogs category. Interestingly, in the case of both extremes, we see similar proportions of recommendations falling under the Education category, due to “educational” videos on the political ideologies of each extreme (e.g. Socialism, Fascism) falling under their respective political classifications.

### Escaping a political persona

Next, we explore the ease by which one can escape their political persona. More specifically, having watched 30 videos in the first stage of the experiment (which determined the user’s “original class”), we now move on to the second stage, where the user watches 30 videos of a different political classification, which we refer to as the “new class.” We examine the average number of videos watched before the proportion of recommendations falling under the new class exceeds that of the original class, and continues to exceed it for the remainder of the 30 videos. In such a case, we say that the user has “escaped” the original class, and has “entered” the new class.

**Fig. 4.**
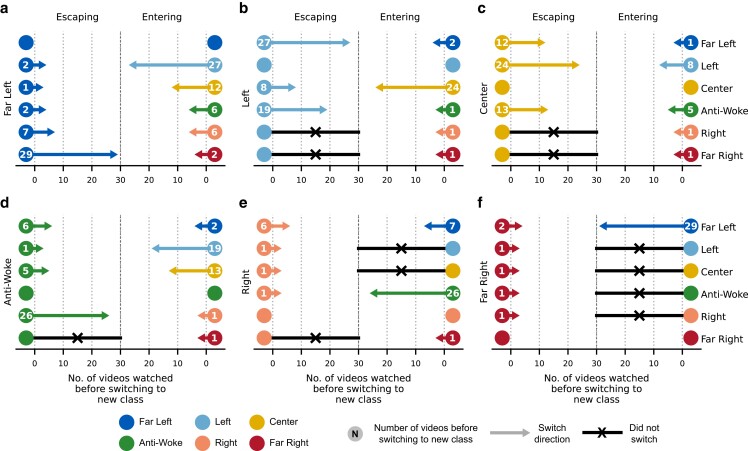
Escaping political personas. The average number of videos watched until the user reaches a point at which the proportion of recommended videos with the new class exceeds that of the original class. For instance, subplot (a) shows the results when Far Left is the original class that the user is trying to escape, as well as the results when Far Left is the new class that the user is trying to enter. A black line indicates that this point was not reached after 30 videos of the new class were watched.

The results of this analysis are summarized in Fig. 4. Within each subplot, the left-hand side (labeled “Escaping”) depicts the average number of videos required to escape a particular class, while the right-hand side (labeled “Entering”) depicts the average number of videos required to enter a class. This is done for all pairs of political classes. The arrow indicates the escape direction, while the arrow’s length corresponds to the average number of videos needed to escape in this direction, which we will call “escape speed.” The escape speed for a given pair of classes is also shown numerically within each vertex. For instance, in Fig. 4a, it takes 2 videos on average to escape Far Left and enter Left, while it takes 27 videos to escape Left and enter Far Left. A black arrow indicates that watching all 30 videos of the new class was insufficient to escape the original class. As can be seen, it is most difficult to escape the Left (Fig. 4b) and Center (Fig. 4c) political personas, as users from both classes were unable to escape in two cases (Right and Far Right) and had to watch a relatively large number of videos (18 for Left, and 16.3 for Center, on average) before escaping in the remaining three cases. Furthermore, the Left and Center personas were the easiest to enter, with other classes requiring an average of only 5.8 and 3.2 videos to enter Left and Center, respectively. In contrast, a Far Right persona was by far the easiest to escape, requiring only 1.2 videos on average to switch to a new political class. Furthermore, the Far Right class was the most difficult to enter, with four classes (Left, Center, Anti-Woke, and Right) failing to switch to Far Right, and Far Left requiring 29 videos on average to enter the Far Right class.

The results in Fig. 4 demonstrate that there exists asymmetry in escape speed between pairs of classes falling on opposite ends of the political spectrum. While a user with a Far Left political persona requires 29 videos to switch to a Far Right persona, it only took 2 videos on average to switch in the opposite direction. This asymmetry exists despite the fact that the Far Right class includes more videos as well as more cumulative views as a whole (see Fig. 2b). Similarly, for Left and Right personas, Left personas were unable to switch to Right personas after 30 videos, while it only took 1 video to switch from Right to Left. These findings highlight the asymmetry in the YouTube’s political recommendation rates across classes in the US political context, suggesting a skew towards left-leaning content. To verify that these results are not due to the general trend of the news during the time of the experiment, we repeated this experiment on the two extreme political classes (Far Left and Far Right) six months later, and again found a statistically significant difference (P<0.0001) in the strength of pull away from Far Left vs. Far Right; see Supplementary Fig. 13.

One possible explanation behind this finding is that the Right and Far Right classes contain a higher proportion of videos that include false information, or “Fake News.” If that is the case, then the observed asymmetry in YouTube’s recommendations could be explained by the recommendation algorithm’s aversion to fake content and misinformation, rather than a bias towards left-leaning content. To investigate whether this is the case, we perform an analysis to determine the proportion of videos in each class which include information classified as False by political fact-checking websites. This analysis is repeated with two different thresholds, 0.8 and 0.9; the 0.8 threshold, for instance, indicates that a video would be classified as fake news if it has a similarity score of at least 0.8 with a headline that is labeled as fake according to the fact checking websites (see Methods for more details). The results of this analysis are reported in Table 1. Given a threshold of 0.9, only 0.0005% of videos were categorized as Fake News, with no significant difference between political classes. On the other hand, given a threshold of 0.8, we see 0.0537% of videos classified as Fake News, distributed uniformly across the different political classes. This finding suggests that the videos containing fake news are not concentrated in any one political class, and only form a negligible proportion of political videos. This finding suggests that YouTube’s left-leaning bias cannot be explained by the algorithm’s aversion to misinformation and fake news content.

**Table 1.** Proportion of videos under each political classification that had a semantic similarity score greater than 0.9 or 0.8 when compared against fake news headlines.

Political class	Percentage of videos labeled as fake news	
	Threshold = 0.9 (%)	Threshold = 0.8 (%)
Far Left	0	0.0441
Left	0.0005	0.0588
Center	0.0004	0.0479
Anti-Woke	0.001	0.0373
Right	0.0087	0.0785
Far Right	0.0007	0.0676
Total	0.0005	0.0537

Supplementary Tables 10–15 summarize the distribution of labeled recommendations across political classifications during this stage of the experiment. Moreover, Supplementary Figs. 1–6 illustrate the mean and 95% confidence intervals of the proportion of recommendations falling under each political classification after each video is watched during this stage of the experiment.

### Recommendation pathways

We continue our analysis with the third and final stage of the experiment, which explores recommendation pathways when a user watches the videos recommended to them, rather than a predetermined sequence of videos. In this stage, a user who has watched a sequence of 30 videos from one political class, and then another 30 videos from a different political class, will watch the top recommended video on their homepage. After a video is watched, the top 20 recommended videos to them will be collected, and then watch the video at the top of their home page. This process is repeated 30 times. Here, we are interested in exploring any transitions in the YouTube recommendation algorithm. In other words, we explore whether the algorithm continues to recommend videos from the political class watched in the second stage of the experiment, or whether it switches to a different political class.

**Fig. 5.**
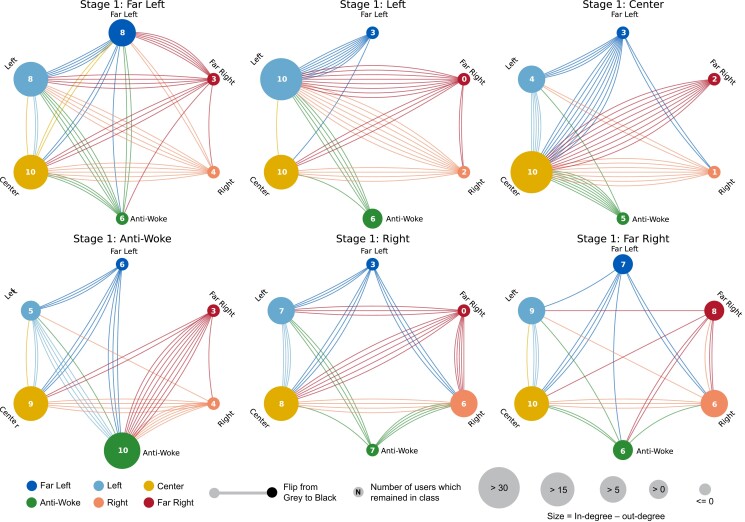
Recommendation pathways. Each subplot represents users who watched a particular political class in the first stage. Within each subplot, each vertex represents the political class watched in the second stage. Colored lines represent a switch from one class to another after 30 videos were watched in the third stage, where the color denotes the origin class from which the directed edge is emanating. The numeric value in each vertex represents the number of users which remained within the class (i.e. the number of self-loops). The size of each vertex corresponds to the difference in number of inbound edges and outbound edges. Larger vertices indicate that users are more likely to enter the vertex’s class, while smaller vertices indicate that users are more likely to escape the vertex class.

A summary of the transitions by the end of this stage can be seen in Fig. 5. In this figure, the title of each subplot corresponds to the political class of videos which users had watched in the first stage of the experiment. Within each subplot, each vertex corresponds to the class of videos watched in the second stage of the experiment, while colored lines correspond to transitions in recommendations after the third stage of the experiment, where the color represents the origin class from which the directed edge is emanating. The numeric value in each vertex represents the number of instances in which there was no transition in recommendations between the second and third stages of the experiment (i.e. the number of self-loops). Finally, the size of each vertex corresponds to the difference between the in-degree and out-degree of each vertex. Here, the size of each vertex can be thought of as its “gravitational pull,” where larger vertices both attract and keep more users within their class, while smaller vertices typically allow users to transition away from them.

Beginning with the case of users watching Center videos in the first stage of the experiment (the top-right subplot), we see that a majority of users return to Center after the conclusion of the third stage of the experiment, illustrating the algorithm’s tendency to return to the Center. Indeed, the Center political class had the strongest “gravitational pull” as evidenced by the fact that the yellow vertex is the largest in four out of the six subplots. The Left political class tended to have the second strongest pull, followed by the Anti-Woke, and Right classes. In contrast, the Far Left and Far Right had the weakest pull, further supporting our previous findings illustrating the algorithm’s tendency to pull away from political extremes. The distribution of labeled recommendations across political classifications during this stage of the experiment can be found in Supplementary Tables 16–21. Furthermore, Supplementary Figs. 7–12 illustrate the mean and 95% confidence intervals of the proportion of recommendations falling under each political classification after each video is watched during this stage of the experiment.

## Discussion

This study evaluates the YouTube recommendation algorithm’s propensity to build political filter bubbles, and the ability and speed by which one may escape them. As shown in our work, the recommendations made by the algorithm with regard to US political videos are asymmetric with a skew towards the Left, both with respect to the speed by which one may enter a political persona, as well as the difficulty in escaping these filter bubbles. This asymmetry exists even when users have not watched any videos, with baseline recommendations made to users also exhibiting more left-leaning videos than their right-leaning counterparts. Furthermore, we have shown that, in line with YouTube’s effort to reduce the amount of extremist content shown to users via the recommendation algorithm (37), users are indeed pulled away from the extremes on both sides of the political aisle. However, the strength of this pull is, again, not symmetrical, with users pulled away from the Far Right political ideology disproportionately stronger than those in Far Left. Our work extends previous literature on YouTube’s recommendation algorithm and supports the findings of several studies suggesting that the algorithm does not lead towards more extremist content (26, 29). We extend this literature by performing a controlled experiment to estimate the “gravitational pull” of each political class in a personalized environment, finding that not only do the recommendations made by the algorithm tend to pull away from political extremes, but it does so in a left-leaning manner. This is despite the fact that Far Right communities on YouTube are larger than corresponding Far Left ones (30).

YouTube’s recommendation algorithm’s left-leaning tilt in the US political context offers questions on the nature of how an ideal recommendation algorithm should perform. Naturally, recommendation algorithms which only offer content similar to what is being watched by the user may create echo chambers. Alternatives could include recommendation algorithms that pull users symmetrically towards the center, or one that offers a uniform distribution of videos across the political aisle. However, given the for-profit nature of YouTube and other similar social media platforms, it is unknown whether the companies behind them would be willing to adopt a different format of recommendation delivery if they do not drive engagement. Further studies may examine the utility of recommendation algorithms which adhere to user-customizable settings, where one can specify the proportion of videos they would be willing to see outside of their own interests or political views. Furthermore, future research may explore potential political biases which exist on different social media platforms on which users consume political content. According to a Pew Research study on news consumption via social media (3), TikTok and Twitch were the only two platforms on which the proportion of respondents who consume political video content grew from 2020 to 2021 among US adults. Understanding potential biases on such growing platforms, particularly due to their younger age demographic, is thus an interesting area of future inquiry.

Future work may also examine the nature of the categorical distribution of videos across the political spectrum. In our work, we find that videos in each political class followed distinct distributions with regard to their categorization on YouTube (e.g. “News & Politics,” “Education,” etc.). While we found that the majority of videos under both political extremes primarily fall under the “Education” category, the reasons behind this are not yet understood. Future work may examine both the psychological underpinnings of categorizing YouTube videos as “Educational,” as well as whether such categorization leads to increased engagement. Our work also focuses on the US-centric political zeitgeist, and as such, the degree to which our findings hold in different country-specific political contexts remains to be seen. Furthermore, while we run our experiment on the Android operating system and on a particular mobile device, namely the Xiaomi Redmi Go, one could explore the degree to which the operating system or type of device on which one consumes content on YouTube affects the types of recommendations they receive. Taken together, our study raises questions on whether recommendation algorithms should exhibit political biases, and the wide-reaching societal and political implications that such biases could entail given the vast audience of these platforms, and the power they hold in forming political opinion.

Supplementary material is available at *PNAS Nexus* online.

## Supplementary Material

pgad264_Supplementary_Data

## Data Availability

All of the data used in our analysis can be found at the following repository: https://github.com/comnetsAD/youtube-politics.
