# Supplementary material for: YouTube’s recommendation algorithm is left-leaning in the United States
Source: PNAS Nexus. 2023 Aug 14;2(8):pgad264. doi: 10.1093/pnasnexus/pgad264 (PMC10433241; doi:10.1093/pnasnexus/pgad264)
Supplement: pgad264_Supplementary_Data [file pgad264_supplementary_data.zip › PNASNEXUS-PNASNEXUS-2023-00588R-s01.pdf]

Supplementary Information for

YouTube's recommendation algorithm is left-leaning

Hazem Ibrahim[a], Nouar AlDahoul[a], Sangjin Lee[a], Talal Rahwan[a], Yasir Zaki[a*]

[a]Computer Science, New York University Abu Dhabi

[*]Corresponding author. E-mail: yasir.zaki@nyu.edu

This document is structured as follows:

- **Supplementary Tables** (*page 2*)
- **Supplementary Figures** (*page 69*)

Supplementary Tables

Baseline tables

Category	Count	Far Left	Left	Center	Anti-Woke	Right	Far Right	Un-labelled
Pets & Animals	66	0	0	0	0	0	0	66
Film & Animation	129	0	0	0	0	0	0	129
Education	299	7	1	1	0	2	2	286
People & Blogs	543	1	2	0	3	1	3	533
Music	272	1	1	0	0	0	2	268
Entertainment	383	0	1	2	0	0	0	380
News & Politics	112	1	19	33	0	4	0	55
Autos & Vehicles	51	0	0	0	0	0	0	51
Nonprofits & Activism	23	0	0	0	0	1	0	22
Sports	98	0	0	0	0	0	0	98
Comedy	74	0	4	0	1	0	0	69
Science & Technology	61	0	0	0	0	0	0	61
Howto & Style	91	0	0	0	0	0	0	91
Gaming	65	0	0	0	0	0	0	65
Travel & Events	38	0	0	0	0	0	0	38
**Total**	**2305**	**10**	**28**	**36**	**4**	**8**	**7**	**2212**

Table S1: **Baseline video counts.** The number of videos recommended to a new user within each category, as well as the number of videos falling under each political classification within each category.

	Far Left	Left	Center	Anti-Woke	Right	Far Right
Baseline	10	28	36	4	8	7
Dataset	106603	1401168	7162943	519252	1363191	1024750
Normalized Dataset	0.86	11.25	57.53	4.17	10.95	8.23
p value	1.09e-26					

Table S2: **Baseline Chi-Squared test**

Stage 1 tables (Entering a political persona)

The first table in this subsection details the Bhattacharyya Distance between the distribution of labelled recommended videos as users watch videos during the first stage of the experiment against the distribution of all videos in the entire labelled dataset. The remaining tables detail the distribution of political classifications for the labelled recommended videos as a new user watches 30 videos of a particular political classification.

Video Number	Far Left	Left	Center	Anti-Woke	Right	Far Right
1	0.43	0.3	0.04	0.45	0.17	0.21
2	0.46	0.3	0.06	0.42	0.21	0.27
3	0.52	0.3	0.04	0.5	0.23	0.22
4	0.6	0.38	0.04	0.57	0.21	0.23
5	0.53	0.44	0.07	0.49	0.2	0.24
6	0.56	0.33	0.06	0.56	0.16	0.29
7	0.63	0.39	0.05	0.55	0.19	0.37
8	0.63	0.33	0.06	0.54	0.17	0.4
9	0.73	0.36	0.03	0.48	0.16	0.31
10	0.71	0.33	0.05	0.44	0.2	0.39
11	0.74	0.32	0.07	0.5	0.17	0.33
12	0.74	0.37	0.07	0.54	0.17	0.27
13	0.71	0.35	0.06	0.52	0.19	0.23
14	0.62	0.32	0.07	0.6	0.19	0.27
15	0.61	0.33	0.07	0.61	0.19	0.37
16	0.66	0.36	0.06	0.51	0.22	0.28
17	0.62	0.36	0.07	0.51	0.2	0.22
18	0.97	0.36	0.07	0.61	0.24	0.23
19	0.73	0.32	0.07	0.58	0.2	0.27
20	0.77	0.31	0.07	0.59	0.22	0.25
21	0.58	0.37	0.05	0.53	0.18	0.25
22	0.69	0.29	0.07	0.52	0.14	0.31
23	0.51	0.35	0.08	0.58	0.17	0.27
24	0.57	0.39	0.08	0.63	0.16	0.29
25	0.54	0.34	0.07	0.56	0.14	0.25
26	0.58	0.35	0.07	0.55	0.13	0.26
27	0.54	0.36	0.08	0.56	0.14	0.22
28	0.59	0.33	0.07	0.55	0.15	0.2
29	0.59	0.33	0.08	0.54	0.16	0.23
30	0.53	0.31	0.05	0.51	0.17	0.2
Sum	18.69	10.28	1.88	16.1	5.43	8.13

Table S3: Bhattacharyya Distance between the distribution of labelled recommended videos as users watch videos during the first stage of the experiment against the distribution of all videos in the entire labelled dataset. The title of each column represents the classification of videos being watched.

Video Number	Far Left	Left	Center	Anti-Woke	Right	Far Right	Unlabelled
1	32.0	28.0	3.0	17.0	17.0	3.0	55.4
2	40.0	27.0	3.0	15.0	13.0	1.0	44.4
3	48.0	25.0	3.0	10.0	13.0	1.0	42.1
4	58.0	21.0	4.0	11.0	6.0	0.0	40.5
5	57.0	19.0	5.0	11.0	6.0	1.0	39.4
6	54.0	24.0	4.0	12.0	5.0	1.0	35.5
7	59.0	22.0	3.0	11.0	4.0	0.0	34.5
8	60.0	26.0	3.0	8.0	3.0	1.0	35.0
9	63.0	21.0	3.0	11.0	2.0	0.0	35.9
10	62.0	21.0	2.0	10.0	3.0	0.0	33.6
11	64.0	21.0	2.0	12.0	1.0	0.0	36.2
12	68.0	19.0	2.0	9.0	1.0	0.0	36.2
13	68.0	16.0	4.0	9.0	2.0	0.0	35.3
14	65.0	18.0	4.0	9.0	2.0	1.0	37.9
15	69.0	15.0	5.0	7.0	4.0	0.0	36.8
16	70.0	12.0	7.0	9.0	2.0	0.0	35.3
17	68.0	14.0	4.0	11.0	2.0	1.0	32.6
18	72.0	18.0	0.0	6.0	3.0	0.0	37.5
19	72.0	14.0	5.0	7.0	1.0	0.0	33.3
20	74.0	14.0	3.0	6.0	1.0	0.0	35.0
21	69.0	16.0	8.0	5.0	1.0	0.0	31.9
22	72.0	13.0	7.0	7.0	1.0	0.0	30.8
23	65.0	13.0	13.0	8.0	1.0	0.0	31.7
24	70.0	15.0	10.0	5.0	1.0	0.0	30.8
25	67.0	16.0	10.0	6.0	1.0	0.0	30.4
26	69.0	18.0	8.0	4.0	1.0	0.0	31.6
27	67.0	15.0	11.0	4.0	2.0	0.0	31.6
28	70.0	13.0	8.0	6.0	2.0	0.0	30.7
29	70.0	16.0	8.0	5.0	1.0	0.0	29.0
30	65.0	17.0	10.0	5.0	2.0	0.0	32.9

Table S4: Proportion of labelled videos falling under each political classification, as well as the proportion of unlabelled videos, as a new user begins to watch Far Left videos in the first stage of the experiment.

Video Number	Far Left	Left	Center	Anti-Woke	Right	Far Right	Unlabelled
1	1.0	76.0	15.0	5.0	2.0	1.0	55.6
2	0.0	77.0	15.0	6.0	2.0	1.0	46.8
3	1.0	77.0	13.0	4.0	4.0	1.0	43.6
4	1.0	81.0	11.0	5.0	3.0	0.0	37.9
5	0.0	85.0	9.0	5.0	1.0	0.0	40.7
6	1.0	79.0	11.0	5.0	4.0	0.0	39.2
7	2.0	83.0	10.0	4.0	2.0	0.0	33.0
8	0.0	80.0	15.0	2.0	2.0	0.0	34.4
9	1.0	80.0	14.0	3.0	2.0	0.0	33.8
10	1.0	81.0	12.0	3.0	1.0	1.0	30.2
11	0.0	82.0	14.0	2.0	2.0	0.0	36.1
12	0.0	84.0	12.0	2.0	2.0	0.0	33.6
13	0.0	83.0	12.0	3.0	2.0	0.0	31.8
14	0.0	83.0	13.0	2.0	1.0	0.0	33.9
15	0.0	80.0	16.0	3.0	1.0	0.0	30.7
16	1.0	83.0	11.0	2.0	2.0	0.0	27.5
17	0.0	85.0	12.0	1.0	2.0	0.0	31.4
18	0.0	84.0	14.0	2.0	0.0	0.0	31.6
19	0.0	82.0	14.0	2.0	2.0	0.0	33.8
20	1.0	81.0	14.0	2.0	2.0	0.0	34.0
21	0.0	87.0	11.0	1.0	2.0	0.0	29.4
22	0.0	79.0	18.0	0.0	2.0	0.0	31.1
23	0.0	84.0	14.0	0.0	2.0	0.0	29.9
24	0.0	86.0	12.0	1.0	0.0	0.0	30.8
25	0.0	83.0	15.0	0.0	1.0	0.0	32.3
26	1.0	81.0	17.0	0.0	0.0	0.0	33.2
27	0.0	83.0	16.0	0.0	1.0	0.0	30.3
28	0.0	80.0	18.0	0.0	1.0	0.0	29.6
29	0.0	80.0	19.0	0.0	1.0	0.0	31.1
30	0.0	80.0	18.0	1.0	2.0	0.0	29.6

Table S5: Proportion of labelled videos falling under each political classification, as well as the proportion of unlabelled videos, as a new user begins to watch Left videos in the first stage of the experiment.

Video Number	Far Left	Left	Center	Anti-Woke	Right	Far Right	Unlabelled
1	0.0	22.0	66.0	4.0	7.0	1.0	57.0
2	1.0	21.0	56.0	14.0	8.0	0.0	49.3
3	1.0	22.0	57.0	10.0	10.0	1.0	46.6
4	1.0	19.0	60.0	13.0	6.0	1.0	41.8
5	0.0	29.0	52.0	12.0	6.0	0.0	41.2
6	0.0	23.0	58.0	12.0	7.0	0.0	39.6
7	0.0	25.0	58.0	10.0	6.0	0.0	35.4
8	1.0	21.0	61.0	10.0	7.0	0.0	37.2
9	1.0	19.0	60.0	8.0	11.0	1.0	35.1
10	1.0	19.0	64.0	9.0	7.0	0.0	33.0
11	1.0	19.0	70.0	7.0	3.0	0.0	36.8
12	0.0	25.0	64.0	4.0	6.0	0.0	34.5
13	1.0	21.0	68.0	5.0	6.0	0.0	32.1
14	0.0	16.0	72.0	7.0	4.0	0.0	31.5
15	0.0	21.0	72.0	3.0	4.0	0.0	30.8
16	0.0	19.0	73.0	3.0	6.0	0.0	28.6
17	0.0	19.0	74.0	4.0	3.0	0.0	32.7
18	0.0	21.0	74.0	1.0	4.0	0.0	28.3
19	0.0	23.0	69.0	3.0	4.0	0.0	28.0
20	0.0	19.0	72.0	4.0	5.0	0.0	29.3
21	0.0	18.0	75.0	3.0	4.0	1.0	26.8
22	0.0	16.0	77.0	3.0	4.0	0.0	29.9
23	0.0	16.0	78.0	2.0	3.0	0.0	26.9
24	0.0	15.0	80.0	4.0	2.0	0.0	29.0
25	0.0	18.0	77.0	1.0	4.0	0.0	26.0
26	0.0	17.0	76.0	1.0	5.0	0.0	28.9
27	0.0	17.0	77.0	2.0	4.0	0.0	28.0
28	0.0	20.0	74.0	1.0	5.0	0.0	27.2
29	0.0	19.0	76.0	2.0	3.0	0.0	28.0
30	0.0	15.0	78.0	2.0	4.0	0.0	26.3

Table S6: Proportion of labelled videos falling under each political classification, as well as the proportion of unlabelled videos, as a new user begins to watch Center videos in the first stage of the experiment.

Video Number	Far Left	Left	Center	Anti-Woke	Right	Far Right	Unlabelled
1	2.0	9.0	5.0	66.0	17.0	1.0	54.9
2	0.0	10.0	6.0	69.0	12.0	1.0	47.4
3	1.0	2.0	6.0	73.0	17.0	2.0	47.7
4	0.0	4.0	2.0	75.0	18.0	0.0	45.8
5	2.0	7.0	5.0	73.0	13.0	0.0	44.8
6	1.0	3.0	3.0	74.0	18.0	1.0	42.5
7	0.0	5.0	2.0	73.0	18.0	2.0	44.6
8	2.0	6.0	3.0	73.0	15.0	1.0	41.9
9	1.0	5.0	5.0	78.0	11.0	1.0	40.0
10	1.0	8.0	6.0	72.0	11.0	2.0	41.5
11	1.0	7.0	5.0	78.0	7.0	2.0	41.5
12	0.0	4.0	5.0	79.0	11.0	1.0	42.7
13	0.0	6.0	5.0	77.0	10.0	2.0	40.7
14	0.0	4.0	3.0	81.0	12.0	1.0	40.7
15	1.0	6.0	4.0	82.0	8.0	0.0	38.6
16	2.0	4.0	5.0	79.0	9.0	1.0	40.8
17	1.0	4.0	5.0	79.0	10.0	1.0	37.0
18	1.0	2.0	4.0	82.0	9.0	1.0	41.7
19	0.0	4.0	4.0	83.0	8.0	1.0	39.0
20	0.0	4.0	5.0	81.0	9.0	0.0	39.0
21	1.0	4.0	5.0	83.0	6.0	1.0	38.0
22	0.0	4.0	6.0	80.0	10.0	0.0	38.7
23	0.0	5.0	5.0	82.0	8.0	0.0	39.2
24	1.0	5.0	3.0	82.0	10.0	0.0	37.4
25	0.0	3.0	7.0	81.0	9.0	0.0	38.3
26	1.0	4.0	4.0	82.0	8.0	1.0	36.2
27	0.0	2.0	5.0	84.0	7.0	1.0	35.8
28	0.0	5.0	7.0	80.0	8.0	0.0	40.2
29	0.0	4.0	6.0	80.0	9.0	0.0	39.0
30	1.0	7.0	5.0	78.0	9.0	1.0	40.5

Table S7: Proportion of labelled videos falling under each political classification, as well as the proportion of unlabelled videos, as a new user begins to watch Anti-Woke videos in the first stage of the experiment.

Video Number	Far Left	Left	Center	Anti-Woke	Right	Far Right	Unlabelled
1	0.0	17.0	17.0	9.0	55.0	3.0	55.4
2	1.0	16.0	12.0	3.0	63.0	5.0	53.3
3	1.0	8.0	14.0	5.0	69.0	3.0	50.0
4	0.0	12.0	13.0	7.0	63.0	5.0	43.8
5	0.0	9.0	14.0	6.0	65.0	6.0	45.2
6	0.0	10.0	18.0	9.0	57.0	7.0	40.0
7	0.0	6.0	16.0	7.0	63.0	8.0	38.2
8	0.0	7.0	18.0	7.0	61.0	6.0	40.2
9	0.0	11.0	18.0	8.0	58.0	5.0	40.0
10	0.0	6.0	17.0	13.0	61.0	2.0	43.9
11	0.0	6.0	20.0	9.0	61.0	4.0	40.0
12	1.0	7.0	19.0	8.0	62.0	4.0	37.6
13	0.0	7.0	16.0	8.0	64.0	4.0	39.6
14	0.0	7.0	19.0	7.0	63.0	4.0	38.4
15	0.0	5.0	20.0	8.0	63.0	4.0	37.9
16	0.0	10.0	14.0	5.0	69.0	3.0	37.1
17	1.0	8.0	18.0	7.0	64.0	2.0	38.7
18	0.0	9.0	12.0	6.0	69.0	3.0	37.1
19	0.0	7.0	19.0	7.0	65.0	2.0	36.0
20	0.0	9.0	13.0	6.0	68.0	4.0	38.4
21	1.0	7.0	22.0	5.0	63.0	1.0	35.1
22	0.0	9.0	26.0	7.0	57.0	1.0	37.4
23	0.0	9.0	25.0	4.0	61.0	1.0	37.4
24	0.0	9.0	23.0	6.0	60.0	2.0	37.5
25	0.0	8.0	26.0	4.0	59.0	2.0	34.2
26	0.0	10.0	29.0	4.0	55.0	1.0	35.3
27	1.0	8.0	27.0	4.0	59.0	1.0	38.1
28	0.0	10.0	25.0	4.0	59.0	1.0	35.1
29	1.0	12.0	24.0	3.0	60.0	1.0	31.5
30	0.0	12.0	23.0	6.0	59.0	1.0	35.1

Table S8: Proportion of labelled videos falling under each political classification, as well as the proportion of unlabelled videos, as a new user begins to watch Right videos in the first stage of the experiment.

Video Number	Far Left	Left	Center	Anti-Woke	Right	Far Right	Unlabelled
1	0.0	10.0	11.0	9.0	32.0	38.0	63.8
2	1.0	7.0	7.0	7.0	30.0	49.0	60.6
3	1.0	9.0	10.0	8.0	31.0	41.0	58.8
4	2.0	7.0	10.0	4.0	34.0	42.0	58.1
5	1.0	7.0	8.0	6.0	31.0	48.0	53.3
6	0.0	6.0	6.0	4.0	31.0	53.0	54.8
7	0.0	2.0	6.0	11.0	32.0	50.0	52.9
8	0.0	4.0	4.0	4.0	31.0	57.0	51.6
9	0.0	6.0	8.0	4.0	24.0	59.0	52.2
10	0.0	3.0	5.0	5.0	28.0	60.0	51.0
11	0.0	5.0	5.0	10.0	24.0	56.0	55.5
12	1.0	8.0	6.0	2.0	30.0	53.0	55.8
13	0.0	3.0	10.0	9.0	22.0	57.0	53.1
14	1.0	8.0	6.0	7.0	20.0	58.0	57.4
15	1.0	3.0	3.0	16.0	25.0	52.0	56.8
16	0.0	8.0	6.0	8.0	22.0	56.0	54.5
17	0.0	8.0	12.0	5.0	17.0	57.0	55.7
18	1.0	5.0	9.0	6.0	23.0	56.0	56.1
19	1.0	5.0	6.0	6.0	22.0	60.0	53.4
20	1.0	2.0	9.0	9.0	20.0	59.0	57.3
21	1.0	5.0	8.0	5.0	11.0	71.0	52.8
22	1.0	9.0	3.0	6.0	19.0	61.0	53.5
23	1.0	5.0	7.0	7.0	21.0	60.0	48.4
24	1.0	4.0	7.0	5.0	19.0	64.0	52.8
25	0.0	7.0	8.0	6.0	20.0	60.0	51.5
26	0.0	5.0	9.0	6.0	17.0	63.0	50.2
27	0.0	9.0	10.0	6.0	22.0	53.0	51.3
28	1.0	6.0	12.0	7.0	20.0	54.0	51.6
29	1.0	5.0	9.0	4.0	22.0	59.0	50.6
30	0.0	5.0	14.0	5.0	18.0	57.0	50.6

Table S9: Proportion of labelled videos falling under each political classification, as well as the proportion of unlabelled videos, as a new user begins to watch Far Right videos in the first stage of the experiment.

Stage 2 tables (Escaping a political persona)

The tables in this subsection detail the distribution of labelled videos across political classifications as a user begins to watch a sequence of 30 videos falling under a new political classification (labelled as New Class in the tables). These users had already watched 30 videos under a particular political classification (labelled as Original class in the tables) prior to watching the next 30 videos.

Original Class	New Class	Video Number	Far Left	Left	Center	Anti-Woke	Right	Far Right
FL	FL	1	82.0	9.0	4.0	3.0	1.0	0.0
FL	FL	2	72.0	17.0	8.0	2.0	0.0	0.0
FL	FL	3	73.0	17.0	3.0	7.0	0.0	0.0
FL	FL	4	75.0	13.0	8.0	2.0	2.0	0.0
FL	FL	5	85.0	9.0	3.0	2.0	2.0	0.0
FL	FL	6	75.0	15.0	4.0	4.0	3.0	0.0
FL	FL	7	76.0	18.0	2.0	2.0	2.0	0.0
FL	FL	8	79.0	13.0	5.0	4.0	0.0	0.0
FL	FL	9	80.0	11.0	3.0	6.0	0.0	0.0
FL	FL	10	78.0	11.0	4.0	6.0	1.0	0.0
FL	FL	11	77.0	15.0	6.0	1.0	1.0	0.0
FL	FL	12	81.0	12.0	6.0	1.0	0.0	0.0
FL	FL	13	75.0	13.0	5.0	5.0	1.0	0.0
FL	FL	14	78.0	13.0	3.0	4.0	2.0	0.0
FL	FL	15	77.0	16.0	3.0	4.0	0.0	0.0
FL	FL	16	76.0	14.0	2.0	5.0	3.0	1.0
FL	FL	17	83.0	9.0	5.0	3.0	0.0	0.0
FL	FL	18	78.0	9.0	8.0	3.0	1.0	0.0
FL	FL	19	77.0	16.0	2.0	5.0	1.0	0.0
FL	FL	20	82.0	9.0	2.0	7.0	1.0	0.0
FL	FL	21	77.0	11.0	6.0	5.0	1.0	0.0
FL	FL	22	77.0	12.0	7.0	4.0	0.0	1.0
FL	FL	23	80.0	7.0	6.0	4.0	2.0	0.0
FL	FL	24	71.0	14.0	7.0	8.0	0.0	0.0
FL	FL	25	74.0	16.0	7.0	2.0	2.0	0.0
FL	FL	26	77.0	10.0	10.0	1.0	1.0	0.0
FL	FL	27	73.0	18.0	4.0	2.0	2.0	0.0
FL	FL	28	77.0	16.0	5.0	1.0	0.0	1.0
FL	FL	29	73.0	19.0	2.0	4.0	2.0	0.0
FL	FL	30	79.0	14.0	4.0	3.0	1.0	0.0
FL	L	1	52.0	33.0	8.0	6.0	1.0	0.0

FL	L	2	49.0	38.0	10.0	1.0	1.0	0.0
FL	L	3	40.0	**45.0**	11.0	4.0	0.0	0.0
FL	L	4	30.0	55.0	10.0	3.0	2.0	0.0
FL	L	5	34.0	52.0	11.0	2.0	0.0	1.0
FL	L	6	31.0	59.0	8.0	3.0	0.0	0.0
FL	L	7	30.0	58.0	7.0	4.0	0.0	1.0
FL	L	8	30.0	57.0	8.0	3.0	1.0	1.0
FL	L	9	27.0	62.0	5.0	4.0	2.0	1.0
FL	L	10	25.0	59.0	8.0	6.0	2.0	0.0
FL	L	11	19.0	65.0	13.0	3.0	1.0	0.0
FL	L	12	22.0	63.0	9.0	2.0	5.0	0.0
FL	L	13	20.0	70.0	7.0	4.0	0.0	0.0
FL	L	14	20.0	67.0	10.0	2.0	0.0	1.0
FL	L	15	18.0	62.0	17.0	3.0	0.0	0.0
FL	L	16	16.0	72.0	7.0	3.0	2.0	0.0
FL	L	17	21.0	62.0	15.0	1.0	1.0	0.0
FL	L	18	20.0	64.0	14.0	2.0	0.0	0.0
FL	L	19	10.0	67.0	21.0	1.0	2.0	0.0
FL	L	20	14.0	69.0	11.0	3.0	2.0	0.0
FL	L	21	16.0	67.0	14.0	2.0	1.0	0.0
FL	L	22	21.0	58.0	14.0	4.0	4.0	0.0
FL	L	23	16.0	71.0	12.0	2.0	0.0	0.0
FL	L	24	11.0	68.0	17.0	3.0	1.0	0.0
FL	L	25	16.0	66.0	17.0	1.0	0.0	0.0
FL	L	26	21.0	61.0	15.0	4.0	0.0	0.0
FL	L	27	17.0	70.0	14.0	0.0	0.0	0.0
FL	L	28	18.0	62.0	17.0	1.0	1.0	1.0
FL	L	29	13.0	67.0	17.0	1.0	2.0	0.0
FL	L	30	16.0	67.0	15.0	2.0	0.0	0.0
FL	C	1	29.0	21.0	35.0	8.0	8.0	0.0
FL	C	2	32.0	22.0	**38.0**	4.0	4.0	1.0
FL	C	3	18.0	27.0	42.0	9.0	4.0	0.0
FL	C	4	21.0	21.0	49.0	6.0	2.0	0.0
FL	C	5	32.0	14.0	46.0	6.0	2.0	0.0
FL	C	6	21.0	21.0	47.0	8.0	2.0	0.0
FL	C	7	21.0	13.0	53.0	10.0	4.0	0.0
FL	C	8	19.0	21.0	48.0	7.0	4.0	1.0
FL	C	9	13.0	22.0	56.0	5.0	4.0	0.0
FL	C	10	15.0	21.0	51.0	10.0	2.0	0.0
FL	C	11	12.0	16.0	62.0	7.0	2.0	0.0

FL	C	12	10.0	23.0	59.0	4.0	4.0	0.0
FL	C	13	8.0	20.0	64.0	4.0	3.0	0.0
FL	C	14	5.0	27.0	63.0	2.0	3.0	0.0
FL	C	15	9.0	29.0	55.0	6.0	1.0	0.0
FL	C	16	10.0	24.0	62.0	1.0	4.0	0.0
FL	C	17	4.0	22.0	63.0	6.0	6.0	0.0
FL	C	18	7.0	29.0	60.0	3.0	1.0	0.0
FL	C	19	10.0	24.0	62.0	2.0	2.0	0.0
FL	C	20	7.0	15.0	74.0	2.0	1.0	0.0
FL	C	21	8.0	19.0	66.0	2.0	5.0	0.0
FL	C	22	9.0	20.0	61.0	4.0	6.0	0.0
FL	C	23	4.0	14.0	74.0	3.0	4.0	0.0
FL	C	24	14.0	11.0	70.0	3.0	2.0	0.0
FL	C	25	10.0	23.0	63.0	0.0	4.0	0.0
FL	C	26	12.0	20.0	64.0	1.0	3.0	0.0
FL	C	27	9.0	22.0	61.0	4.0	5.0	0.0
FL	C	28	8.0	18.0	72.0	2.0	0.0	0.0
FL	C	29	7.0	17.0	71.0	3.0	3.0	0.0
FL	C	30	5.0	20.0	67.0	4.0	3.0	0.0
FL	AW	1	41.0	19.0	5.0	31.0	3.0	1.0
FL	AW	2	41.0	15.0	6.0	37.0	0.0	0.0
FL	AW	3	34.0	12.0	4.0	**50.0**	0.0	0.0
FL	AW	4	36.0	11.0	7.0	39.0	6.0	1.0
FL	AW	5	35.0	11.0	2.0	43.0	7.0	2.0
FL	AW	6	33.0	20.0	5.0	39.0	3.0	0.0
FL	AW	7	31.0	13.0	8.0	44.0	4.0	0.0
FL	AW	8	26.0	15.0	1.0	50.0	6.0	1.0
FL	AW	9	30.0	12.0	1.0	51.0	6.0	0.0
FL	AW	10	36.0	9.0	1.0	53.0	1.0	0.0
FL	AW	11	28.0	5.0	2.0	59.0	6.0	0.0
FL	AW	12	33.0	9.0	9.0	43.0	5.0	2.0
FL	AW	13	28.0	9.0	6.0	49.0	5.0	2.0
FL	AW	14	20.0	7.0	6.0	64.0	2.0	1.0
FL	AW	15	30.0	8.0	6.0	51.0	5.0	1.0
FL	AW	16	18.0	16.0	8.0	55.0	2.0	0.0
FL	AW	17	24.0	12.0	4.0	54.0	6.0	0.0
FL	AW	18	22.0	12.0	5.0	57.0	3.0	0.0
FL	AW	19	24.0	9.0	9.0	53.0	3.0	1.0
FL	AW	20	27.0	7.0	5.0	52.0	8.0	1.0
FL	AW	21	27.0	8.0	4.0	57.0	3.0	1.0

FL	AW	22	30.0	5.0	6.0	54.0	3.0	2.0
FL	AW	23	33.0	10.0	1.0	53.0	1.0	1.0
FL	AW	24	27.0	8.0	2.0	58.0	4.0	0.0
FL	AW	25	29.0	7.0	3.0	53.0	7.0	1.0
FL	AW	26	23.0	6.0	2.0	66.0	3.0	0.0
FL	AW	27	27.0	10.0	5.0	52.0	6.0	0.0
FL	AW	28	26.0	7.0	10.0	54.0	2.0	0.0
FL	AW	29	24.0	4.0	4.0	61.0	7.0	0.0
FL	AW	30	30.0	8.0	1.0	57.0	4.0	0.0
FL	R	1	47.0	16.0	11.0	4.0	22.0	0.0
FL	R	2	43.0	12.0	8.0	9.0	25.0	3.0
FL	R	3	47.0	19.0	8.0	6.0	20.0	0.0
FL	R	4	42.0	10.0	12.0	5.0	30.0	1.0
FL	R	5	34.0	15.0	7.0	10.0	34.0	1.0
FL	R	6	33.0	15.0	10.0	8.0	33.0	1.0
FL	R	7	37.0	12.0	11.0	11.0	29.0	0.0
FL	R	8	31.0	14.0	7.0	10.0	**36.0**	2.0
FL	R	9	32.0	15.0	6.0	9.0	39.0	0.0
FL	R	10	27.0	12.0	8.0	8.0	43.0	1.0
FL	R	11	22.0	17.0	12.0	5.0	42.0	1.0
FL	R	12	22.0	13.0	15.0	8.0	43.0	0.0
FL	R	13	22.0	16.0	13.0	11.0	37.0	1.0
FL	R	14	24.0	11.0	12.0	7.0	44.0	1.0
FL	R	15	24.0	14.0	11.0	4.0	46.0	1.0
FL	R	16	20.0	12.0	13.0	8.0	47.0	0.0
FL	R	17	22.0	9.0	19.0	3.0	46.0	1.0
FL	R	18	27.0	7.0	12.0	11.0	43.0	1.0
FL	R	19	18.0	12.0	18.0	3.0	49.0	0.0
FL	R	20	20.0	12.0	18.0	2.0	46.0	1.0
FL	R	21	16.0	14.0	14.0	5.0	51.0	1.0
FL	R	22	21.0	15.0	20.0	6.0	39.0	0.0
FL	R	23	23.0	10.0	17.0	8.0	41.0	0.0
FL	R	24	24.0	11.0	19.0	3.0	44.0	0.0
FL	R	25	19.0	10.0	22.0	7.0	41.0	1.0
FL	R	26	23.0	17.0	24.0	2.0	33.0	0.0
FL	R	27	24.0	10.0	26.0	2.0	38.0	0.0
FL	R	28	15.0	15.0	22.0	2.0	44.0	1.0
FL	R	29	19.0	13.0	23.0	3.0	40.0	1.0
FL	R	30	19.0	11.0	28.0	4.0	38.0	0.0
FL	FR	1	55.0	17.0	5.0	7.0	7.0	10.0

FL	FR	2	59.0	9.0	6.0	9.0	6.0	11.0
FL	FR	3	61.0	16.0	5.0	4.0	5.0	9.0
FL	FR	4	55.0	18.0	3.0	5.0	3.0	16.0
FL	FR	5	54.0	17.0	4.0	7.0	2.0	16.0
FL	FR	6	44.0	14.0	1.0	8.0	7.0	25.0
FL	FR	7	37.0	8.0	5.0	11.0	6.0	32.0
FL	FR	8	40.0	12.0	6.0	12.0	2.0	27.0
FL	FR	9	45.0	14.0	3.0	6.0	7.0	25.0
FL	FR	10	37.0	12.0	5.0	11.0	12.0	23.0
FL	FR	11	49.0	12.0	3.0	4.0	8.0	25.0
FL	FR	12	32.0	10.0	6.0	8.0	11.0	32.0
FL	FR	13	44.0	7.0	7.0	7.0	7.0	26.0
FL	FR	14	30.0	11.0	5.0	17.0	8.0	29.0
FL	FR	15	40.0	6.0	9.0	11.0	13.0	21.0
FL	FR	16	33.0	19.0	9.0	15.0	5.0	19.0
FL	FR	17	33.0	14.0	4.0	9.0	9.0	31.0
FL	FR	18	29.0	12.0	2.0	15.0	10.0	32.0
FL	FR	19	39.0	16.0	5.0	7.0	4.0	29.0
FL	FR	20	31.0	3.0	12.0	3.0	14.0	37.0
FL	FR	21	40.0	9.0	4.0	2.0	13.0	32.0
FL	FR	22	35.0	10.0	6.0	10.0	8.0	31.0
FL	FR	23	39.0	6.0	5.0	3.0	14.0	33.0
FL	FR	24	27.0	16.0	6.0	10.0	6.0	35.0
FL	FR	25	37.0	10.0	7.0	7.0	4.0	35.0
FL	FR	26	27.0	16.0	7.0	11.0	4.0	36.0
FL	FR	27	34.0	12.0	12.0	5.0	12.0	24.0
FL	FR	28	36.0	7.0	10.0	12.0	7.0	29.0
FL	FR	29	38.0	12.0	8.0	8.0	6.0	28.0
FL	FR	30	33.0	6.0	8.0	8.0	6.0	**39.0**

Table S 10: Proportion of labelled videos falling under each political classification as a Far Left user begins to watch videos of a new political classification in the second stage of the experiment. Percentages highlighted in bold denote the video at which the proportion of videos labelled under the new class exceeded that of the original class.

**Original Class**	**New Class**	**Video Number**	**Far Left**	**Left**	**Center**	**Anti-Woke**	**Right**	**Far Right**
L	FL	1	22.0	69.0	6.0	1.0	3.0	0.0
L	FL	2	26.0	63.0	9.0	2.0	1.0	0.0
L	FL	3	28.0	59.0	12.0	2.0	0.0	0.0

L	FL	4	35.0	57.0	6.0	2.0	0.0	0.0
L	FL	5	28.0	60.0	8.0	4.0	1.0	0.0
L	FL	6	34.0	55.0	6.0	3.0	3.0	0.0
L	FL	7	38.0	51.0	6.0	1.0	4.0	0.0
L	FL	8	50.0	45.0	4.0	1.0	1.0	0.0
L	FL	9	44.0	47.0	6.0	2.0	1.0	0.0
L	FL	10	50.0	44.0	3.0	4.0	0.0	0.0
L	FL	11	35.0	53.0	8.0	3.0	1.0	0.0
L	FL	12	42.0	43.0	10.0	3.0	2.0	0.0
L	FL	13	48.0	42.0	5.0	3.0	1.0	0.0
L	FL	14	52.0	44.0	3.0	0.0	1.0	0.0
L	FL	15	52.0	41.0	5.0	0.0	1.0	0.0
L	FL	16	52.0	37.0	7.0	4.0	1.0	0.0
L	FL	17	50.0	41.0	5.0	5.0	0.0	0.0
L	FL	18	54.0	39.0	4.0	2.0	2.0	0.0
L	FL	19	46.0	45.0	1.0	6.0	3.0	0.0
L	FL	20	45.0	46.0	4.0	3.0	2.0	1.0
L	FL	21	63.0	29.0	4.0	2.0	2.0	0.0
L	FL	22	47.0	41.0	6.0	5.0	1.0	0.0
L	FL	23	46.0	40.0	10.0	3.0	1.0	1.0
L	FL	24	53.0	39.0	7.0	1.0	0.0	0.0
L	FL	25	49.0	40.0	8.0	3.0	0.0	0.0
L	FL	26	50.0	42.0	7.0	0.0	1.0	0.0
L	FL	27	42.0	45.0	8.0	2.0	2.0	0.0
L	FL	28	**47.0**	41.0	9.0	1.0	1.0	0.0
L	FL	29	44.0	44.0	11.0	1.0	0.0	0.0
L	FL	30	56.0	34.0	10.0	0.0	0.0	0.0
L	L	1	0.0	84.0	13.0	1.0	2.0	0.0
L	L	2	0.0	89.0	11.0	0.0	0.0	0.0
L	L	3	0.0	79.0	16.0	1.0	4.0	0.0
L	L	4	0.0	81.0	17.0	0.0	3.0	0.0
L	L	5	1.0	85.0	13.0	0.0	1.0	0.0
L	L	6	0.0	90.0	8.0	2.0	0.0	0.0
L	L	7	0.0	88.0	10.0	0.0	2.0	0.0
L	L	8	0.0	86.0	13.0	1.0	0.0	0.0
L	L	9	0.0	86.0	12.0	1.0	1.0	0.0
L	L	10	1.0	88.0	11.0	1.0	0.0	0.0
L	L	11	0.0	88.0	10.0	1.0	1.0	0.0
L	L	12	0.0	88.0	10.0	1.0	1.0	0.0
L	L	13	1.0	93.0	6.0	0.0	1.0	0.0

L	L	14	1.0	85.0	13.0	0.0	2.0	0.0
L	L	15	0.0	88.0	12.0	0.0	0.0	0.0
L	L	16	0.0	89.0	10.0	0.0	2.0	0.0
L	L	17	0.0	89.0	10.0	0.0	1.0	0.0
L	L	18	0.0	91.0	8.0	1.0	1.0	0.0
L	L	19	0.0	90.0	8.0	2.0	1.0	0.0
L	L	20	0.0	90.0	9.0	0.0	1.0	0.0
L	L	21	0.0	89.0	9.0	2.0	0.0	0.0
L	L	22	0.0	89.0	9.0	1.0	1.0	0.0
L	L	23	1.0	82.0	16.0	0.0	2.0	0.0
L	L	24	0.0	86.0	13.0	0.0	1.0	0.0
L	L	25	0.0	86.0	14.0	0.0	0.0	0.0
L	L	26	0.0	82.0	18.0	0.0	1.0	0.0
L	L	27	0.0	81.0	17.0	0.0	2.0	0.0
L	L	28	0.0	78.0	20.0	1.0	1.0	0.0
L	L	29	0.0	80.0	20.0	0.0	0.0	0.0
L	L	30	0.0	85.0	14.0	1.0	0.0	0.0
L	C	1	0.0	56.0	37.0	4.0	4.0	0.0
L	C	2	0.0	51.0	42.0	3.0	4.0	0.0
L	C	3	0.0	51.0	40.0	5.0	4.0	0.0
L	C	4	0.0	60.0	33.0	4.0	3.0	0.0
L	C	5	1.0	38.0	57.0	3.0	1.0	0.0
L	C	6	0.0	46.0	47.0	3.0	4.0	0.0
L	C	7	0.0	48.0	44.0	3.0	5.0	0.0
L	C	8	1.0	44.0	43.0	5.0	7.0	0.0
L	C	9	0.0	42.0	**49.0**	4.0	5.0	0.0
L	C	10	1.0	44.0	50.0	3.0	3.0	0.0
L	C	11	1.0	37.0	56.0	2.0	5.0	0.0
L	C	12	0.0	41.0	55.0	1.0	3.0	0.0
L	C	13	0.0	40.0	58.0	1.0	1.0	0.0
L	C	14	0.0	39.0	55.0	3.0	3.0	0.0
L	C	15	1.0	41.0	54.0	3.0	2.0	0.0
L	C	16	0.0	43.0	51.0	3.0	3.0	0.0
L	C	17	0.0	38.0	59.0	0.0	4.0	0.0
L	C	18	0.0	48.0	49.0	1.0	3.0	0.0
L	C	19	0.0	44.0	53.0	1.0	2.0	0.0
L	C	20	0.0	32.0	65.0	0.0	3.0	0.0
L	C	21	0.0	35.0	62.0	2.0	2.0	0.0
L	C	22	0.0	35.0	63.0	1.0	1.0	0.0
L	C	23	0.0	37.0	62.0	0.0	1.0	0.0

L	C	24	0.0	41.0	54.0	1.0	4.0	1.0
L	C	25	0.0	37.0	61.0	1.0	1.0	0.0
L	C	26	0.0	43.0	55.0	2.0	0.0	0.0
L	C	27	0.0	34.0	63.0	2.0	1.0	0.0
L	C	28	0.0	38.0	58.0	2.0	3.0	0.0
L	C	29	0.0	35.0	59.0	1.0	6.0	0.0
L	C	30	0.0	41.0	58.0	1.0	0.0	0.0
L	AW	1	0.0	63.0	9.0	28.0	0.0	0.0
L	AW	2	0.0	54.0	8.0	34.0	4.0	0.0
L	AW	3	0.0	59.0	4.0	32.0	4.0	0.0
L	AW	4	0.0	52.0	13.0	33.0	3.0	0.0
L	AW	5	1.0	53.0	7.0	35.0	4.0	0.0
L	AW	6	2.0	48.0	5.0	38.0	7.0	0.0
L	AW	7	4.0	40.0	6.0	48.0	2.0	0.0
L	AW	8	1.0	38.0	11.0	46.0	5.0	0.0
L	AW	9	0.0	37.0	5.0	49.0	9.0	0.0
L	AW	10	0.0	36.0	7.0	51.0	5.0	1.0
L	AW	11	1.0	40.0	8.0	46.0	5.0	0.0
L	AW	12	1.0	38.0	10.0	50.0	1.0	0.0
L	AW	13	0.0	39.0	8.0	47.0	4.0	1.0
L	AW	14	1.0	46.0	8.0	45.0	0.0	0.0
L	AW	15	3.0	40.0	3.0	50.0	3.0	0.0
L	AW	16	0.0	38.0	10.0	44.0	7.0	1.0
L	AW	17	0.0	39.0	4.0	50.0	7.0	0.0
L	AW	18	0.0	24.0	10.0	58.0	6.0	1.0
L	AW	19	0.0	47.0	5.0	44.0	3.0	1.0
L	AW	20	2.0	40.0	8.0	**42.0**	7.0	0.0
L	AW	21	1.0	28.0	8.0	53.0	9.0	0.0
L	AW	22	0.0	31.0	8.0	57.0	4.0	0.0
L	AW	23	1.0	35.0	9.0	52.0	3.0	0.0
L	AW	24	0.0	30.0	7.0	58.0	5.0	0.0
L	AW	25	0.0	37.0	8.0	51.0	4.0	0.0
L	AW	26	0.0	31.0	1.0	61.0	7.0	0.0
L	AW	27	3.0	33.0	6.0	50.0	8.0	0.0
L	AW	28	0.0	41.0	7.0	43.0	9.0	0.0
L	AW	29	0.0	28.0	11.0	55.0	5.0	1.0
L	AW	30	0.0	35.0	4.0	55.0	6.0	0.0
L	R	1	0.0	65.0	14.0	2.0	19.0	0.0
L	R	2	0.0	51.0	9.0	3.0	36.0	2.0
L	R	3	0.0	60.0	19.0	0.0	19.0	2.0

L	R	4	0.0	49.0	16.0	3.0	31.0	0.0
L	R	5	0.0	55.0	19.0	2.0	23.0	2.0
L	R	6	0.0	49.0	17.0	2.0	33.0	0.0
L	R	7	0.0	50.0	17.0	3.0	30.0	0.0
L	R	8	1.0	49.0	15.0	4.0	31.0	0.0
L	R	9	1.0	45.0	12.0	3.0	39.0	0.0
L	R	10	0.0	44.0	20.0	2.0	32.0	1.0
L	R	11	0.0	51.0	12.0	4.0	32.0	1.0
L	R	12	0.0	38.0	22.0	2.0	39.0	0.0
L	R	13	0.0	47.0	20.0	4.0	29.0	0.0
L	R	14	1.0	42.0	18.0	4.0	34.0	1.0
L	R	15	0.0	39.0	14.0	5.0	40.0	1.0
L	R	16	1.0	37.0	18.0	2.0	42.0	0.0
L	R	17	1.0	41.0	9.0	5.0	44.0	0.0
L	R	18	0.0	42.0	15.0	1.0	41.0	1.0
L	R	19	0.0	38.0	13.0	10.0	39.0	0.0
L	R	20	0.0	44.0	12.0	2.0	41.0	0.0
L	R	21	0.0	45.0	16.0	4.0	34.0	1.0
L	R	22	0.0	41.0	28.0	1.0	30.0	0.0
L	R	23	2.0	39.0	22.0	0.0	37.0	0.0
L	R	24	0.0	44.0	21.0	2.0	34.0	0.0
L	R	25	0.0	33.0	23.0	3.0	40.0	0.0
L	R	26	1.0	36.0	29.0	0.0	32.0	2.0
L	R	27	0.0	37.0	19.0	2.0	41.0	1.0
L	R	28	1.0	31.0	19.0	2.0	45.0	1.0
L	R	29	0.0	36.0	22.0	2.0	39.0	1.0
L	R	30	0.0	37.0	30.0	0.0	33.0	0.0
L	FR	1	0.0	71.0	17.0	1.0	6.0	5.0
L	FR	2	0.0	79.0	11.0	2.0	5.0	4.0
L	FR	3	0.0	71.0	7.0	6.0	6.0	11.0
L	FR	4	1.0	68.0	10.0	4.0	6.0	12.0
L	FR	5	0.0	67.0	12.0	1.0	6.0	14.0
L	FR	6	1.0	66.0	12.0	1.0	9.0	9.0
L	FR	7	0.0	57.0	6.0	6.0	15.0	16.0
L	FR	8	1.0	54.0	7.0	5.0	8.0	24.0
L	FR	9	0.0	52.0	12.0	1.0	11.0	24.0
L	FR	10	0.0	57.0	8.0	3.0	9.0	23.0
L	FR	11	0.0	51.0	17.0	2.0	6.0	24.0
L	FR	12	0.0	59.0	12.0	2.0	7.0	20.0
L	FR	13	0.0	48.0	14.0	3.0	8.0	28.0

L	FR	14	1.0	49.0	21.0	4.0	7.0	17.0
L	FR	15	0.0	59.0	9.0	5.0	3.0	24.0
L	FR	16	0.0	55.0	13.0	5.0	13.0	15.0
L	FR	17	2.0	38.0	18.0	5.0	13.0	25.0
L	FR	18	0.0	50.0	13.0	7.0	7.0	23.0
L	FR	19	0.0	46.0	9.0	6.0	9.0	30.0
L	FR	20	1.0	51.0	9.0	5.0	13.0	20.0
L	FR	21	2.0	56.0	11.0	2.0	6.0	24.0
L	FR	22	0.0	57.0	7.0	3.0	7.0	27.0
L	FR	23	0.0	47.0	14.0	2.0	9.0	29.0
L	FR	24	0.0	51.0	13.0	0.0	8.0	29.0
L	FR	25	4.0	40.0	19.0	1.0	16.0	20.0
L	FR	26	2.0	44.0	16.0	0.0	7.0	32.0
L	FR	27	0.0	49.0	9.0	4.0	9.0	30.0
L	FR	28	0.0	44.0	9.0	2.0	8.0	38.0
L	FR	29	4.0	45.0	9.0	11.0	9.0	21.0
L	FR	30	0.0	41.0	19.0	5.0	14.0	21.0

Table S 11: Proportion of labelled videos falling under each political classification as a Left user begins to watch videos of a new political classification in the second stage of the experiment. Percentages highlighted in bold denote the video at which the proportion of videos labelled under the new class exceeded that of the original class.

**Original Class**	**New Class**	**Video Number**	**Far Left**	**Left**	**Center**	**Anti-Woke**	**Right**	**Far Right**
C	FL	1	11.0	15.0	68.0	2.0	4.0	0.0
C	FL	2	18.0	20.0	50.0	6.0	7.0	0.0
C	FL	3	24.0	18.0	45.0	8.0	6.0	0.0
C	FL	4	28.0	15.0	45.0	7.0	6.0	0.0
C	FL	5	33.0	16.0	44.0	2.0	5.0	0.0
C	FL	6	33.0	17.0	41.0	6.0	3.0	0.0
C	FL	7	34.0	18.0	38.0	4.0	6.0	0.0
C	FL	8	36.0	17.0	39.0	5.0	4.0	0.0
C	FL	9	39.0	15.0	34.0	10.0	1.0	1.0
C	FL	10	50.0	9.0	32.0	7.0	3.0	0.0
C	FL	11	39.0	17.0	33.0	7.0	3.0	0.0
C	FL	12	35.0	18.0	36.0	5.0	6.0	0.0
C	FL	13	**42.0**	12.0	34.0	8.0	4.0	0.0
C	FL	14	38.0	16.0	38.0	6.0	2.0	0.0
C	FL	15	46.0	14.0	35.0	4.0	1.0	0.0

C	FL	16	44.0	16.0	33.0	1.0	6.0	0.0
C	FL	17	44.0	17.0	33.0	3.0	3.0	0.0
C	FL	18	49.0	15.0	29.0	5.0	1.0	0.0
C	FL	19	50.0	11.0	35.0	1.0	3.0	0.0
C	FL	20	44.0	24.0	26.0	4.0	1.0	1.0
C	FL	21	48.0	15.0	36.0	1.0	0.0	0.0
C	FL	22	46.0	19.0	29.0	1.0	5.0	1.0
C	FL	23	43.0	17.0	33.0	4.0	4.0	0.0
C	FL	24	45.0	17.0	32.0	2.0	3.0	1.0
C	FL	25	54.0	11.0	29.0	4.0	3.0	0.0
C	FL	26	42.0	22.0	31.0	5.0	0.0	0.0
C	FL	27	46.0	14.0	29.0	9.0	2.0	0.0
C	FL	28	53.0	9.0	34.0	3.0	1.0	0.0
C	FL	29	52.0	14.0	29.0	2.0	3.0	0.0
C	FL	30	46.0	14.0	31.0	6.0	2.0	0.0
C	L	1	0.0	30.0	63.0	3.0	4.0	0.0
C	L	2	1.0	36.0	58.0	2.0	4.0	0.0
C	L	3	0.0	42.0	51.0	6.0	1.0	0.0
C	L	4	0.0	46.0	49.0	3.0	1.0	0.0
C	L	5	0.0	45.0	49.0	3.0	3.0	0.0
C	L	6	0.0	55.0	41.0	3.0	1.0	0.0
C	L	7	0.0	56.0	40.0	2.0	2.0	0.0
C	L	8	0.0	53.0	44.0	3.0	0.0	0.0
C	L	9	0.0	59.0	38.0	1.0	2.0	0.0
C	L	10	0.0	66.0	31.0	2.0	1.0	0.0
C	L	11	0.0	64.0	32.0	2.0	2.0	0.0
C	L	12	0.0	70.0	28.0	2.0	0.0	0.0
C	L	13	0.0	67.0	31.0	1.0	1.0	0.0
C	L	14	0.0	65.0	30.0	4.0	1.0	0.0
C	L	15	0.0	62.0	34.0	2.0	2.0	0.0
C	L	16	0.0	64.0	36.0	0.0	0.0	0.0
C	L	17	1.0	59.0	37.0	2.0	1.0	0.0
C	L	18	1.0	64.0	34.0	0.0	1.0	0.0
C	L	19	0.0	64.0	33.0	1.0	2.0	0.0
C	L	20	0.0	54.0	39.0	1.0	5.0	0.0
C	L	21	0.0	63.0	35.0	1.0	2.0	0.0
C	L	22	0.0	61.0	36.0	1.0	2.0	0.0
C	L	23	0.0	65.0	33.0	1.0	1.0	0.0
C	L	24	0.0	48.0	50.0	1.0	1.0	0.0
C	L	25	0.0	58.0	37.0	2.0	3.0	0.0

C	L	26	0.0	**53.0**	45.0	0.0	2.0	0.0
C	L	27	0.0	65.0	34.0	0.0	1.0	0.0
C	L	28	0.0	57.0	40.0	1.0	2.0	0.0
C	L	29	1.0	58.0	39.0	1.0	1.0	0.0
C	L	30	0.0	55.0	42.0	1.0	2.0	0.0
C	C	1	0.0	17.0	77.0	5.0	2.0	0.0
C	C	2	0.0	18.0	70.0	4.0	9.0	0.0
C	C	3	0.0	19.0	68.0	7.0	7.0	0.0
C	C	4	0.0	16.0	75.0	4.0	4.0	0.0
C	C	5	1.0	15.0	75.0	2.0	7.0	0.0
C	C	6	0.0	23.0	64.0	7.0	6.0	0.0
C	C	7	0.0	24.0	73.0	0.0	3.0	0.0
C	C	8	0.0	15.0	72.0	8.0	4.0	1.0
C	C	9	0.0	20.0	71.0	2.0	8.0	0.0
C	C	10	0.0	15.0	73.0	5.0	6.0	0.0
C	C	11	0.0	20.0	75.0	4.0	1.0	0.0
C	C	12	0.0	21.0	73.0	3.0	3.0	0.0
C	C	13	0.0	16.0	77.0	5.0	2.0	1.0
C	C	14	0.0	18.0	73.0	4.0	4.0	0.0
C	C	15	0.0	17.0	78.0	3.0	2.0	0.0
C	C	16	0.0	16.0	80.0	3.0	1.0	0.0
C	C	17	0.0	16.0	78.0	3.0	3.0	0.0
C	C	18	0.0	16.0	80.0	3.0	1.0	0.0
C	C	19	0.0	24.0	72.0	4.0	1.0	0.0
C	C	20	0.0	17.0	75.0	6.0	2.0	0.0
C	C	21	0.0	15.0	84.0	1.0	0.0	0.0
C	C	22	0.0	14.0	86.0	0.0	0.0	0.0
C	C	23	0.0	18.0	78.0	4.0	1.0	0.0
C	C	24	0.0	17.0	78.0	4.0	2.0	0.0
C	C	25	0.0	13.0	81.0	2.0	3.0	0.0
C	C	26	0.0	16.0	76.0	2.0	6.0	0.0
C	C	27	1.0	13.0	79.0	5.0	2.0	0.0
C	C	28	0.0	11.0	82.0	3.0	4.0	0.0
C	C	29	0.0	16.0	81.0	3.0	1.0	0.0
C	C	30	0.0	12.0	87.0	1.0	1.0	0.0
C	AW	1	1.0	11.0	63.0	19.0	7.0	0.0
C	AW	2	0.0	18.0	45.0	33.0	5.0	0.0
C	AW	3	1.0	18.0	43.0	35.0	3.0	0.0
C	AW	4	2.0	13.0	35.0	44.0	6.0	0.0
C	AW	5	0.0	15.0	38.0	38.0	7.0	1.0

C	AW	6	0.0	18.0	43.0	37.0	2.0	0.0
C	AW	7	0.0	15.0	41.0	38.0	5.0	0.0
C	AW	8	1.0	9.0	36.0	48.0	6.0	0.0
C	AW	9	0.0	10.0	37.0	49.0	3.0	0.0
C	AW	10	1.0	10.0	41.0	41.0	7.0	0.0
C	AW	11	0.0	12.0	41.0	39.0	8.0	0.0
C	AW	12	0.0	7.0	30.0	59.0	4.0	0.0
C	AW	13	0.0	11.0	41.0	40.0	5.0	2.0
C	AW	14	0.0	6.0	44.0	**46.0**	5.0	0.0
C	AW	15	0.0	16.0	38.0	39.0	5.0	1.0
C	AW	16	0.0	9.0	32.0	53.0	6.0	0.0
C	AW	17	0.0	14.0	31.0	43.0	11.0	1.0
C	AW	18	1.0	9.0	38.0	43.0	9.0	0.0
C	AW	19	1.0	11.0	31.0	48.0	9.0	0.0
C	AW	20	0.0	4.0	39.0	52.0	4.0	0.0
C	AW	21	1.0	16.0	30.0	43.0	10.0	0.0
C	AW	22	1.0	12.0	29.0	49.0	9.0	0.0
C	AW	23	0.0	8.0	35.0	45.0	13.0	0.0
C	AW	24	1.0	3.0	37.0	54.0	5.0	0.0
C	AW	25	0.0	5.0	40.0	50.0	4.0	0.0
C	AW	26	1.0	12.0	24.0	53.0	8.0	1.0
C	AW	27	0.0	13.0	35.0	42.0	10.0	0.0
C	AW	28	0.0	9.0	30.0	54.0	6.0	1.0
C	AW	29	1.0	11.0	29.0	48.0	11.0	0.0
C	AW	30	0.0	13.0	28.0	51.0	8.0	0.0
C	R	1	0.0	15.0	63.0	4.0	18.0	1.0
C	R	2	0.0	18.0	66.0	1.0	12.0	3.0
C	R	3	0.0	16.0	54.0	5.0	23.0	3.0
C	R	4	0.0	13.0	58.0	6.0	22.0	1.0
C	R	5	0.0	16.0	51.0	2.0	29.0	2.0
C	R	6	0.0	17.0	49.0	5.0	27.0	2.0
C	R	7	0.0	12.0	47.0	7.0	33.0	0.0
C	R	8	1.0	12.0	47.0	4.0	36.0	0.0
C	R	9	1.0	14.0	43.0	6.0	36.0	0.0
C	R	10	0.0	14.0	41.0	5.0	38.0	2.0
C	R	11	0.0	11.0	48.0	6.0	35.0	0.0
C	R	12	0.0	17.0	34.0	6.0	41.0	2.0
C	R	13	0.0	11.0	46.0	4.0	39.0	0.0
C	R	14	0.0	9.0	45.0	6.0	40.0	1.0
C	R	15	2.0	14.0	43.0	3.0	37.0	1.0

C	R	16	0.0	17.0	49.0	3.0	32.0	0.0
C	R	17	0.0	12.0	51.0	3.0	32.0	2.0
C	R	18	0.0	15.0	34.0	6.0	44.0	1.0
C	R	19	0.0	13.0	43.0	4.0	39.0	0.0
C	R	20	0.0	10.0	38.0	8.0	42.0	2.0
C	R	21	0.0	19.0	44.0	0.0	36.0	1.0
C	R	22	0.0	11.0	41.0	3.0	45.0	0.0
C	R	23	1.0	17.0	40.0	2.0	40.0	0.0
C	R	24	0.0	18.0	36.0	0.0	45.0	1.0
C	R	25	0.0	15.0	42.0	2.0	41.0	0.0
C	R	26	1.0	15.0	46.0	3.0	36.0	0.0
C	R	27	0.0	14.0	42.0	4.0	39.0	1.0
C	R	28	0.0	16.0	39.0	2.0	43.0	0.0
C	R	29	0.0	18.0	32.0	7.0	43.0	0.0
C	R	30	0.0	13.0	48.0	3.0	36.0	0.0
C	FR	1	0.0	14.0	68.0	5.0	10.0	3.0
C	FR	2	1.0	15.0	67.0	6.0	4.0	6.0
C	FR	3	0.0	15.0	62.0	0.0	14.0	8.0
C	FR	4	1.0	11.0	63.0	4.0	9.0	12.0
C	FR	5	0.0	23.0	46.0	6.0	12.0	12.0
C	FR	6	0.0	12.0	51.0	7.0	15.0	15.0
C	FR	7	0.0	12.0	51.0	7.0	7.0	24.0
C	FR	8	0.0	7.0	57.0	6.0	14.0	16.0
C	FR	9	0.0	14.0	50.0	8.0	7.0	22.0
C	FR	10	1.0	12.0	42.0	5.0	20.0	20.0
C	FR	11	2.0	11.0	47.0	6.0	9.0	26.0
C	FR	12	0.0	14.0	53.0	2.0	17.0	14.0
C	FR	13	0.0	23.0	41.0	1.0	9.0	25.0
C	FR	14	1.0	7.0	52.0	11.0	11.0	18.0
C	FR	15	0.0	23.0	45.0	4.0	7.0	20.0
C	FR	16	2.0	14.0	51.0	3.0	7.0	24.0
C	FR	17	1.0	14.0	42.0	5.0	12.0	25.0
C	FR	18	2.0	13.0	43.0	8.0	12.0	22.0
C	FR	19	0.0	17.0	51.0	0.0	8.0	24.0
C	FR	20	0.0	10.0	47.0	8.0	8.0	27.0
C	FR	21	0.0	7.0	54.0	4.0	13.0	22.0
C	FR	22	0.0	14.0	48.0	2.0	17.0	20.0
C	FR	23	0.0	15.0	46.0	3.0	7.0	28.0
C	FR	24	0.0	16.0	51.0	1.0	10.0	22.0
C	FR	25	1.0	15.0	47.0	7.0	4.0	25.0

C	FR	26	0.0	13.0	52.0	0.0	8.0	28.0
C	FR	27	1.0	16.0	43.0	1.0	16.0	22.0
C	FR	28	0.0	11.0	61.0	0.0	8.0	20.0
C	FR	29	0.0	18.0	44.0	1.0	12.0	25.0
C	FR	30	0.0	15.0	53.0	4.0	13.0	15.0

Table S 12: Proportion of labelled videos falling under each political classification as a Center user begins to watch videos of a new political classification in the second stage of the experiment. Percentages highlighted in bold denote the video at which the proportion of videos labelled under the new class exceeded that of the original class.

**Original Class**	**New Class**	**Video Number**	**Far Left**	**Left**	**Center**	**Anti-Woke**	**Right**	**Far Right**
AW	FL	1	25.0	4.0	4.0	61.0	6.0	0.0
AW	FL	2	25.0	8.0	2.0	58.0	8.0	0.0
AW	FL	3	31.0	6.0	3.0	52.0	6.0	2.0
AW	FL	4	32.0	11.0	3.0	51.0	3.0	0.0
AW	FL	5	36.0	8.0	3.0	45.0	8.0	0.0
AW	FL	6	37.0	10.0	1.0	46.0	4.0	1.0
AW	FL	7	**47.0**	2.0	1.0	43.0	7.0	0.0
AW	FL	8	47.0	9.0	1.0	39.0	4.0	1.0
AW	FL	9	50.0	10.0	1.0	35.0	4.0	0.0
AW	FL	10	44.0	12.0	0.0	39.0	4.0	0.0
AW	FL	11	44.0	8.0	2.0	40.0	4.0	2.0
AW	FL	12	43.0	10.0	2.0	42.0	3.0	0.0
AW	FL	13	46.0	13.0	5.0	31.0	5.0	0.0
AW	FL	14	51.0	10.0	2.0	35.0	0.0	1.0
AW	FL	15	48.0	9.0	2.0	36.0	5.0	0.0
AW	FL	16	49.0	9.0	4.0	37.0	2.0	0.0
AW	FL	17	52.0	13.0	2.0	31.0	2.0	0.0
AW	FL	18	56.0	13.0	5.0	24.0	2.0	0.0
AW	FL	19	60.0	5.0	1.0	29.0	3.0	1.0
AW	FL	20	58.0	6.0	2.0	33.0	1.0	0.0
AW	FL	21	55.0	12.0	2.0	31.0	0.0	0.0
AW	FL	22	59.0	9.0	3.0	29.0	1.0	0.0
AW	FL	23	52.0	6.0	7.0	32.0	3.0	0.0
AW	FL	24	53.0	8.0	9.0	27.0	4.0	0.0
AW	FL	25	55.0	8.0	4.0	30.0	3.0	0.0
AW	FL	26	49.0	10.0	7.0	31.0	3.0	0.0
AW	FL	27	52.0	13.0	7.0	27.0	1.0	0.0

AW	FL	28	53.0	17.0	4.0	25.0	1.0	0.0
AW	FL	29	52.0	13.0	7.0	25.0	3.0	0.0
AW	FL	30	56.0	10.0	3.0	27.0	3.0	1.0
AW	L	1	2.0	29.0	8.0	56.0	5.0	0.0
AW	L	2	0.0	**47.0**	5.0	44.0	5.0	0.0
AW	L	3	0.0	46.0	7.0	44.0	2.0	0.0
AW	L	4	1.0	47.0	10.0	37.0	3.0	1.0
AW	L	5	0.0	58.0	7.0	29.0	7.0	0.0
AW	L	6	0.0	56.0	8.0	34.0	2.0	0.0
AW	L	7	0.0	60.0	6.0	27.0	7.0	0.0
AW	L	8	0.0	62.0	5.0	30.0	3.0	0.0
AW	L	9	0.0	62.0	7.0	28.0	3.0	0.0
AW	L	10	0.0	70.0	7.0	22.0	1.0	0.0
AW	L	11	1.0	57.0	13.0	27.0	1.0	0.0
AW	L	12	1.0	63.0	7.0	26.0	2.0	0.0
AW	L	13	0.0	65.0	9.0	24.0	3.0	0.0
AW	L	14	2.0	56.0	15.0	20.0	7.0	0.0
AW	L	15	0.0	66.0	7.0	23.0	4.0	0.0
AW	L	16	1.0	60.0	10.0	25.0	4.0	0.0
AW	L	17	0.0	49.0	14.0	29.0	7.0	0.0
AW	L	18	0.0	72.0	7.0	18.0	3.0	0.0
AW	L	19	0.0	66.0	12.0	18.0	3.0	1.0
AW	L	20	1.0	65.0	10.0	24.0	0.0	0.0
AW	L	21	0.0	60.0	14.0	26.0	0.0	0.0
AW	L	22	0.0	65.0	18.0	17.0	1.0	0.0
AW	L	23	0.0	70.0	8.0	21.0	1.0	0.0
AW	L	24	0.0	63.0	13.0	20.0	3.0	0.0
AW	L	25	0.0	66.0	13.0	18.0	2.0	0.0
AW	L	26	0.0	68.0	17.0	14.0	0.0	0.0
AW	L	27	0.0	63.0	19.0	18.0	0.0	0.0
AW	L	28	0.0	61.0	15.0	20.0	4.0	0.0
AW	L	29	0.0	67.0	15.0	17.0	0.0	0.0
AW	L	30	0.0	64.0	16.0	18.0	1.0	1.0
AW	C	1	1.0	14.0	25.0	52.0	9.0	0.0
AW	C	2	1.0	6.0	32.0	51.0	8.0	1.0
AW	C	3	1.0	11.0	37.0	46.0	4.0	0.0
AW	C	4	1.0	5.0	38.0	46.0	9.0	0.0
AW	C	5	1.0	9.0	39.0	43.0	8.0	0.0
AW	C	6	1.0	7.0	**54.0**	32.0	6.0	0.0
AW	C	7	0.0	8.0	46.0	38.0	8.0	0.0

AW	C	8	2.0	10.0	48.0	36.0	2.0	1.0
AW	C	9	1.0	10.0	52.0	30.0	6.0	0.0
AW	C	10	0.0	13.0	41.0	41.0	6.0	0.0
AW	C	11	0.0	10.0	55.0	30.0	3.0	1.0
AW	C	12	0.0	14.0	54.0	27.0	4.0	0.0
AW	C	13	0.0	12.0	58.0	20.0	10.0	0.0
AW	C	14	0.0	19.0	60.0	15.0	6.0	0.0
AW	C	15	0.0	13.0	52.0	27.0	8.0	0.0
AW	C	16	3.0	12.0	59.0	21.0	5.0	0.0
AW	C	17	0.0	17.0	49.0	28.0	5.0	0.0
AW	C	18	0.0	26.0	47.0	23.0	5.0	0.0
AW	C	19	1.0	17.0	61.0	19.0	2.0	0.0
AW	C	20	0.0	12.0	67.0	19.0	2.0	0.0
AW	C	21	0.0	14.0	61.0	23.0	2.0	0.0
AW	C	22	0.0	12.0	63.0	20.0	4.0	0.0
AW	C	23	0.0	8.0	63.0	22.0	6.0	1.0
AW	C	24	0.0	17.0	65.0	14.0	4.0	0.0
AW	C	25	0.0	17.0	62.0	16.0	6.0	0.0
AW	C	26	1.0	9.0	64.0	23.0	2.0	0.0
AW	C	27	0.0	8.0	72.0	13.0	5.0	1.0
AW	C	28	1.0	10.0	60.0	22.0	7.0	0.0
AW	C	29	0.0	11.0	66.0	18.0	5.0	0.0
AW	C	30	0.0	18.0	61.0	19.0	3.0	0.0
AW	AW	1	0.0	4.0	11.0	81.0	5.0	0.0
AW	AW	2	0.0	9.0	3.0	75.0	13.0	0.0
AW	AW	3	0.0	7.0	5.0	83.0	5.0	0.0
AW	AW	4	0.0	5.0	5.0	87.0	3.0	0.0
AW	AW	5	0.0	4.0	0.0	88.0	8.0	0.0
AW	AW	6	1.0	4.0	1.0	85.0	9.0	0.0
AW	AW	7	1.0	3.0	4.0	80.0	9.0	2.0
AW	AW	8	0.0	5.0	6.0	83.0	6.0	0.0
AW	AW	9	0.0	3.0	5.0	80.0	12.0	0.0
AW	AW	10	0.0	1.0	8.0	80.0	11.0	0.0
AW	AW	11	0.0	2.0	2.0	91.0	5.0	0.0
AW	AW	12	0.0	5.0	5.0	83.0	6.0	0.0
AW	AW	13	0.0	4.0	3.0	84.0	10.0	0.0
AW	AW	14	1.0	3.0	2.0	89.0	4.0	0.0
AW	AW	15	1.0	4.0	0.0	88.0	7.0	0.0
AW	AW	16	0.0	4.0	2.0	85.0	7.0	1.0
AW	AW	17	1.0	1.0	4.0	85.0	9.0	0.0

AW	AW	18	0.0	2.0	8.0	80.0	9.0	1.0
AW	AW	19	0.0	6.0	6.0	82.0	5.0	0.0
AW	AW	20	0.0	2.0	3.0	89.0	6.0	0.0
AW	AW	21	0.0	4.0	2.0	87.0	7.0	0.0
AW	AW	22	1.0	4.0	2.0	88.0	4.0	0.0
AW	AW	23	0.0	2.0	4.0	87.0	7.0	0.0
AW	AW	24	0.0	4.0	5.0	84.0	7.0	0.0
AW	AW	25	0.0	2.0	2.0	88.0	7.0	0.0
AW	AW	26	0.0	2.0	5.0	88.0	5.0	0.0
AW	AW	27	0.0	4.0	2.0	87.0	6.0	0.0
AW	AW	28	1.0	9.0	4.0	77.0	8.0	0.0
AW	AW	29	1.0	2.0	3.0	84.0	10.0	0.0
AW	AW	30	0.0	5.0	8.0	78.0	8.0	0.0
AW	R	1	0.0	9.0	6.0	63.0	21.0	1.0
AW	R	2	0.0	2.0	4.0	68.0	23.0	2.0
AW	R	3	0.0	5.0	6.0	54.0	34.0	1.0
AW	R	4	0.0	5.0	6.0	52.0	35.0	2.0
AW	R	5	1.0	7.0	4.0	47.0	40.0	1.0
AW	R	6	0.0	6.0	7.0	48.0	39.0	1.0
AW	R	7	0.0	6.0	9.0	41.0	45.0	0.0
AW	R	8	1.0	2.0	14.0	42.0	39.0	2.0
AW	R	9	1.0	3.0	9.0	52.0	34.0	0.0
AW	R	10	1.0	4.0	8.0	40.0	45.0	1.0
AW	R	11	0.0	4.0	6.0	45.0	42.0	2.0
AW	R	12	0.0	11.0	10.0	35.0	42.0	1.0
AW	R	13	0.0	8.0	11.0	37.0	43.0	0.0
AW	R	14	0.0	10.0	13.0	31.0	44.0	2.0
AW	R	15	1.0	14.0	8.0	45.0	30.0	1.0
AW	R	16	0.0	6.0	7.0	38.0	49.0	0.0
AW	R	17	1.0	10.0	16.0	27.0	44.0	1.0
AW	R	18	0.0	11.0	15.0	31.0	41.0	3.0
AW	R	19	0.0	7.0	14.0	30.0	47.0	2.0
AW	R	20	0.0	8.0	10.0	33.0	45.0	3.0
AW	R	21	0.0	9.0	24.0	31.0	35.0	1.0
AW	R	22	1.0	4.0	22.0	33.0	40.0	0.0
AW	R	23	0.0	11.0	18.0	22.0	49.0	0.0
AW	R	24	2.0	5.0	26.0	21.0	44.0	2.0
AW	R	25	0.0	12.0	21.0	26.0	38.0	3.0
AW	R	26	2.0	14.0	23.0	33.0	28.0	0.0
AW	R	27	0.0	9.0	21.0	23.0	**45.0**	1.0

AW	R	28	1.0	14.0	19.0	33.0	33.0	0.0
AW	R	29	0.0	7.0	21.0	28.0	42.0	2.0
AW	R	30	0.0	12.0	29.0	20.0	39.0	0.0
AW	FR	1	0.0	1.0	5.0	79.0	12.0	3.0
AW	FR	2	0.0	4.0	2.0	75.0	12.0	7.0
AW	FR	3	0.0	1.0	1.0	71.0	14.0	12.0
AW	FR	4	0.0	1.0	3.0	71.0	8.0	16.0
AW	FR	5	1.0	4.0	5.0	55.0	11.0	24.0
AW	FR	6	4.0	0.0	1.0	63.0	12.0	20.0
AW	FR	7	0.0	1.0	4.0	61.0	16.0	17.0
AW	FR	8	1.0	3.0	1.0	64.0	9.0	21.0
AW	FR	9	0.0	1.0	1.0	59.0	16.0	22.0
AW	FR	10	0.0	3.0	1.0	51.0	22.0	23.0
AW	FR	11	0.0	4.0	3.0	57.0	17.0	20.0
AW	FR	12	0.0	3.0	3.0	67.0	15.0	12.0
AW	FR	13	3.0	4.0	1.0	49.0	16.0	27.0
AW	FR	14	3.0	0.0	4.0	59.0	13.0	22.0
AW	FR	15	2.0	0.0	7.0	49.0	19.0	23.0
AW	FR	16	0.0	2.0	1.0	54.0	19.0	24.0
AW	FR	17	1.0	5.0	5.0	57.0	18.0	14.0
AW	FR	18	0.0	8.0	3.0	44.0	21.0	25.0
AW	FR	19	0.0	1.0	2.0	57.0	10.0	30.0
AW	FR	20	0.0	3.0	1.0	56.0	21.0	19.0
AW	FR	21	1.0	6.0	10.0	51.0	7.0	25.0
AW	FR	22	0.0	8.0	8.0	48.0	8.0	28.0
AW	FR	23	0.0	2.0	2.0	50.0	18.0	27.0
AW	FR	24	0.0	6.0	1.0	44.0	18.0	31.0
AW	FR	25	3.0	1.0	4.0	52.0	16.0	24.0
AW	FR	26	0.0	3.0	1.0	62.0	14.0	20.0
AW	FR	27	0.0	6.0	2.0	62.0	11.0	19.0
AW	FR	28	0.0	1.0	2.0	50.0	12.0	34.0
AW	FR	29	0.0	5.0	8.0	50.0	12.0	26.0
AW	FR	30	1.0	2.0	5.0	48.0	16.0	27.0

Table S 13: Proportion of labelled videos falling under each political classification as a Anti-Woke user begins to watch videos of a new political classification in the second stage of the experiment. Percentages highlighted in bold denote the video at which the proportion of videos labelled under the new class exceeded that of the original class.

**Original**	**New**	**Video**	**Far**	**Left**	**Center**	**Anti-**	**Right**	**Far**

Class	Class	Number	Left			Woke		Right
R	FL	1	23.0	8.0	14.0	12.0	42.0	1.0
R	FL	2	29.0	9.0	10.0	13.0	37.0	2.0
R	FL	3	33.0	9.0	13.0	9.0	35.0	1.0
R	FL	4	37.0	8.0	12.0	2.0	40.0	1.0
R	FL	5	34.0	12.0	16.0	5.0	31.0	1.0
R	FL	6	33.0	12.0	5.0	11.0	38.0	1.0
R	FL	7	**38.0**	13.0	5.0	7.0	33.0	4.0
R	FL	8	35.0	15.0	5.0	10.0	32.0	3.0
R	FL	9	41.0	10.0	8.0	7.0	32.0	1.0
R	FL	10	59.0	7.0	4.0	10.0	20.0	0.0
R	FL	11	41.0	14.0	11.0	10.0	23.0	1.0
R	FL	12	35.0	15.0	12.0	6.0	33.0	0.0
R	FL	13	43.0	16.0	3.0	9.0	28.0	1.0
R	FL	14	39.0	15.0	11.0	9.0	25.0	1.0
R	FL	15	48.0	15.0	6.0	12.0	18.0	0.0
R	FL	16	52.0	18.0	1.0	6.0	22.0	0.0
R	FL	17	57.0	13.0	7.0	8.0	16.0	0.0
R	FL	18	43.0	15.0	7.0	8.0	26.0	0.0
R	FL	19	44.0	13.0	6.0	10.0	27.0	0.0
R	FL	20	51.0	12.0	9.0	9.0	19.0	0.0
R	FL	21	47.0	20.0	5.0	6.0	21.0	1.0
R	FL	22	58.0	7.0	10.0	8.0	17.0	0.0
R	FL	23	56.0	12.0	16.0	6.0	10.0	0.0
R	FL	24	62.0	13.0	11.0	3.0	12.0	0.0
R	FL	25	59.0	12.0	7.0	6.0	15.0	0.0
R	FL	26	57.0	4.0	10.0	9.0	18.0	1.0
R	FL	27	48.0	14.0	11.0	9.0	17.0	1.0
R	FL	28	47.0	9.0	16.0	9.0	18.0	0.0
R	FL	29	53.0	15.0	12.0	6.0	14.0	0.0
R	FL	30	56.0	17.0	7.0	4.0	17.0	0.0
R	L	1	0.0	34.0	22.0	2.0	42.0	0.0
R	L	2	1.0	**43.0**	16.0	9.0	29.0	2.0
R	L	3	4.0	43.0	14.0	5.0	32.0	1.0
R	L	4	0.0	57.0	16.0	1.0	24.0	2.0
R	L	5	0.0	56.0	17.0	3.0	22.0	1.0
R	L	6	0.0	49.0	21.0	2.0	28.0	0.0
R	L	7	1.0	56.0	17.0	3.0	23.0	0.0
R	L	8	2.0	55.0	11.0	3.0	29.0	1.0
R	L	9	1.0	57.0	19.0	1.0	22.0	0.0

R	L	10	2.0	62.0	11.0	2.0	23.0	0.0
R	L	11	0.0	61.0	12.0	1.0	25.0	1.0
R	L	12	0.0	61.0	19.0	4.0	16.0	0.0
R	L	13	1.0	63.0	21.0	1.0	14.0	0.0
R	L	14	0.0	59.0	24.0	3.0	14.0	0.0
R	L	15	0.0	62.0	17.0	2.0	19.0	0.0
R	L	16	0.0	57.0	24.0	2.0	15.0	1.0
R	L	17	0.0	63.0	17.0	0.0	20.0	0.0
R	L	18	0.0	51.0	25.0	2.0	21.0	1.0
R	L	19	0.0	65.0	20.0	1.0	14.0	0.0
R	L	20	0.0	58.0	19.0	1.0	22.0	0.0
R	L	21	0.0	64.0	20.0	0.0	16.0	0.0
R	L	22	0.0	70.0	19.0	0.0	11.0	0.0
R	L	23	0.0	59.0	23.0	2.0	17.0	0.0
R	L	24	3.0	60.0	21.0	3.0	12.0	1.0
R	L	25	0.0	55.0	29.0	1.0	15.0	0.0
R	L	26	1.0	65.0	14.0	0.0	20.0	0.0
R	L	27	0.0	60.0	25.0	0.0	14.0	0.0
R	L	28	0.0	58.0	19.0	2.0	20.0	1.0
R	L	29	0.0	69.0	20.0	0.0	12.0	0.0
R	L	30	0.0	60.0	20.0	1.0	18.0	0.0
R	C	1	0.0	15.0	**42.0**	3.0	39.0	1.0
R	C	2	0.0	11.0	45.0	8.0	35.0	1.0
R	C	3	0.0	11.0	51.0	7.0	31.0	0.0
R	C	4	1.0	9.0	56.0	14.0	20.0	0.0
R	C	5	0.0	17.0	51.0	10.0	22.0	0.0
R	C	6	0.0	11.0	55.0	12.0	20.0	1.0
R	C	7	0.0	11.0	63.0	9.0	17.0	0.0
R	C	8	0.0	17.0	47.0	7.0	29.0	0.0
R	C	9	1.0	15.0	57.0	7.0	18.0	1.0
R	C	10	0.0	24.0	49.0	8.0	18.0	1.0
R	C	11	1.0	15.0	58.0	5.0	18.0	3.0
R	C	12	1.0	19.0	61.0	9.0	9.0	0.0
R	C	13	0.0	11.0	61.0	6.0	22.0	0.0
R	C	14	0.0	21.0	67.0	2.0	9.0	1.0
R	C	15	1.0	24.0	66.0	3.0	6.0	0.0
R	C	16	0.0	22.0	58.0	5.0	16.0	0.0
R	C	17	1.0	21.0	62.0	6.0	9.0	1.0
R	C	18	0.0	18.0	61.0	4.0	17.0	0.0
R	C	19	0.0	18.0	56.0	6.0	20.0	0.0

R	C	20	0.0	13.0	74.0	1.0	12.0	0.0
R	C	21	0.0	20.0	61.0	1.0	18.0	0.0
R	C	22	0.0	13.0	69.0	3.0	13.0	1.0
R	C	23	0.0	18.0	68.0	1.0	11.0	2.0
R	C	24	0.0	17.0	63.0	3.0	15.0	2.0
R	C	25	0.0	11.0	73.0	3.0	12.0	0.0
R	C	26	0.0	15.0	68.0	1.0	15.0	0.0
R	C	27	0.0	12.0	70.0	2.0	16.0	0.0
R	C	28	1.0	9.0	77.0	2.0	10.0	0.0
R	C	29	0.0	21.0	64.0	4.0	11.0	0.0
R	C	30	0.0	12.0	71.0	1.0	14.0	1.0
R	AW	1	0.0	4.0	11.0	38.0	46.0	1.0
R	AW	2	1.0	8.0	19.0	**37.0**	32.0	4.0
R	AW	3	1.0	6.0	10.0	43.0	37.0	2.0
R	AW	4	0.0	4.0	5.0	54.0	36.0	0.0
R	AW	5	0.0	8.0	15.0	41.0	36.0	0.0
R	AW	6	0.0	4.0	17.0	47.0	32.0	0.0
R	AW	7	0.0	5.0	11.0	53.0	30.0	1.0
R	AW	8	0.0	2.0	7.0	55.0	34.0	1.0
R	AW	9	0.0	5.0	9.0	51.0	32.0	3.0
R	AW	10	0.0	9.0	7.0	57.0	25.0	2.0
R	AW	11	0.0	5.0	11.0	58.0	26.0	0.0
R	AW	12	0.0	5.0	7.0	53.0	34.0	1.0
R	AW	13	0.0	5.0	7.0	51.0	37.0	0.0
R	AW	14	0.0	2.0	10.0	55.0	32.0	1.0
R	AW	15	0.0	10.0	8.0	58.0	23.0	1.0
R	AW	16	0.0	10.0	12.0	53.0	23.0	1.0
R	AW	17	0.0	8.0	8.0	56.0	29.0	0.0
R	AW	18	0.0	6.0	8.0	49.0	34.0	2.0
R	AW	19	0.0	9.0	7.0	59.0	25.0	0.0
R	AW	20	0.0	6.0	8.0	65.0	21.0	0.0
R	AW	21	0.0	6.0	9.0	57.0	26.0	1.0
R	AW	22	0.0	9.0	8.0	51.0	31.0	1.0
R	AW	23	0.0	4.0	11.0	57.0	29.0	0.0
R	AW	24	1.0	4.0	12.0	54.0	28.0	1.0
R	AW	25	1.0	6.0	8.0	57.0	26.0	3.0
R	AW	26	1.0	4.0	7.0	58.0	28.0	2.0
R	AW	27	1.0	6.0	6.0	58.0	29.0	0.0
R	AW	28	1.0	9.0	6.0	58.0	25.0	1.0
R	AW	29	0.0	7.0	6.0	61.0	24.0	2.0

R	AW	30	0.0	1.0	6.0	65.0	27.0	0.0
R	R	1	2.0	9.0	14.0	3.0	68.0	3.0
R	R	2	0.0	10.0	19.0	3.0	61.0	6.0
R	R	3	0.0	9.0	21.0	6.0	62.0	2.0
R	R	4	0.0	6.0	22.0	3.0	68.0	1.0
R	R	5	0.0	7.0	16.0	2.0	73.0	2.0
R	R	6	0.0	8.0	20.0	7.0	63.0	1.0
R	R	7	0.0	2.0	13.0	4.0	80.0	0.0
R	R	8	0.0	13.0	17.0	7.0	63.0	1.0
R	R	9	0.0	12.0	23.0	2.0	59.0	4.0
R	R	10	0.0	6.0	24.0	4.0	64.0	2.0
R	R	11	0.0	8.0	14.0	3.0	70.0	4.0
R	R	12	0.0	8.0	23.0	2.0	63.0	3.0
R	R	13	1.0	12.0	13.0	4.0	67.0	4.0
R	R	14	0.0	7.0	24.0	2.0	62.0	5.0
R	R	15	0.0	10.0	22.0	2.0	63.0	2.0
R	R	16	0.0	6.0	22.0	1.0	66.0	5.0
R	R	17	1.0	13.0	17.0	2.0	64.0	2.0
R	R	18	1.0	6.0	20.0	3.0	69.0	1.0
R	R	19	0.0	19.0	22.0	1.0	56.0	1.0
R	R	20	1.0	9.0	23.0	1.0	65.0	0.0
R	R	21	0.0	22.0	19.0	4.0	54.0	1.0
R	R	22	1.0	8.0	24.0	2.0	64.0	1.0
R	R	23	0.0	15.0	21.0	1.0	61.0	1.0
R	R	24	1.0	11.0	21.0	4.0	59.0	4.0
R	R	25	0.0	10.0	22.0	6.0	61.0	1.0
R	R	26	0.0	10.0	33.0	3.0	52.0	1.0
R	R	27	0.0	13.0	27.0	1.0	57.0	2.0
R	R	28	0.0	13.0	32.0	5.0	49.0	1.0
R	R	29	0.0	9.0	26.0	5.0	59.0	1.0
R	R	30	0.0	14.0	23.0	4.0	58.0	1.0
R	FR	1	0.0	17.0	13.0	4.0	58.0	8.0
R	FR	2	0.0	15.0	14.0	6.0	51.0	14.0
R	FR	3	0.0	10.0	13.0	9.0	47.0	21.0
R	FR	4	0.0	12.0	13.0	10.0	46.0	19.0
R	FR	5	1.0	11.0	17.0	6.0	43.0	23.0
R	FR	6	0.0	8.0	18.0	6.0	56.0	12.0
R	FR	7	0.0	5.0	19.0	1.0	59.0	16.0
R	FR	8	0.0	9.0	22.0	2.0	42.0	24.0
R	FR	9	1.0	8.0	12.0	6.0	51.0	22.0

R	FR	10	1.0	9.0	16.0	7.0	47.0	20.0
R	FR	11	1.0	12.0	12.0	1.0	57.0	16.0
R	FR	12	1.0	11.0	24.0	7.0	45.0	13.0
R	FR	13	3.0	12.0	15.0	7.0	43.0	21.0
R	FR	14	0.0	5.0	16.0	11.0	50.0	18.0
R	FR	15	0.0	6.0	23.0	3.0	42.0	26.0
R	FR	16	0.0	7.0	12.0	7.0	53.0	21.0
R	FR	17	1.0	3.0	19.0	7.0	46.0	24.0
R	FR	18	0.0	9.0	14.0	5.0	41.0	31.0
R	FR	19	0.0	8.0	20.0	10.0	35.0	27.0
R	FR	20	0.0	12.0	10.0	7.0	47.0	24.0
R	FR	21	0.0	9.0	19.0	8.0	38.0	26.0
R	FR	22	1.0	4.0	19.0	8.0	46.0	22.0
R	FR	23	2.0	12.0	18.0	5.0	37.0	27.0
R	FR	24	0.0	6.0	12.0	3.0	48.0	30.0
R	FR	25	1.0	5.0	10.0	4.0	40.0	41.0
R	FR	26	0.0	9.0	20.0	1.0	34.0	36.0
R	FR	27	0.0	8.0	15.0	4.0	42.0	30.0
R	FR	28	0.0	3.0	10.0	5.0	47.0	35.0
R	FR	29	0.0	6.0	15.0	7.0	49.0	24.0
R	FR	30	1.0	9.0	13.0	4.0	46.0	26.0

Table S 14: Proportion of labelled videos falling under each political classification as a Right user begins to watch videos of a new political classification in the second stage of the experiment. Percentages highlighted in bold denote the video at which the proportion of videos labelled under the new class exceeded that of the original class.

**Original Class**	**New Class**	**Video Number**	**Far Left**	**Left**	**Center**	**Anti-Woke**	**Right**	**Far Right**
FR	FL	1	27.0	8.0	6.0	10.0	21.0	27.0
FR	FL	2	26.0	4.0	6.0	14.0	20.0	29.0
FR	FL	3	**37.0**	10.0	4.0	11.0	7.0	31.0
FR	FL	4	45.0	5.0	1.0	7.0	12.0	29.0
FR	FL	5	47.0	13.0	1.0	16.0	5.0	18.0
FR	FL	6	44.0	14.0	4.0	9.0	12.0	18.0
FR	FL	7	42.0	10.0	8.0	15.0	8.0	18.0
FR	FL	8	48.0	11.0	4.0	13.0	11.0	13.0
FR	FL	9	55.0	16.0	1.0	9.0	7.0	12.0
FR	FL	10	51.0	13.0	3.0	10.0	3.0	21.0
FR	FL	11	54.0	10.0	0.0	10.0	4.0	21.0

FR	FL	12	51.0	16.0	7.0	10.0	1.0	13.0
FR	FL	13	60.0	14.0	2.0	11.0	5.0	8.0
FR	FL	14	71.0	11.0	2.0	3.0	5.0	8.0
FR	FL	15	57.0	12.0	4.0	13.0	4.0	9.0
FR	FL	16	49.0	13.0	7.0	12.0	7.0	11.0
FR	FL	17	62.0	13.0	7.0	9.0	3.0	7.0
FR	FL	18	65.0	8.0	4.0	7.0	5.0	11.0
FR	FL	19	55.0	10.0	4.0	14.0	4.0	14.0
FR	FL	20	66.0	8.0	4.0	7.0	2.0	13.0
FR	FL	21	70.0	6.0	4.0	11.0	1.0	8.0
FR	FL	22	69.0	9.0	2.0	5.0	3.0	12.0
FR	FL	23	66.0	10.0	5.0	9.0	4.0	6.0
FR	FL	24	59.0	14.0	5.0	9.0	3.0	10.0
FR	FL	25	61.0	15.0	6.0	5.0	4.0	10.0
FR	FL	26	61.0	12.0	11.0	2.0	4.0	8.0
FR	FL	27	62.0	11.0	3.0	6.0	1.0	17.0
FR	FL	28	64.0	13.0	7.0	8.0	4.0	3.0
FR	FL	29	63.0	12.0	9.0	4.0	2.0	11.0
FR	FL	30	58.0	12.0	9.0	9.0	5.0	8.0
FR	L	1	2.0	28.0	9.0	12.0	21.0	29.0
FR	L	2	0.0	**47.0**	12.0	3.0	10.0	28.0
FR	L	3	1.0	53.0	4.0	5.0	12.0	24.0
FR	L	4	0.0	52.0	10.0	7.0	10.0	19.0
FR	L	5	1.0	62.0	11.0	2.0	2.0	21.0
FR	L	6	1.0	53.0	15.0	6.0	12.0	13.0
FR	L	7	1.0	61.0	8.0	8.0	6.0	15.0
FR	L	8	0.0	61.0	14.0	5.0	7.0	12.0
FR	L	9	0.0	69.0	13.0	4.0	5.0	9.0
FR	L	10	0.0	73.0	9.0	4.0	3.0	11.0
FR	L	11	0.0	73.0	15.0	3.0	1.0	7.0
FR	L	12	1.0	67.0	13.0	5.0	3.0	11.0
FR	L	13	0.0	66.0	21.0	0.0	4.0	9.0
FR	L	14	1.0	70.0	16.0	1.0	2.0	9.0
FR	L	15	0.0	73.0	14.0	2.0	4.0	6.0
FR	L	16	1.0	62.0	20.0	2.0	2.0	12.0
FR	L	17	0.0	74.0	15.0	2.0	4.0	5.0
FR	L	18	0.0	69.0	12.0	2.0	9.0	9.0
FR	L	19	0.0	68.0	14.0	4.0	3.0	11.0
FR	L	20	1.0	71.0	12.0	2.0	2.0	11.0
FR	L	21	1.0	73.0	15.0	4.0	1.0	6.0

FR	L	22	0.0	68.0	18.0	0.0	5.0	9.0
FR	L	23	0.0	69.0	17.0	2.0	1.0	11.0
FR	L	24	0.0	72.0	20.0	0.0	4.0	5.0
FR	L	25	0.0	67.0	23.0	1.0	1.0	8.0
FR	L	26	1.0	71.0	19.0	0.0	4.0	6.0
FR	L	27	0.0	65.0	21.0	0.0	4.0	10.0
FR	L	28	0.0	68.0	17.0	2.0	6.0	8.0
FR	L	29	1.0	70.0	16.0	1.0	4.0	8.0
FR	L	30	0.0	67.0	22.0	0.0	2.0	9.0
FR	C	1	0.0	11.0	**45.0**	18.0	8.0	18.0
FR	C	2	0.0	11.0	56.0	17.0	10.0	6.0
FR	C	3	2.0	8.0	58.0	6.0	13.0	12.0
FR	C	4	0.0	14.0	55.0	7.0	14.0	11.0
FR	C	5	1.0	17.0	55.0	7.0	11.0	8.0
FR	C	6	0.0	16.0	63.0	5.0	8.0	8.0
FR	C	7	0.0	13.0	60.0	13.0	9.0	5.0
FR	C	8	0.0	10.0	58.0	8.0	17.0	8.0
FR	C	9	0.0	13.0	60.0	8.0	8.0	12.0
FR	C	10	0.0	19.0	59.0	9.0	9.0	5.0
FR	C	11	1.0	19.0	58.0	7.0	10.0	4.0
FR	C	12	1.0	15.0	61.0	11.0	7.0	5.0
FR	C	13	1.0	16.0	71.0	5.0	3.0	4.0
FR	C	14	0.0	20.0	56.0	5.0	12.0	8.0
FR	C	15	0.0	15.0	68.0	2.0	7.0	7.0
FR	C	16	0.0	13.0	71.0	5.0	7.0	4.0
FR	C	17	0.0	19.0	70.0	1.0	6.0	5.0
FR	C	18	0.0	20.0	66.0	0.0	9.0	5.0
FR	C	19	0.0	20.0	71.0	2.0	2.0	4.0
FR	C	20	1.0	16.0	66.0	3.0	8.0	6.0
FR	C	21	0.0	20.0	65.0	3.0	7.0	5.0
FR	C	22	0.0	21.0	67.0	2.0	6.0	4.0
FR	C	23	0.0	17.0	74.0	3.0	4.0	2.0
FR	C	24	0.0	16.0	74.0	1.0	4.0	4.0
FR	C	25	0.0	10.0	75.0	2.0	5.0	7.0
FR	C	26	0.0	8.0	80.0	2.0	6.0	3.0
FR	C	27	0.0	13.0	75.0	3.0	4.0	4.0
FR	C	28	0.0	16.0	65.0	2.0	12.0	5.0
FR	C	29	0.0	14.0	70.0	7.0	6.0	3.0
FR	C	30	0.0	12.0	75.0	3.0	7.0	3.0
FR	AW	1	0.0	3.0	11.0	**36.0**	20.0	30.0

FR	AW	2	0.0	8.0	3.0	51.0	18.0	21.0
FR	AW	3	0.0	6.0	5.0	58.0	14.0	16.0
FR	AW	4	0.0	6.0	6.0	49.0	19.0	20.0
FR	AW	5	2.0	5.0	0.0	68.0	15.0	11.0
FR	AW	6	0.0	4.0	7.0	61.0	17.0	11.0
FR	AW	7	0.0	4.0	8.0	58.0	9.0	22.0
FR	AW	8	1.0	7.0	4.0	56.0	17.0	15.0
FR	AW	9	1.0	1.0	4.0	62.0	14.0	18.0
FR	AW	10	0.0	4.0	8.0	56.0	14.0	17.0
FR	AW	11	0.0	3.0	3.0	64.0	16.0	14.0
FR	AW	12	0.0	7.0	4.0	64.0	13.0	12.0
FR	AW	13	0.0	0.0	5.0	65.0	15.0	15.0
FR	AW	14	0.0	5.0	3.0	64.0	16.0	12.0
FR	AW	15	0.0	8.0	8.0	58.0	17.0	10.0
FR	AW	16	0.0	8.0	5.0	61.0	13.0	12.0
FR	AW	17	0.0	4.0	5.0	65.0	14.0	12.0
FR	AW	18	0.0	10.0	6.0	62.0	12.0	9.0
FR	AW	19	1.0	4.0	4.0	67.0	11.0	12.0
FR	AW	20	0.0	6.0	3.0	68.0	8.0	15.0
FR	AW	21	0.0	1.0	8.0	56.0	21.0	14.0
FR	AW	22	1.0	6.0	3.0	63.0	11.0	15.0
FR	AW	23	0.0	6.0	5.0	65.0	12.0	13.0
FR	AW	24	0.0	1.0	4.0	72.0	12.0	11.0
FR	AW	25	0.0	7.0	6.0	60.0	15.0	13.0
FR	AW	26	1.0	4.0	8.0	63.0	15.0	9.0
FR	AW	27	0.0	4.0	5.0	64.0	8.0	18.0
FR	AW	28	1.0	3.0	5.0	73.0	12.0	6.0
FR	AW	29	1.0	6.0	8.0	63.0	8.0	14.0
FR	AW	30	0.0	5.0	5.0	60.0	17.0	13.0
FR	R	1	0.0	10.0	15.0	13.0	29.0	33.0
FR	R	2	0.0	8.0	16.0	4.0	36.0	36.0
FR	R	3	0.0	2.0	10.0	10.0	**48.0**	31.0
FR	R	4	0.0	3.0	20.0	8.0	37.0	31.0
FR	R	5	0.0	8.0	19.0	6.0	48.0	18.0
FR	R	6	0.0	7.0	7.0	7.0	56.0	22.0
FR	R	7	0.0	5.0	3.0	6.0	56.0	30.0
FR	R	8	0.0	1.0	10.0	8.0	61.0	20.0
FR	R	9	1.0	9.0	9.0	8.0	62.0	12.0
FR	R	10	0.0	4.0	18.0	10.0	48.0	21.0
FR	R	11	1.0	7.0	7.0	9.0	60.0	16.0

FR	R	12	0.0	10.0	17.0	9.0	51.0	13.0
FR	R	13	0.0	5.0	16.0	4.0	57.0	17.0
FR	R	14	1.0	6.0	15.0	8.0	56.0	13.0
FR	R	15	0.0	7.0	12.0	1.0	65.0	14.0
FR	R	16	0.0	12.0	9.0	6.0	54.0	18.0
FR	R	17	2.0	2.0	10.0	8.0	64.0	14.0
FR	R	18	0.0	5.0	19.0	7.0	60.0	8.0
FR	R	19	0.0	7.0	14.0	8.0	58.0	12.0
FR	R	20	0.0	11.0	14.0	1.0	56.0	18.0
FR	R	21	0.0	8.0	24.0	5.0	53.0	11.0
FR	R	22	0.0	6.0	26.0	3.0	46.0	19.0
FR	R	23	2.0	11.0	23.0	5.0	47.0	12.0
FR	R	24	1.0	8.0	21.0	3.0	60.0	8.0
FR	R	25	0.0	7.0	21.0	4.0	49.0	18.0
FR	R	26	0.0	14.0	30.0	4.0	44.0	8.0
FR	R	27	0.0	4.0	28.0	1.0	51.0	15.0
FR	R	28	0.0	6.0	27.0	4.0	55.0	8.0
FR	R	29	0.0	8.0	31.0	3.0	46.0	12.0
FR	R	30	1.0	15.0	24.0	1.0	46.0	12.0
FR	FR	1	0.0	0.0	7.0	16.0	20.0	57.0
FR	FR	2	2.0	2.0	2.0	5.0	28.0	60.0
FR	FR	3	0.0	15.0	8.0	7.0	10.0	60.0
FR	FR	4	0.0	8.0	12.0	10.0	23.0	47.0
FR	FR	5	0.0	4.0	7.0	9.0	25.0	56.0
FR	FR	6	0.0	8.0	3.0	5.0	29.0	55.0
FR	FR	7	2.0	4.0	9.0	9.0	15.0	61.0
FR	FR	8	0.0	12.0	3.0	3.0	23.0	58.0
FR	FR	9	0.0	5.0	11.0	2.0	16.0	66.0
FR	FR	10	0.0	2.0	16.0	4.0	22.0	55.0
FR	FR	11	0.0	12.0	9.0	9.0	14.0	55.0
FR	FR	12	0.0	5.0	2.0	14.0	20.0	59.0
FR	FR	13	0.0	7.0	7.0	4.0	18.0	64.0
FR	FR	14	0.0	9.0	4.0	11.0	29.0	47.0
FR	FR	15	0.0	11.0	4.0	6.0	13.0	66.0
FR	FR	16	2.0	4.0	11.0	2.0	13.0	69.0
FR	FR	17	0.0	5.0	9.0	11.0	23.0	52.0
FR	FR	18	2.0	2.0	4.0	7.0	17.0	67.0
FR	FR	19	0.0	3.0	8.0	5.0	24.0	61.0
FR	FR	20	0.0	0.0	7.0	4.0	33.0	56.0
FR	FR	21	0.0	11.0	11.0	4.0	21.0	54.0

FR	FR	22	0.0	4.0	9.0	4.0	22.0	61.0
FR	FR	23	0.0	9.0	10.0	3.0	26.0	52.0
FR	FR	24	0.0	11.0	4.0	0.0	16.0	70.0
FR	FR	25	2.0	9.0	11.0	2.0	16.0	59.0
FR	FR	26	0.0	9.0	18.0	5.0	12.0	55.0
FR	FR	27	0.0	2.0	11.0	13.0	16.0	58.0
FR	FR	28	0.0	10.0	8.0	4.0	21.0	56.0
FR	FR	29	0.0	8.0	22.0	2.0	16.0	53.0
FR	FR	30	0.0	6.0	11.0	3.0	11.0	69.0

Table S 15: Proportion of labelled videos falling under each political classification as a Far Right user begins to watch videos of a new political classification in the second stage of the experiment. Percentages highlighted in bold denote the video at which the proportion of videos labelled under the new class exceeded that of the original class.

Stage 3 Tables (Recommendation pathways)

Original Class	New Class	Video Number	Far Left	Left	Center	Anti-Woke	Right	Far Right
FL	FL	1	80.0	11.0	7.0	1.0	1.0	0.0
FL	FL	2	71.0	13.0	6.0	9.0	1.0	0.0
FL	FL	3	72.0	15.0	12.0	2.0	0.0	0.0
FL	FL	4	56.0	23.0	15.0	6.0	0.0	0.0
FL	FL	5	64.0	15.0	14.0	6.0	1.0	0.0
FL	FL	6	72.0	14.0	10.0	2.0	1.0	0.0
FL	FL	7	64.0	17.0	16.0	2.0	1.0	0.0
FL	FL	8	62.0	17.0	17.0	1.0	2.0	0.0
FL	FL	9	71.0	9.0	19.0	1.0	0.0	0.0
FL	FL	10	61.0	14.0	18.0	5.0	2.0	0.0
FL	FL	11	64.0	14.0	15.0	6.0	0.0	0.0
FL	FL	12	57.0	27.0	13.0	1.0	2.0	0.0
FL	FL	13	64.0	17.0	14.0	3.0	3.0	0.0
FL	FL	14	64.0	16.0	13.0	6.0	1.0	0.0
FL	FL	15	62.0	12.0	16.0	6.0	2.0	0.0
FL	FL	16	64.0	18.0	14.0	4.0	1.0	0.0
FL	FL	17	56.0	23.0	11.0	10.0	0.0	0.0
FL	FL	18	64.0	21.0	10.0	3.0	3.0	0.0
FL	FL	19	64.0	23.0	10.0	1.0	1.0	0.0
FL	FL	20	62.0	22.0	14.0	3.0	0.0	0.0
FL	FL	21	51.0	27.0	12.0	5.0	5.0	0.0
FL	FL	22	58.0	19.0	13.0	7.0	1.0	0.0
FL	FL	23	62.0	16.0	12.0	7.0	3.0	0.0
FL	FL	24	58.0	21.0	16.0	5.0	0.0	0.0
FL	FL	25	54.0	23.0	16.0	3.0	4.0	0.0
FL	FL	26	47.0	34.0	11.0	5.0	3.0	0.0
FL	FL	27	41.0	31.0	19.0	2.0	7.0	0.0
FL	FL	28	57.0	23.0	12.0	6.0	2.0	0.0
FL	FL	29	63.0	16.0	13.0	3.0	5.0	0.0
FL	FL	30	48.0	29.0	13.0	6.0	2.0	1.0
FL	L	1	14.0	58.0	24.0	2.0	1.0	0.0
FL	L	2	14.0	65.0	18.0	1.0	2.0	0.0
FL	L	3	11.0	64.0	21.0	2.0	1.0	0.0
FL	L	4	11.0	65.0	21.0	1.0	1.0	0.0
FL	L	5	12.0	63.0	21.0	2.0	1.0	0.0
FL	L	6	12.0	61.0	26.0	0.0	1.0	0.0

FL	L	7	11.0	59.0	24.0	3.0	1.0	1.0
FL	L	8	8.0	65.0	23.0	3.0	1.0	0.0
FL	L	9	15.0	62.0	17.0	2.0	3.0	0.0
FL	L	10	10.0	69.0	16.0	2.0	2.0	0.0
FL	L	11	16.0	63.0	17.0	3.0	1.0	0.0
FL	L	12	20.0	58.0	20.0	1.0	1.0	0.0
FL	L	13	16.0	60.0	23.0	1.0	0.0	0.0
FL	L	14	13.0	71.0	15.0	0.0	1.0	0.0
FL	L	15	18.0	57.0	21.0	4.0	0.0	0.0
FL	L	16	19.0	64.0	15.0	1.0	0.0	0.0
FL	L	17	16.0	59.0	23.0	1.0	0.0	0.0
FL	L	18	23.0	59.0	15.0	2.0	0.0	0.0
FL	L	19	18.0	62.0	15.0	6.0	0.0	0.0
FL	L	20	23.0	61.0	13.0	3.0	0.0	0.0
FL	L	21	14.0	69.0	16.0	1.0	0.0	0.0
FL	L	22	25.0	58.0	13.0	1.0	3.0	0.0
FL	L	23	12.0	61.0	20.0	5.0	1.0	0.0
FL	L	24	10.0	58.0	24.0	4.0	2.0	1.0
FL	L	25	17.0	57.0	20.0	2.0	3.0	0.0
FL	L	26	16.0	57.0	23.0	2.0	1.0	0.0
FL	L	27	16.0	62.0	20.0	0.0	2.0	0.0
FL	L	28	17.0	58.0	22.0	2.0	1.0	0.0
FL	L	29	16.0	56.0	27.0	1.0	0.0	0.0
FL	L	30	21.0	59.0	17.0	0.0	3.0	0.0
FL	C	1	5.0	18.0	69.0	4.0	5.0	0.0
FL	C	2	4.0	19.0	71.0	1.0	5.0	0.0
FL	C	3	5.0	25.0	64.0	2.0	3.0	0.0
FL	C	4	8.0	22.0	65.0	2.0	3.0	0.0
FL	C	5	8.0	21.0	66.0	4.0	1.0	0.0
FL	C	6	12.0	19.0	65.0	0.0	4.0	0.0
FL	C	7	6.0	18.0	71.0	1.0	3.0	0.0
FL	C	8	11.0	22.0	61.0	1.0	5.0	0.0
FL	C	9	10.0	27.0	56.0	2.0	5.0	0.0
FL	C	10	8.0	25.0	64.0	1.0	2.0	0.0
FL	C	11	12.0	21.0	62.0	1.0	5.0	0.0
FL	C	12	4.0	26.0	66.0	0.0	4.0	0.0
FL	C	13	6.0	24.0	61.0	3.0	5.0	0.0
FL	C	14	6.0	25.0	65.0	0.0	4.0	0.0
FL	C	15	12.0	17.0	62.0	4.0	6.0	0.0
FL	C	16	8.0	28.0	57.0	1.0	6.0	0.0

FL	C	17	7.0	21.0	65.0	0.0	6.0	0.0
FL	C	18	8.0	17.0	67.0	2.0	7.0	0.0
FL	C	19	6.0	19.0	73.0	0.0	2.0	0.0
FL	C	20	7.0	21.0	65.0	1.0	6.0	0.0
FL	C	21	14.0	22.0	58.0	0.0	7.0	0.0
FL	C	22	8.0	23.0	63.0	1.0	5.0	0.0
FL	C	23	11.0	25.0	55.0	3.0	6.0	0.0
FL	C	24	10.0	20.0	69.0	1.0	0.0	0.0
FL	C	25	8.0	31.0	56.0	1.0	4.0	0.0
FL	C	26	13.0	20.0	63.0	0.0	3.0	0.0
FL	C	27	9.0	18.0	69.0	0.0	4.0	0.0
FL	C	28	13.0	18.0	68.0	0.0	1.0	0.0
FL	C	29	13.0	14.0	71.0	0.0	1.0	0.0
FL	C	30	6.0	21.0	68.0	2.0	2.0	0.0

FL	AW	1	16.0	12.0	2.0	60.0	9.0	0.0
FL	AW	2	15.0	11.0	11.0	59.0	3.0	1.0
FL	AW	3	20.0	8.0	6.0	62.0	4.0	0.0
FL	AW	4	22.0	10.0	11.0	46.0	11.0	0.0
FL	AW	5	12.0	5.0	10.0	55.0	15.0	3.0
FL	AW	6	14.0	6.0	8.0	61.0	10.0	0.0
FL	AW	7	8.0	13.0	15.0	58.0	6.0	0.0
FL	AW	8	9.0	22.0	12.0	50.0	6.0	1.0
FL	AW	9	8.0	14.0	14.0	52.0	11.0	0.0
FL	AW	10	7.0	24.0	16.0	47.0	7.0	0.0
FL	AW	11	17.0	15.0	18.0	44.0	5.0	1.0
FL	AW	12	7.0	22.0	14.0	46.0	10.0	0.0
FL	AW	13	2.0	23.0	16.0	53.0	6.0	0.0
FL	AW	14	5.0	15.0	11.0	61.0	8.0	0.0
FL	AW	15	6.0	9.0	20.0	59.0	4.0	1.0
FL	AW	16	7.0	13.0	27.0	41.0	9.0	3.0
FL	AW	17	8.0	15.0	18.0	48.0	9.0	2.0
FL	AW	18	3.0	17.0	24.0	51.0	5.0	0.0
FL	AW	19	8.0	17.0	30.0	40.0	5.0	0.0
FL	AW	20	9.0	13.0	21.0	45.0	10.0	1.0
FL	AW	21	7.0	18.0	15.0	49.0	11.0	0.0
FL	AW	22	14.0	13.0	17.0	51.0	3.0	1.0
FL	AW	23	5.0	19.0	27.0	45.0	4.0	0.0
FL	AW	24	9.0	14.0	16.0	50.0	11.0	0.0
FL	AW	25	15.0	9.0	22.0	40.0	14.0	0.0
FL	AW	26	12.0	16.0	23.0	44.0	5.0	0.0

FL	AW	27	11.0	16.0	21.0	46.0	5.0	2.0
FL	AW	28	13.0	12.0	26.0	44.0	4.0	0.0
FL	AW	29	12.0	13.0	19.0	48.0	6.0	1.0
FL	AW	30	13.0	14.0	22.0	47.0	4.0	0.0
FL	R	1	16.0	15.0	28.0	3.0	37.0	1.0
FL	R	2	13.0	23.0	25.0	6.0	33.0	0.0
FL	R	3	9.0	32.0	32.0	3.0	25.0	0.0
FL	R	4	15.0	23.0	32.0	6.0	24.0	0.0
FL	R	5	9.0	26.0	40.0	2.0	22.0	0.0
FL	R	6	9.0	32.0	38.0	4.0	15.0	1.0
FL	R	7	8.0	33.0	29.0	0.0	29.0	1.0
FL	R	8	4.0	32.0	39.0	3.0	22.0	0.0
FL	R	9	6.0	26.0	35.0	1.0	32.0	0.0
FL	R	10	12.0	25.0	30.0	6.0	25.0	1.0
FL	R	11	12.0	26.0	30.0	4.0	29.0	0.0
FL	R	12	8.0	30.0	29.0	8.0	24.0	1.0
FL	R	13	10.0	23.0	43.0	4.0	21.0	0.0
FL	R	14	6.0	24.0	36.0	7.0	25.0	1.0
FL	R	15	11.0	24.0	38.0	1.0	25.0	1.0
FL	R	16	5.0	26.0	29.0	7.0	31.0	2.0
FL	R	17	8.0	18.0	43.0	3.0	26.0	1.0
FL	R	18	13.0	36.0	21.0	6.0	22.0	1.0
FL	R	19	9.0	27.0	27.0	3.0	34.0	1.0
FL	R	20	8.0	24.0	33.0	6.0	29.0	0.0
FL	R	21	7.0	17.0	42.0	9.0	25.0	0.0
FL	R	22	10.0	23.0	40.0	5.0	22.0	0.0
FL	R	23	5.0	30.0	35.0	4.0	24.0	1.0
FL	R	24	9.0	20.0	34.0	9.0	28.0	0.0
FL	R	25	8.0	25.0	35.0	0.0	31.0	1.0
FL	R	26	6.0	30.0	30.0	6.0	28.0	0.0
FL	R	27	7.0	20.0	39.0	7.0	27.0	1.0
FL	R	28	12.0	14.0	34.0	8.0	32.0	0.0
FL	R	29	8.0	26.0	37.0	1.0	28.0	0.0
FL	R	30	9.0	23.0	40.0	3.0	23.0	2.0
FL	FR	1	31.0	21.0	17.0	10.0	2.0	19.0
FL	FR	2	46.0	8.0	5.0	10.0	3.0	28.0
FL	FR	3	32.0	15.0	12.0	3.0	8.0	29.0
FL	FR	4	22.0	18.0	15.0	7.0	13.0	25.0
FL	FR	5	36.0	8.0	23.0	8.0	8.0	19.0
FL	FR	6	33.0	16.0	12.0	14.0	12.0	14.0

FL	FR	7	33.0	11.0	13.0	13.0	7.0	24.0
FL	FR	8	35.0	17.0	11.0	7.0	6.0	24.0
FL	FR	9	33.0	19.0	21.0	10.0	2.0	15.0
FL	FR	10	43.0	6.0	6.0	6.0	14.0	24.0
FL	FR	11	26.0	13.0	17.0	9.0	15.0	21.0
FL	FR	12	32.0	5.0	18.0	2.0	18.0	25.0
FL	FR	13	20.0	22.0	9.0	9.0	9.0	31.0
FL	FR	14	26.0	15.0	20.0	9.0	13.0	17.0
FL	FR	15	27.0	16.0	16.0	11.0	13.0	18.0
FL	FR	16	29.0	19.0	15.0	8.0	6.0	23.0
FL	FR	17	28.0	14.0	21.0	9.0	12.0	16.0
FL	FR	18	30.0	17.0	7.0	13.0	11.0	22.0
FL	FR	19	26.0	14.0	20.0	6.0	23.0	11.0
FL	FR	20	30.0	14.0	12.0	9.0	12.0	23.0
FL	FR	21	42.0	13.0	20.0	4.0	4.0	16.0
FL	FR	22	26.0	9.0	33.0	14.0	7.0	12.0
FL	FR	23	30.0	30.0	12.0	0.0	15.0	12.0
FL	FR	24	33.0	21.0	12.0	9.0	12.0	14.0
FL	FR	25	29.0	20.0	12.0	10.0	10.0	20.0
FL	FR	26	34.0	20.0	16.0	2.0	9.0	18.0
FL	FR	27	18.0	25.0	7.0	11.0	11.0	29.0
FL	FR	28	32.0	18.0	9.0	11.0	7.0	23.0
FL	FR	29	18.0	11.0	21.0	12.0	9.0	29.0
FL	FR	30	27.0	18.0	20.0	6.0	4.0	25.0

Table S 16: Proportion of labelled videos falling under each political classification as a user who has watched 30 videos of the “Original class” and then 30 videos of the “New Class” begins to watch the top recommended video in the third stage of the experiment.

**Original Class**	**New Class**	**Video Number**	**Far Left**	**Left**	**Center**	**Anti-Woke**	**Right**	**Far Right**
L	FL	1	47.0	39.0	11.0	2.0	1.0	0.0
L	FL	2	43.0	47.0	8.0	1.0	0.0	1.0
L	FL	3	38.0	51.0	9.0	1.0	0.0	1.0
L	FL	4	35.0	49.0	15.0	0.0	1.0	0.0
L	FL	5	38.0	42.0	13.0	4.0	2.0	1.0
L	FL	6	40.0	46.0	12.0	1.0	1.0	0.0
L	FL	7	39.0	47.0	11.0	1.0	2.0	0.0
L	FL	8	31.0	55.0	12.0	1.0	1.0	0.0
L	FL	9	36.0	48.0	14.0	0.0	2.0	0.0

L	FL	10	34.0	48.0	11.0	2.0	3.0	0.0
L	FL	11	31.0	51.0	13.0	2.0	4.0	0.0
L	FL	12	34.0	52.0	11.0	1.0	2.0	0.0
L	FL	13	32.0	55.0	12.0	0.0	1.0	0.0
L	FL	14	31.0	48.0	17.0	1.0	2.0	0.0
L	FL	15	31.0	48.0	16.0	3.0	1.0	0.0
L	FL	16	26.0	50.0	17.0	0.0	7.0	0.0
L	FL	17	30.0	51.0	18.0	0.0	1.0	0.0
L	FL	18	36.0	46.0	14.0	1.0	3.0	0.0
L	FL	19	41.0	47.0	12.0	0.0	0.0	0.0
L	FL	20	22.0	53.0	18.0	1.0	6.0	0.0
L	FL	21	26.0	50.0	23.0	0.0	1.0	0.0
L	FL	22	29.0	52.0	17.0	2.0	0.0	0.0
L	FL	23	41.0	45.0	13.0	0.0	1.0	0.0
L	FL	24	37.0	46.0	11.0	1.0	4.0	1.0
L	FL	25	27.0	58.0	14.0	0.0	0.0	0.0
L	FL	26	32.0	47.0	19.0	0.0	1.0	0.0
L	FL	27	25.0	58.0	15.0	1.0	1.0	0.0
L	FL	28	27.0	51.0	20.0	0.0	1.0	0.0
L	FL	29	21.0	61.0	14.0	2.0	1.0	0.0
L	FL	30	27.0	57.0	13.0	1.0	1.0	0.0
L	L	1	0.0	81.0	18.0	0.0	1.0	0.0
L	L	2	1.0	76.0	21.0	0.0	2.0	0.0
L	L	3	1.0	72.0	24.0	0.0	3.0	0.0
L	L	4	0.0	78.0	20.0	1.0	1.0	0.0
L	L	5	0.0	75.0	24.0	0.0	1.0	0.0
L	L	6	0.0	74.0	25.0	0.0	1.0	0.0
L	L	7	0.0	70.0	28.0	0.0	2.0	0.0
L	L	8	1.0	76.0	23.0	0.0	0.0	0.0
L	L	9	0.0	72.0	25.0	1.0	2.0	0.0
L	L	10	1.0	75.0	20.0	0.0	4.0	0.0
L	L	11	1.0	77.0	21.0	0.0	1.0	0.0
L	L	12	0.0	80.0	18.0	1.0	1.0	0.0
L	L	13	0.0	71.0	26.0	0.0	3.0	0.0
L	L	14	0.0	76.0	21.0	2.0	1.0	0.0
L	L	15	0.0	72.0	24.0	0.0	3.0	0.0
L	L	16	1.0	83.0	15.0	0.0	1.0	0.0
L	L	17	1.0	72.0	25.0	1.0	1.0	0.0
L	L	18	0.0	80.0	19.0	0.0	1.0	0.0
L	L	19	0.0	75.0	22.0	0.0	3.0	0.0

L	L	20	0.0	77.0	20.0	0.0	2.0	1.0
L	L	21	0.0	82.0	16.0	0.0	2.0	0.0
L	L	22	0.0	78.0	21.0	0.0	1.0	0.0
L	L	23	0.0	78.0	22.0	0.0	0.0	0.0
L	L	24	0.0	75.0	24.0	0.0	1.0	0.0
L	L	25	1.0	75.0	23.0	0.0	2.0	0.0
L	L	26	0.0	79.0	20.0	0.0	1.0	0.0
L	L	27	1.0	69.0	27.0	0.0	4.0	0.0
L	L	28	1.0	76.0	21.0	1.0	1.0	0.0
L	L	29	0.0	74.0	23.0	0.0	3.0	0.0
L	L	30	0.0	84.0	15.0	0.0	1.0	0.0
L	C	1	0.0	39.0	59.0	1.0	1.0	0.0
L	C	2	1.0	41.0	54.0	0.0	4.0	0.0
L	C	3	0.0	34.0	62.0	2.0	3.0	0.0
L	C	4	0.0	37.0	60.0	0.0	4.0	0.0
L	C	5	0.0	45.0	51.0	1.0	3.0	0.0
L	C	6	0.0	30.0	66.0	0.0	4.0	0.0
L	C	7	0.0	41.0	57.0	0.0	2.0	0.0
L	C	8	0.0	46.0	51.0	0.0	4.0	0.0
L	C	9	0.0	34.0	66.0	0.0	0.0	0.0
L	C	10	0.0	45.0	53.0	0.0	3.0	0.0
L	C	11	0.0	32.0	63.0	1.0	4.0	0.0
L	C	12	0.0	40.0	57.0	0.0	3.0	0.0
L	C	13	0.0	37.0	59.0	1.0	3.0	0.0
L	C	14	0.0	33.0	65.0	1.0	1.0	0.0
L	C	15	0.0	38.0	60.0	0.0	2.0	0.0
L	C	16	0.0	34.0	60.0	2.0	4.0	0.0
L	C	17	0.0	38.0	59.0	1.0	2.0	0.0
L	C	18	0.0	39.0	54.0	2.0	5.0	0.0
L	C	19	0.0	33.0	62.0	2.0	3.0	0.0
L	C	20	0.0	42.0	56.0	1.0	1.0	0.0
L	C	21	0.0	31.0	64.0	0.0	5.0	0.0
L	C	22	0.0	38.0	61.0	0.0	1.0	0.0
L	C	23	0.0	39.0	60.0	0.0	1.0	0.0
L	C	24	0.0	32.0	63.0	1.0	4.0	0.0
L	C	25	0.0	42.0	57.0	1.0	0.0	0.0
L	C	26	0.0	35.0	64.0	0.0	1.0	0.0
L	C	27	0.0	36.0	64.0	1.0	0.0	0.0
L	C	28	0.0	39.0	60.0	0.0	1.0	0.0
L	C	29	1.0	39.0	56.0	1.0	3.0	0.0

L	C	30	0.0	41.0	58.0	1.0	0.0	0.0
L	AW	1	0.0	30.0	7.0	55.0	7.0	0.0
L	AW	2	1.0	35.0	8.0	45.0	11.0	0.0
L	AW	3	0.0	27.0	7.0	57.0	7.0	1.0
L	AW	4	0.0	26.0	12.0	51.0	10.0	1.0
L	AW	5	0.0	33.0	14.0	46.0	6.0	1.0
L	AW	6	0.0	35.0	6.0	49.0	9.0	0.0
L	AW	7	1.0	30.0	15.0	45.0	9.0	0.0
L	AW	8	2.0	38.0	12.0	41.0	6.0	0.0
L	AW	9	0.0	32.0	16.0	44.0	7.0	0.0
L	AW	10	1.0	30.0	13.0	45.0	11.0	0.0
L	AW	11	1.0	31.0	14.0	44.0	10.0	0.0
L	AW	12	0.0	43.0	15.0	39.0	4.0	0.0
L	AW	13	1.0	37.0	9.0	45.0	9.0	0.0
L	AW	14	0.0	39.0	13.0	40.0	7.0	0.0
L	AW	15	0.0	38.0	11.0	44.0	5.0	1.0
L	AW	16	0.0	37.0	9.0	47.0	7.0	0.0
L	AW	17	1.0	26.0	17.0	44.0	12.0	0.0
L	AW	18	0.0	43.0	18.0	33.0	6.0	0.0
L	AW	19	0.0	34.0	14.0	42.0	10.0	0.0
L	AW	20	1.0	38.0	12.0	40.0	9.0	0.0
L	AW	21	0.0	26.0	15.0	49.0	10.0	0.0
L	AW	22	0.0	38.0	10.0	45.0	6.0	0.0
L	AW	23	1.0	43.0	8.0	38.0	10.0	0.0
L	AW	24	0.0	43.0	8.0	44.0	5.0	0.0
L	AW	25	0.0	35.0	17.0	42.0	6.0	0.0
L	AW	26	1.0	42.0	12.0	36.0	7.0	1.0
L	AW	27	0.0	38.0	11.0	46.0	5.0	0.0
L	AW	28	0.0	37.0	6.0	46.0	11.0	0.0
L	AW	29	1.0	49.0	5.0	38.0	7.0	0.0
L	AW	30	0.0	33.0	15.0	43.0	8.0	1.0
L	R	1	1.0	32.0	30.0	4.0	33.0	0.0
L	R	2	0.0	44.0	31.0	4.0	20.0	1.0
L	R	3	0.0	43.0	22.0	1.0	33.0	0.0
L	R	4	0.0	44.0	25.0	0.0	31.0	0.0
L	R	5	0.0	44.0	29.0	2.0	26.0	0.0
L	R	6	0.0	39.0	34.0	0.0	28.0	0.0
L	R	7	0.0	48.0	26.0	1.0	26.0	0.0
L	R	8	0.0	43.0	36.0	2.0	20.0	0.0
L	R	9	0.0	55.0	27.0	0.0	19.0	0.0

L	R	10	0.0	40.0	33.0	2.0	26.0	0.0
L	R	11	1.0	39.0	38.0	1.0	21.0	0.0
L	R	12	1.0	52.0	20.0	2.0	25.0	0.0
L	R	13	0.0	53.0	24.0	0.0	23.0	0.0
L	R	14	0.0	43.0	27.0	0.0	31.0	0.0
L	R	15	1.0	43.0	32.0	0.0	23.0	1.0
L	R	16	1.0	56.0	20.0	0.0	22.0	1.0
L	R	17	0.0	45.0	26.0	3.0	25.0	0.0
L	R	18	0.0	47.0	30.0	2.0	20.0	0.0
L	R	19	0.0	44.0	31.0	0.0	26.0	0.0
L	R	20	0.0	46.0	24.0	1.0	28.0	0.0
L	R	21	0.0	56.0	22.0	0.0	22.0	0.0
L	R	22	0.0	56.0	17.0	0.0	24.0	3.0
L	R	23	0.0	48.0	27.0	2.0	23.0	0.0
L	R	24	0.0	45.0	30.0	0.0	25.0	0.0
L	R	25	0.0	52.0	24.0	1.0	24.0	0.0
L	R	26	0.0	46.0	33.0	1.0	20.0	0.0
L	R	27	0.0	41.0	26.0	0.0	33.0	0.0
L	R	28	0.0	48.0	32.0	1.0	19.0	0.0
L	R	29	0.0	44.0	30.0	1.0	26.0	0.0
L	R	30	0.0	52.0	25.0	0.0	23.0	0.0
L	FR	1	0.0	48.0	20.0	0.0	11.0	21.0
L	FR	2	0.0	43.0	20.0	2.0	14.0	20.0
L	FR	3	0.0	45.0	22.0	2.0	18.0	14.0
L	FR	4	0.0	55.0	15.0	0.0	12.0	18.0
L	FR	5	0.0	49.0	23.0	0.0	10.0	18.0
L	FR	6	0.0	59.0	20.0	2.0	5.0	15.0
L	FR	7	2.0	43.0	32.0	4.0	4.0	15.0
L	FR	8	3.0	44.0	23.0	0.0	15.0	15.0
L	FR	9	0.0	63.0	16.0	2.0	9.0	9.0
L	FR	10	0.0	51.0	21.0	5.0	10.0	13.0
L	FR	11	0.0	55.0	25.0	2.0	2.0	15.0
L	FR	12	0.0	49.0	24.0	2.0	9.0	16.0
L	FR	13	0.0	59.0	25.0	5.0	5.0	7.0
L	FR	14	0.0	53.0	23.0	0.0	12.0	12.0
L	FR	15	0.0	63.0	15.0	2.0	8.0	12.0
L	FR	16	0.0	56.0	23.0	0.0	5.0	16.0
L	FR	17	0.0	57.0	22.0	2.0	5.0	12.0
L	FR	18	0.0	60.0	13.0	0.0	4.0	23.0
L	FR	19	0.0	70.0	14.0	2.0	5.0	9.0

L	FR	20	5.0	59.0	16.0	0.0	5.0	14.0
L	FR	21	3.0	58.0	22.0	0.0	6.0	11.0
L	FR	22	0.0	51.0	29.0	0.0	11.0	9.0
L	FR	23	2.0	43.0	25.0	2.0	7.0	20.0
L	FR	24	2.0	57.0	19.0	2.0	0.0	19.0
L	FR	25	0.0	48.0	31.0	0.0	4.0	17.0
L	FR	26	0.0	54.0	24.0	2.0	2.0	18.0
L	FR	27	0.0	60.0	23.0	2.0	4.0	11.0
L	FR	28	0.0	65.0	14.0	2.0	4.0	14.0
L	FR	29	0.0	57.0	20.0	2.0	2.0	18.0
L	FR	30	0.0	60.0	24.0	2.0	5.0	10.0

Table S 17: Proportion of labelled videos falling under each political classification as a user who has watched 30 videos of the “Original class” and then 30 videos of the “New Class” begins to watch the top recommended video in the third stage of the experiment.

**Original Class**	**New Class**	**Video Number**	**Far Left**	**Left**	**Center**	**Anti-Woke**	**Right**	**Far Right**
C	FL	1	46.0	19.0	27.0	5.0	2.0	0.0
C	FL	2	42.0	19.0	36.0	2.0	2.0	0.0
C	FL	3	29.0	16.0	47.0	4.0	3.0	0.0
C	FL	4	23.0	24.0	45.0	2.0	5.0	0.0
C	FL	5	38.0	18.0	34.0	6.0	3.0	0.0
C	FL	6	26.0	18.0	46.0	4.0	5.0	0.0
C	FL	7	31.0	22.0	34.0	8.0	5.0	0.0
C	FL	8	33.0	23.0	40.0	2.0	2.0	0.0
C	FL	9	24.0	26.0	41.0	7.0	2.0	0.0
C	FL	10	27.0	22.0	44.0	1.0	5.0	0.0
C	FL	11	38.0	20.0	36.0	0.0	5.0	1.0
C	FL	12	23.0	28.0	44.0	1.0	4.0	0.0
C	FL	13	21.0	14.0	49.0	10.0	6.0	0.0
C	FL	14	29.0	28.0	36.0	4.0	4.0	0.0
C	FL	15	24.0	16.0	41.0	5.0	14.0	0.0
C	FL	16	18.0	24.0	43.0	7.0	7.0	1.0
C	FL	17	31.0	18.0	43.0	2.0	6.0	0.0
C	FL	18	24.0	19.0	46.0	5.0	5.0	0.0
C	FL	19	27.0	23.0	36.0	3.0	10.0	0.0
C	FL	20	29.0	25.0	39.0	2.0	5.0	0.0
C	FL	21	20.0	28.0	44.0	4.0	5.0	0.0
C	FL	22	23.0	21.0	49.0	4.0	4.0	0.0

C	FL	23	23.0	23.0	48.0	2.0	3.0	0.0
C	FL	24	23.0	26.0	39.0	5.0	7.0	0.0
C	FL	25	31.0	33.0	28.0	4.0	2.0	1.0
C	FL	26	29.0	25.0	37.0	2.0	7.0	0.0
C	FL	27	21.0	23.0	47.0	2.0	7.0	0.0
C	FL	28	22.0	21.0	44.0	6.0	7.0	0.0
C	FL	29	22.0	29.0	41.0	1.0	6.0	0.0
C	FL	30	26.0	22.0	45.0	4.0	4.0	0.0
C	L	1	0.0	49.0	49.0	1.0	1.0	0.0
C	L	2	1.0	55.0	41.0	2.0	1.0	0.0
C	L	3	0.0	48.0	50.0	2.0	0.0	0.0
C	L	4	0.0	39.0	56.0	1.0	3.0	0.0
C	L	5	0.0	49.0	50.0	0.0	1.0	0.0
C	L	6	0.0	45.0	53.0	0.0	1.0	0.0
C	L	7	0.0	60.0	37.0	1.0	2.0	0.0
C	L	8	0.0	47.0	52.0	1.0	0.0	0.0
C	L	9	0.0	44.0	52.0	3.0	1.0	0.0
C	L	10	1.0	52.0	46.0	0.0	1.0	0.0
C	L	11	0.0	42.0	55.0	0.0	3.0	0.0
C	L	12	0.0	48.0	49.0	2.0	1.0	0.0
C	L	13	0.0	52.0	43.0	0.0	5.0	0.0
C	L	14	1.0	47.0	52.0	0.0	0.0	0.0
C	L	15	0.0	49.0	48.0	0.0	3.0	0.0
C	L	16	1.0	45.0	51.0	2.0	1.0	0.0
C	L	17	0.0	40.0	56.0	2.0	2.0	0.0
C	L	18	0.0	43.0	53.0	0.0	4.0	0.0
C	L	19	0.0	49.0	46.0	1.0	3.0	0.0
C	L	20	1.0	43.0	54.0	1.0	1.0	0.0
C	L	21	1.0	42.0	54.0	0.0	3.0	0.0
C	L	22	0.0	37.0	60.0	1.0	2.0	0.0
C	L	23	0.0	41.0	54.0	1.0	3.0	0.0
C	L	24	0.0	50.0	49.0	1.0	0.0	0.0
C	L	25	0.0	44.0	53.0	1.0	2.0	0.0
C	L	26	0.0	48.0	52.0	0.0	0.0	0.0
C	L	27	0.0	45.0	54.0	0.0	1.0	0.0
C	L	28	0.0	50.0	50.0	0.0	0.0	0.0
C	L	29	0.0	49.0	47.0	1.0	3.0	0.0
C	L	30	0.0	42.0	57.0	0.0	1.0	0.0
C	C	1	0.0	14.0	82.0	0.0	3.0	1.0
C	C	2	0.0	14.0	81.0	1.0	3.0	0.0

C	C	3	0.0	19.0	76.0	0.0	5.0	0.0
C	C	4	0.0	11.0	85.0	2.0	2.0	0.0
C	C	5	1.0	13.0	84.0	1.0	1.0	0.0
C	C	6	1.0	15.0	81.0	2.0	2.0	0.0
C	C	7	3.0	19.0	71.0	0.0	7.0	0.0
C	C	8	1.0	12.0	82.0	1.0	4.0	0.0
C	C	9	1.0	11.0	82.0	2.0	5.0	0.0
C	C	10	1.0	14.0	79.0	3.0	4.0	0.0
C	C	11	2.0	21.0	70.0	2.0	5.0	1.0
C	C	12	1.0	13.0	79.0	2.0	5.0	0.0
C	C	13	1.0	18.0	76.0	1.0	4.0	0.0
C	C	14	0.0	17.0	80.0	1.0	2.0	0.0
C	C	15	0.0	18.0	80.0	1.0	1.0	0.0
C	C	16	0.0	20.0	73.0	2.0	5.0	0.0
C	C	17	0.0	18.0	77.0	2.0	3.0	0.0
C	C	18	0.0	12.0	81.0	0.0	6.0	0.0
C	C	19	0.0	21.0	71.0	1.0	7.0	0.0
C	C	20	1.0	13.0	79.0	3.0	5.0	0.0
C	C	21	0.0	24.0	68.0	2.0	6.0	0.0
C	C	22	0.0	20.0	77.0	0.0	3.0	0.0
C	C	23	0.0	15.0	78.0	1.0	6.0	0.0
C	C	24	0.0	14.0	82.0	2.0	2.0	0.0
C	C	25	0.0	15.0	80.0	1.0	4.0	0.0
C	C	26	0.0	21.0	75.0	0.0	3.0	1.0
C	C	27	0.0	16.0	79.0	2.0	4.0	0.0
C	C	28	0.0	14.0	82.0	2.0	2.0	0.0
C	C	29	0.0	11.0	86.0	0.0	3.0	0.0
C	C	30	0.0	19.0	77.0	1.0	3.0	0.0
C	AW	1	0.0	16.0	34.0	47.0	2.0	0.0
C	AW	2	0.0	13.0	35.0	44.0	8.0	0.0
C	AW	3	3.0	8.0	38.0	43.0	7.0	0.0
C	AW	4	0.0	22.0	41.0	34.0	3.0	0.0
C	AW	5	1.0	18.0	38.0	36.0	7.0	0.0
C	AW	6	0.0	13.0	47.0	29.0	10.0	0.0
C	AW	7	2.0	18.0	45.0	32.0	2.0	2.0
C	AW	8	3.0	14.0	32.0	42.0	10.0	0.0
C	AW	9	0.0	11.0	40.0	37.0	11.0	0.0
C	AW	10	0.0	16.0	44.0	35.0	5.0	0.0
C	AW	11	1.0	11.0	53.0	29.0	5.0	0.0
C	AW	12	0.0	15.0	47.0	36.0	3.0	0.0

C	AW	13	0.0	15.0	47.0	28.0	9.0	0.0
C	AW	14	0.0	13.0	52.0	27.0	6.0	2.0
C	AW	15	1.0	12.0	46.0	35.0	5.0	0.0
C	AW	16	0.0	19.0	43.0	32.0	5.0	1.0
C	AW	17	0.0	12.0	49.0	35.0	3.0	1.0
C	AW	18	1.0	14.0	54.0	23.0	7.0	0.0
C	AW	19	0.0	24.0	46.0	27.0	3.0	0.0
C	AW	20	1.0	9.0	45.0	35.0	9.0	0.0
C	AW	21	0.0	17.0	48.0	32.0	3.0	0.0
C	AW	22	0.0	17.0	44.0	33.0	5.0	0.0
C	AW	23	0.0	15.0	51.0	27.0	4.0	3.0
C	AW	24	0.0	11.0	44.0	33.0	11.0	0.0
C	AW	25	0.0	18.0	47.0	31.0	4.0	0.0
C	AW	26	0.0	11.0	59.0	21.0	9.0	0.0
C	AW	27	0.0	13.0	48.0	29.0	9.0	0.0
C	AW	28	0.0	9.0	57.0	28.0	6.0	0.0
C	AW	29	0.0	20.0	48.0	26.0	6.0	0.0
C	AW	30	0.0	10.0	54.0	29.0	6.0	0.0
C	R	1	0.0	17.0	44.0	2.0	36.0	1.0
C	R	2	0.0	20.0	50.0	2.0	28.0	0.0
C	R	3	0.0	27.0	51.0	3.0	19.0	0.0
C	R	4	0.0	19.0	47.0	1.0	33.0	0.0
C	R	5	1.0	19.0	42.0	0.0	38.0	0.0
C	R	6	1.0	28.0	47.0	1.0	23.0	0.0
C	R	7	0.0	25.0	42.0	2.0	30.0	0.0
C	R	8	0.0	21.0	48.0	2.0	29.0	0.0
C	R	9	0.0	19.0	56.0	4.0	20.0	0.0
C	R	10	0.0	26.0	48.0	0.0	27.0	0.0
C	R	11	0.0	26.0	47.0	2.0	24.0	0.0
C	R	12	0.0	28.0	43.0	2.0	27.0	0.0
C	R	13	0.0	22.0	47.0	4.0	26.0	1.0
C	R	14	0.0	24.0	47.0	1.0	27.0	0.0
C	R	15	1.0	26.0	48.0	1.0	24.0	0.0
C	R	16	0.0	24.0	40.0	1.0	34.0	1.0
C	R	17	2.0	22.0	56.0	1.0	19.0	0.0
C	R	18	0.0	24.0	55.0	2.0	20.0	0.0
C	R	19	0.0	21.0	48.0	3.0	28.0	0.0
C	R	20	0.0	34.0	44.0	3.0	19.0	0.0
C	R	21	1.0	22.0	53.0	1.0	23.0	0.0
C	R	22	0.0	26.0	49.0	2.0	23.0	0.0

C	R	23	0.0	24.0	55.0	0.0	21.0	0.0
C	R	24	0.0	30.0	49.0	3.0	18.0	0.0
C	R	25	1.0	32.0	47.0	2.0	18.0	0.0
C	R	26	1.0	31.0	46.0	1.0	20.0	1.0
C	R	27	0.0	27.0	48.0	1.0	24.0	0.0
C	R	28	0.0	28.0	47.0	3.0	21.0	1.0
C	R	29	0.0	24.0	49.0	2.0	24.0	0.0
C	R	30	1.0	27.0	52.0	0.0	20.0	0.0
C	FR	1	0.0	8.0	65.0	5.0	14.0	9.0
C	FR	2	0.0	17.0	58.0	2.0	8.0	15.0
C	FR	3	2.0	15.0	63.0	2.0	11.0	7.0
C	FR	4	0.0	18.0	59.0	2.0	9.0	12.0
C	FR	5	0.0	26.0	56.0	0.0	8.0	10.0
C	FR	6	0.0	19.0	62.0	2.0	5.0	13.0
C	FR	7	0.0	20.0	65.0	5.0	4.0	5.0
C	FR	8	0.0	21.0	62.0	0.0	7.0	10.0
C	FR	9	0.0	22.0	61.0	0.0	9.0	8.0
C	FR	10	0.0	21.0	68.0	2.0	4.0	5.0
C	FR	11	0.0	17.0	74.0	0.0	6.0	4.0
C	FR	12	0.0	20.0	67.0	3.0	7.0	3.0
C	FR	13	2.0	14.0	71.0	2.0	7.0	4.0
C	FR	14	0.0	16.0	62.0	4.0	11.0	7.0
C	FR	15	3.0	17.0	57.0	3.0	7.0	12.0
C	FR	16	0.0	9.0	76.0	2.0	9.0	4.0
C	FR	17	0.0	16.0	69.0	2.0	8.0	5.0
C	FR	18	0.0	29.0	49.0	6.0	4.0	12.0
C	FR	19	0.0	18.0	61.0	2.0	15.0	5.0
C	FR	20	0.0	23.0	65.0	2.0	6.0	5.0
C	FR	21	0.0	24.0	58.0	2.0	7.0	10.0
C	FR	22	0.0	26.0	66.0	2.0	3.0	3.0
C	FR	23	2.0	17.0	67.0	3.0	11.0	2.0
C	FR	24	1.0	21.0	57.0	1.0	12.0	7.0
C	FR	25	0.0	23.0	65.0	0.0	8.0	5.0
C	FR	26	0.0	20.0	62.0	1.0	11.0	7.0
C	FR	27	0.0	14.0	70.0	1.0	10.0	4.0
C	FR	28	0.0	22.0	66.0	4.0	3.0	5.0
C	FR	29	2.0	18.0	60.0	5.0	8.0	8.0
C	FR	30	0.0	15.0	70.0	1.0	9.0	4.0

Table S 18: Proportion of labelled videos falling under each political classification as a user who has watched 30 videos of the "Original class" and then 30 videos of the "New Class" begins to watch the top recommended video in the third stage of the experiment.

Original Class	New Class	Video Number	Far Left	Left	Center	Anti-Woke	Right	Far Right
AW	FL	1	45.0	15.0	15.0	25.0	0.0	0.0
AW	FL	2	41.0	17.0	16.0	24.0	1.0	0.0
AW	FL	3	39.0	19.0	16.0	22.0	4.0	0.0
AW	FL	4	31.0	15.0	21.0	26.0	6.0	0.0
AW	FL	5	34.0	20.0	11.0	30.0	4.0	1.0
AW	FL	6	32.0	15.0	16.0	28.0	8.0	0.0
AW	FL	7	30.0	24.0	15.0	30.0	0.0	0.0
AW	FL	8	36.0	12.0	16.0	30.0	5.0	0.0
AW	FL	9	28.0	20.0	21.0	27.0	3.0	1.0
AW	FL	10	23.0	25.0	14.0	33.0	3.0	2.0
AW	FL	11	32.0	21.0	15.0	28.0	4.0	0.0
AW	FL	12	24.0	14.0	26.0	29.0	7.0	0.0
AW	FL	13	29.0	29.0	14.0	26.0	1.0	0.0
AW	FL	14	27.0	17.0	23.0	28.0	5.0	0.0
AW	FL	15	16.0	24.0	27.0	29.0	3.0	0.0
AW	FL	16	27.0	27.0	18.0	23.0	5.0	0.0
AW	FL	17	26.0	30.0	24.0	20.0	0.0	0.0
AW	FL	18	24.0	30.0	15.0	26.0	5.0	0.0
AW	FL	19	29.0	22.0	24.0	24.0	2.0	0.0
AW	FL	20	33.0	20.0	15.0	26.0	7.0	0.0
AW	FL	21	25.0	25.0	27.0	23.0	0.0	0.0
AW	FL	22	32.0	19.0	22.0	25.0	0.0	2.0
AW	FL	23	23.0	25.0	27.0	23.0	2.0	0.0
AW	FL	24	26.0	19.0	18.0	32.0	4.0	0.0
AW	FL	25	25.0	16.0	16.0	44.0	0.0	0.0
AW	FL	26	17.0	26.0	16.0	37.0	3.0	0.0
AW	FL	27	19.0	26.0	18.0	32.0	4.0	0.0
AW	FL	28	20.0	21.0	17.0	39.0	3.0	0.0
AW	FL	29	24.0	25.0	18.0	32.0	1.0	0.0
AW	FL	30	17.0	21.0	17.0	42.0	3.0	0.0
AW	L	1	0.0	56.0	20.0	19.0	6.0	0.0
AW	L	2	1.0	49.0	15.0	34.0	1.0	0.0
AW	L	3	0.0	67.0	14.0	17.0	3.0	0.0

AW	L	4	3.0	55.0	15.0	21.0	6.0	0.0
AW	L	5	0.0	56.0	18.0	20.0	6.0	0.0
AW	L	6	0.0	54.0	22.0	23.0	1.0	0.0
AW	L	7	0.0	59.0	15.0	20.0	6.0	0.0
AW	L	8	0.0	61.0	15.0	20.0	4.0	0.0
AW	L	9	0.0	62.0	18.0	20.0	0.0	0.0
AW	L	10	3.0	60.0	17.0	19.0	1.0	0.0
AW	L	11	2.0	62.0	10.0	23.0	2.0	0.0
AW	L	12	0.0	56.0	14.0	29.0	1.0	0.0
AW	L	13	1.0	57.0	20.0	16.0	6.0	0.0
AW	L	14	0.0	53.0	21.0	21.0	5.0	0.0
AW	L	15	0.0	53.0	19.0	22.0	5.0	0.0
AW	L	16	1.0	43.0	23.0	28.0	5.0	0.0
AW	L	17	0.0	49.0	21.0	24.0	6.0	0.0
AW	L	18	0.0	51.0	23.0	23.0	3.0	0.0
AW	L	19	1.0	57.0	21.0	18.0	3.0	0.0
AW	L	20	0.0	55.0	16.0	25.0	2.0	1.0
AW	L	21	1.0	44.0	17.0	36.0	0.0	1.0
AW	L	22	0.0	49.0	20.0	28.0	4.0	0.0
AW	L	23	0.0	53.0	14.0	28.0	5.0	0.0
AW	L	24	0.0	43.0	22.0	30.0	5.0	0.0
AW	L	25	1.0	51.0	17.0	27.0	4.0	0.0
AW	L	26	0.0	47.0	21.0	23.0	9.0	0.0
AW	L	27	0.0	53.0	13.0	25.0	9.0	0.0
AW	L	28	0.0	51.0	19.0	24.0	4.0	1.0
AW	L	29	1.0	51.0	20.0	21.0	6.0	0.0
AW	L	30	1.0	48.0	22.0	28.0	1.0	0.0
AW	C	1	1.0	17.0	62.0	17.0	2.0	0.0
AW	C	2	0.0	11.0	70.0	19.0	1.0	0.0
AW	C	3	0.0	12.0	67.0	19.0	2.0	0.0
AW	C	4	0.0	15.0	68.0	16.0	0.0	0.0
AW	C	5	0.0	19.0	65.0	13.0	3.0	0.0
AW	C	6	0.0	11.0	70.0	15.0	5.0	0.0
AW	C	7	0.0	16.0	65.0	16.0	2.0	1.0
AW	C	8	0.0	17.0	65.0	12.0	5.0	1.0
AW	C	9	0.0	16.0	69.0	10.0	5.0	0.0
AW	C	10	0.0	18.0	65.0	16.0	2.0	0.0
AW	C	11	0.0	17.0	61.0	17.0	5.0	0.0
AW	C	12	0.0	15.0	68.0	13.0	3.0	0.0
AW	C	13	0.0	14.0	66.0	18.0	3.0	0.0

AW	C	14	0.0	12.0	64.0	16.0	8.0	0.0
AW	C	15	0.0	14.0	66.0	16.0	4.0	0.0
AW	C	16	0.0	10.0	69.0	16.0	5.0	0.0
AW	C	17	0.0	12.0	70.0	16.0	2.0	0.0
AW	C	18	0.0	14.0	67.0	13.0	6.0	0.0
AW	C	19	0.0	16.0	62.0	19.0	3.0	0.0
AW	C	20	0.0	15.0	65.0	17.0	3.0	0.0
AW	C	21	0.0	17.0	65.0	16.0	2.0	0.0
AW	C	22	0.0	15.0	55.0	25.0	5.0	0.0
AW	C	23	0.0	17.0	58.0	19.0	6.0	0.0
AW	C	24	0.0	14.0	63.0	21.0	1.0	0.0
AW	C	25	1.0	12.0	61.0	19.0	6.0	2.0
AW	C	26	0.0	14.0	59.0	19.0	8.0	0.0
AW	C	27	0.0	12.0	64.0	18.0	6.0	0.0
AW	C	28	0.0	10.0	72.0	11.0	7.0	0.0
AW	C	29	0.0	15.0	62.0	19.0	4.0	0.0
AW	C	30	0.0	12.0	66.0	16.0	5.0	0.0
AW	AW	1	0.0	2.0	6.0	82.0	9.0	1.0
AW	AW	2	0.0	2.0	4.0	82.0	11.0	1.0
AW	AW	3	0.0	1.0	4.0	83.0	11.0	0.0
AW	AW	4	1.0	5.0	4.0	84.0	5.0	0.0
AW	AW	5	0.0	6.0	5.0	79.0	11.0	0.0
AW	AW	6	1.0	7.0	3.0	81.0	8.0	0.0
AW	AW	7	0.0	8.0	5.0	75.0	11.0	0.0
AW	AW	8	1.0	5.0	6.0	85.0	3.0	0.0
AW	AW	9	3.0	5.0	1.0	86.0	5.0	0.0
AW	AW	10	0.0	5.0	6.0	81.0	7.0	0.0
AW	AW	11	2.0	4.0	7.0	80.0	7.0	0.0
AW	AW	12	0.0	8.0	10.0	74.0	8.0	0.0
AW	AW	13	0.0	6.0	5.0	78.0	10.0	0.0
AW	AW	14	0.0	3.0	3.0	86.0	8.0	0.0
AW	AW	15	0.0	6.0	4.0	76.0	14.0	0.0
AW	AW	16	0.0	5.0	1.0	88.0	6.0	0.0
AW	AW	17	0.0	10.0	6.0	71.0	13.0	0.0
AW	AW	18	0.0	8.0	11.0	72.0	8.0	1.0
AW	AW	19	0.0	3.0	5.0	86.0	6.0	0.0
AW	AW	20	2.0	5.0	10.0	78.0	6.0	0.0
AW	AW	21	0.0	3.0	0.0	89.0	8.0	0.0
AW	AW	22	0.0	6.0	9.0	83.0	1.0	0.0
AW	AW	23	0.0	5.0	2.0	81.0	12.0	0.0

AW	AW	24	0.0	12.0	8.0	70.0	10.0	0.0
AW	AW	25	0.0	6.0	11.0	78.0	5.0	0.0
AW	AW	26	0.0	11.0	10.0	73.0	6.0	0.0
AW	AW	27	0.0	5.0	8.0	81.0	5.0	2.0
AW	AW	28	0.0	6.0	11.0	70.0	13.0	0.0
AW	AW	29	2.0	5.0	7.0	79.0	7.0	0.0
AW	AW	30	0.0	7.0	8.0	73.0	10.0	2.0
AW	R	1	0.0	16.0	22.0	34.0	28.0	0.0
AW	R	2	0.0	14.0	26.0	28.0	31.0	1.0
AW	R	3	0.0	16.0	25.0	22.0	34.0	2.0
AW	R	4	0.0	9.0	30.0	23.0	36.0	1.0
AW	R	5	0.0	15.0	31.0	32.0	21.0	0.0
AW	R	6	0.0	18.0	27.0	24.0	32.0	0.0
AW	R	7	0.0	13.0	34.0	24.0	27.0	2.0
AW	R	8	0.0	11.0	43.0	20.0	26.0	0.0
AW	R	9	0.0	24.0	29.0	29.0	17.0	1.0
AW	R	10	0.0	14.0	26.0	32.0	27.0	0.0
AW	R	11	1.0	19.0	37.0	25.0	17.0	0.0
AW	R	12	0.0	18.0	30.0	29.0	22.0	1.0
AW	R	13	0.0	12.0	36.0	29.0	23.0	0.0
AW	R	14	0.0	11.0	44.0	20.0	23.0	1.0
AW	R	15	3.0	13.0	38.0	29.0	17.0	0.0
AW	R	16	0.0	20.0	37.0	26.0	18.0	0.0
AW	R	17	1.0	12.0	27.0	37.0	22.0	0.0
AW	R	18	0.0	19.0	27.0	29.0	25.0	0.0
AW	R	19	0.0	14.0	33.0	36.0	17.0	0.0
AW	R	20	0.0	12.0	37.0	32.0	19.0	0.0
AW	R	21	0.0	18.0	36.0	25.0	21.0	0.0
AW	R	22	0.0	27.0	29.0	24.0	18.0	1.0
AW	R	23	0.0	19.0	35.0	28.0	18.0	0.0
AW	R	24	0.0	12.0	32.0	29.0	27.0	0.0
AW	R	25	0.0	14.0	29.0	30.0	26.0	1.0
AW	R	26	0.0	22.0	24.0	34.0	19.0	1.0
AW	R	27	0.0	9.0	36.0	34.0	21.0	0.0
AW	R	28	0.0	12.0	35.0	30.0	22.0	0.0
AW	R	29	0.0	12.0	36.0	30.0	23.0	0.0
AW	R	30	0.0	15.0	26.0	29.0	28.0	1.0
AW	FR	1	0.0	1.0	8.0	45.0	16.0	29.0
AW	FR	2	0.0	7.0	3.0	54.0	15.0	21.0
AW	FR	3	0.0	4.0	2.0	69.0	13.0	12.0

AW	FR	4	0.0	0.0	9.0	57.0	15.0	19.0
AW	FR	5	0.0	10.0	10.0	50.0	10.0	19.0
AW	FR	6	2.0	6.0	17.0	47.0	17.0	11.0
AW	FR	7	0.0	2.0	18.0	53.0	8.0	18.0
AW	FR	8	0.0	5.0	5.0	62.0	18.0	10.0
AW	FR	9	0.0	9.0	13.0	57.0	13.0	9.0
AW	FR	10	0.0	7.0	0.0	56.0	16.0	21.0
AW	FR	11	0.0	9.0	6.0	45.0	28.0	13.0
AW	FR	12	0.0	7.0	7.0	53.0	18.0	16.0
AW	FR	13	0.0	10.0	8.0	49.0	24.0	8.0
AW	FR	14	0.0	12.0	14.0	45.0	12.0	16.0
AW	FR	15	0.0	13.0	9.0	44.0	20.0	13.0
AW	FR	16	4.0	10.0	6.0	44.0	21.0	15.0
AW	FR	17	0.0	9.0	6.0	44.0	18.0	24.0
AW	FR	18	0.0	5.0	10.0	59.0	13.0	13.0
AW	FR	19	2.0	13.0	13.0	49.0	9.0	13.0
AW	FR	20	0.0	9.0	12.0	44.0	19.0	16.0
AW	FR	21	3.0	5.0	13.0	59.0	8.0	13.0
AW	FR	22	0.0	18.0	3.0	51.0	8.0	21.0
AW	FR	23	3.0	6.0	14.0	60.0	9.0	9.0
AW	FR	24	4.0	9.0	11.0	46.0	7.0	24.0
AW	FR	25	0.0	11.0	13.0	50.0	16.0	11.0
AW	FR	26	4.0	4.0	11.0	71.0	7.0	4.0
AW	FR	27	0.0	5.0	8.0	59.0	11.0	16.0
AW	FR	28	0.0	11.0	9.0	52.0	11.0	16.0
AW	FR	29	4.0	8.0	0.0	61.0	10.0	18.0
AW	FR	30	5.0	9.0	7.0	67.0	7.0	5.0

Table S 19: Proportion of labelled videos falling under each political classification as a user who has watched 30 videos of the "Original class" and then 30 videos of the "New Class" begins to watch the top recommended video in the third stage of the experiment.

**Original Class**	**New Class**	**Video Number**	**Far Left**	**Left**	**Center**	**Anti-Woke**	**Right**	**Far Right**
R	FL	1	43.0	14.0	11.0	5.0	28.0	0.0
R	FL	2	30.0	12.0	20.0	7.0	31.0	0.0
R	FL	3	36.0	17.0	20.0	6.0	20.0	2.0
R	FL	4	26.0	18.0	15.0	4.0	37.0	0.0
R	FL	5	38.0	15.0	25.0	5.0	16.0	0.0
R	FL	6	38.0	19.0	16.0	3.0	24.0	0.0

R	FL	7	38.0	19.0	19.0	5.0	19.0	0.0
R	FL	8	36.0	12.0	24.0	5.0	23.0	0.0
R	FL	9	37.0	19.0	17.0	2.0	26.0	0.0
R	FL	10	32.0	13.0	18.0	6.0	31.0	0.0
R	FL	11	33.0	25.0	22.0	6.0	14.0	0.0
R	FL	12	29.0	21.0	23.0	2.0	24.0	2.0
R	FL	13	37.0	15.0	17.0	0.0	31.0	0.0
R	FL	14	33.0	19.0	27.0	4.0	16.0	0.0
R	FL	15	26.0	30.0	27.0	5.0	11.0	0.0
R	FL	16	22.0	26.0	32.0	1.0	18.0	0.0
R	FL	17	28.0	15.0	43.0	3.0	9.0	2.0
R	FL	18	34.0	23.0	19.0	3.0	20.0	0.0
R	FL	19	25.0	25.0	27.0	3.0	19.0	0.0
R	FL	20	27.0	23.0	31.0	5.0	14.0	0.0
R	FL	21	24.0	25.0	27.0	7.0	16.0	0.0
R	FL	22	25.0	19.0	31.0	7.0	16.0	0.0
R	FL	23	30.0	19.0	32.0	4.0	14.0	0.0
R	FL	24	22.0	16.0	34.0	0.0	24.0	3.0
R	FL	25	21.0	22.0	33.0	4.0	19.0	0.0
R	FL	26	27.0	19.0	24.0	3.0	26.0	0.0
R	FL	27	20.0	30.0	30.0	4.0	14.0	0.0
R	FL	28	25.0	28.0	25.0	3.0	17.0	2.0
R	FL	29	20.0	27.0	29.0	5.0	19.0	0.0
R	FL	30	19.0	23.0	25.0	10.0	23.0	0.0
R	L	1	0.0	58.0	27.0	0.0	15.0	0.0
R	L	2	0.0	60.0	28.0	0.0	13.0	0.0
R	L	3	1.0	55.0	25.0	1.0	18.0	0.0
R	L	4	1.0	46.0	30.0	8.0	15.0	0.0
R	L	5	0.0	41.0	25.0	17.0	16.0	0.0
R	L	6	0.0	39.0	29.0	15.0	17.0	0.0
R	L	7	0.0	53.0	33.0	3.0	11.0	0.0
R	L	8	0.0	53.0	33.0	0.0	14.0	0.0
R	L	9	0.0	45.0	42.0	2.0	10.0	0.0
R	L	10	1.0	52.0	27.0	0.0	17.0	2.0
R	L	11	1.0	57.0	30.0	3.0	9.0	0.0
R	L	12	1.0	51.0	31.0	0.0	18.0	0.0
R	L	13	0.0	56.0	31.0	0.0	12.0	0.0
R	L	14	0.0	44.0	39.0	0.0	17.0	0.0
R	L	15	0.0	50.0	36.0	1.0	12.0	0.0
R	L	16	0.0	48.0	37.0	1.0	13.0	1.0

R	L	17	1.0	45.0	33.0	1.0	20.0	0.0
R	L	18	0.0	48.0	33.0	1.0	18.0	0.0
R	L	19	0.0	49.0	35.0	0.0	17.0	0.0
R	L	20	0.0	46.0	38.0	1.0	13.0	1.0
R	L	21	0.0	47.0	35.0	0.0	18.0	0.0
R	L	22	0.0	49.0	40.0	0.0	11.0	0.0
R	L	23	0.0	57.0	32.0	1.0	10.0	0.0
R	L	24	0.0	50.0	37.0	1.0	12.0	0.0
R	L	25	0.0	49.0	39.0	2.0	9.0	0.0
R	L	26	0.0	50.0	41.0	0.0	9.0	0.0
R	L	27	0.0	39.0	45.0	1.0	15.0	0.0
R	L	28	0.0	45.0	39.0	1.0	15.0	0.0
R	L	29	0.0	48.0	32.0	2.0	18.0	0.0
R	L	30	0.0	45.0	31.0	3.0	20.0	1.0
R	C	1	0.0	16.0	63.0	3.0	16.0	1.0
R	C	2	0.0	12.0	76.0	2.0	10.0	0.0
R	C	3	0.0	15.0	64.0	5.0	13.0	4.0
R	C	4	0.0	11.0	67.0	3.0	17.0	1.0
R	C	5	0.0	15.0	71.0	1.0	12.0	1.0
R	C	6	0.0	13.0	73.0	3.0	11.0	0.0
R	C	7	0.0	17.0	60.0	0.0	22.0	1.0
R	C	8	0.0	12.0	66.0	2.0	19.0	1.0
R	C	9	0.0	18.0	62.0	0.0	20.0	0.0
R	C	10	0.0	16.0	68.0	2.0	14.0	0.0
R	C	11	0.0	20.0	65.0	0.0	14.0	0.0
R	C	12	0.0	17.0	62.0	5.0	16.0	0.0
R	C	13	0.0	14.0	65.0	5.0	15.0	0.0
R	C	14	0.0	11.0	73.0	1.0	14.0	0.0
R	C	15	0.0	19.0	66.0	1.0	12.0	1.0
R	C	16	0.0	18.0	57.0	3.0	21.0	1.0
R	C	17	0.0	18.0	55.0	4.0	22.0	0.0
R	C	18	0.0	21.0	60.0	3.0	17.0	0.0
R	C	19	0.0	22.0	65.0	2.0	10.0	0.0
R	C	20	0.0	15.0	66.0	2.0	17.0	0.0
R	C	21	0.0	15.0	63.0	2.0	16.0	4.0
R	C	22	0.0	12.0	63.0	3.0	20.0	1.0
R	C	23	0.0	14.0	68.0	6.0	11.0	1.0
R	C	24	0.0	19.0	62.0	4.0	15.0	0.0
R	C	25	0.0	17.0	58.0	4.0	21.0	0.0
R	C	26	0.0	19.0	57.0	4.0	19.0	0.0

R	C	27	0.0	15.0	66.0	2.0	15.0	1.0
R	C	28	0.0	15.0	63.0	2.0	20.0	0.0
R	C	29	0.0	14.0	66.0	4.0	16.0	0.0
R	C	30	0.0	18.0	57.0	4.0	20.0	1.0
R	AW	1	1.0	9.0	11.0	57.0	21.0	1.0
R	AW	2	0.0	11.0	8.0	59.0	22.0	0.0
R	AW	3	0.0	9.0	10.0	49.0	30.0	1.0
R	AW	4	0.0	16.0	11.0	49.0	22.0	1.0
R	AW	5	1.0	12.0	15.0	49.0	21.0	1.0
R	AW	6	1.0	12.0	16.0	49.0	20.0	1.0
R	AW	7	0.0	11.0	17.0	58.0	14.0	0.0
R	AW	8	2.0	13.0	13.0	41.0	29.0	0.0
R	AW	9	1.0	7.0	18.0	41.0	29.0	2.0
R	AW	10	1.0	10.0	18.0	49.0	20.0	1.0
R	AW	11	0.0	11.0	10.0	56.0	22.0	1.0
R	AW	12	1.0	13.0	22.0	51.0	12.0	1.0
R	AW	13	4.0	11.0	21.0	44.0	20.0	0.0
R	AW	14	0.0	12.0	15.0	52.0	21.0	0.0
R	AW	15	0.0	13.0	27.0	47.0	14.0	0.0
R	AW	16	1.0	10.0	22.0	51.0	15.0	0.0
R	AW	17	0.0	11.0	25.0	47.0	16.0	1.0
R	AW	18	0.0	12.0	16.0	48.0	23.0	1.0
R	AW	19	0.0	12.0	22.0	43.0	21.0	1.0
R	AW	20	0.0	10.0	17.0	49.0	24.0	0.0
R	AW	21	1.0	10.0	19.0	46.0	20.0	3.0
R	AW	22	0.0	8.0	18.0	47.0	26.0	2.0
R	AW	23	0.0	13.0	22.0	48.0	17.0	0.0
R	AW	24	0.0	7.0	23.0	45.0	23.0	2.0
R	AW	25	0.0	13.0	17.0	45.0	22.0	3.0
R	AW	26	0.0	15.0	16.0	43.0	24.0	2.0
R	AW	27	0.0	10.0	19.0	51.0	18.0	1.0
R	AW	28	0.0	9.0	15.0	57.0	19.0	0.0
R	AW	29	2.0	10.0	18.0	41.0	27.0	2.0
R	AW	30	3.0	10.0	23.0	49.0	14.0	1.0
R	R	1	0.0	8.0	36.0	0.0	55.0	1.0
R	R	2	0.0	11.0	29.0	2.0	58.0	0.0
R	R	3	0.0	15.0	27.0	1.0	56.0	1.0
R	R	4	0.0	15.0	23.0	1.0	57.0	3.0
R	R	5	0.0	20.0	22.0	0.0	58.0	0.0
R	R	6	0.0	8.0	27.0	0.0	63.0	1.0

R	R	7	1.0	10.0	35.0	2.0	51.0	1.0
R	R	8	0.0	11.0	24.0	1.0	63.0	1.0
R	R	9	0.0	16.0	27.0	2.0	52.0	2.0
R	R	10	0.0	9.0	38.0	3.0	50.0	0.0
R	R	11	1.0	10.0	41.0	2.0	46.0	0.0
R	R	12	0.0	12.0	35.0	4.0	49.0	0.0
R	R	13	1.0	13.0	34.0	0.0	51.0	1.0
R	R	14	1.0	14.0	37.0	3.0	45.0	0.0
R	R	15	0.0	13.0	33.0	1.0	51.0	1.0
R	R	16	0.0	11.0	33.0	1.0	52.0	2.0
R	R	17	0.0	10.0	32.0	0.0	57.0	1.0
R	R	18	0.0	12.0	28.0	0.0	59.0	1.0
R	R	19	1.0	10.0	29.0	0.0	58.0	1.0
R	R	20	0.0	9.0	30.0	1.0	60.0	0.0
R	R	21	0.0	12.0	30.0	0.0	54.0	4.0
R	R	22	0.0	10.0	29.0	3.0	59.0	0.0
R	R	23	0.0	17.0	26.0	1.0	55.0	1.0
R	R	24	1.0	15.0	36.0	0.0	47.0	1.0
R	R	25	0.0	10.0	37.0	1.0	51.0	0.0
R	R	26	1.0	11.0	26.0	3.0	58.0	1.0
R	R	27	0.0	19.0	19.0	1.0	58.0	3.0
R	R	28	0.0	13.0	29.0	1.0	57.0	0.0
R	R	29	0.0	9.0	39.0	1.0	49.0	1.0
R	R	30	1.0	15.0	27.0	2.0	54.0	0.0
R	FR	1	0.0	13.0	31.0	5.0	31.0	21.0
R	FR	2	0.0	14.0	27.0	8.0	37.0	14.0
R	FR	3	0.0	12.0	23.0	3.0	42.0	20.0
R	FR	4	0.0	19.0	34.0	6.0	31.0	9.0
R	FR	5	0.0	16.0	27.0	1.0	45.0	10.0
R	FR	6	0.0	14.0	32.0	0.0	35.0	18.0
R	FR	7	1.0	24.0	28.0	0.0	36.0	10.0
R	FR	8	2.0	28.0	27.0	3.0	30.0	10.0
R	FR	9	0.0	16.0	26.0	3.0	39.0	15.0
R	FR	10	1.0	36.0	21.0	3.0	31.0	8.0
R	FR	11	0.0	28.0	25.0	3.0	36.0	8.0
R	FR	12	1.0	28.0	29.0	0.0	31.0	11.0
R	FR	13	2.0	18.0	43.0	2.0	22.0	13.0
R	FR	14	1.0	22.0	35.0	1.0	25.0	15.0
R	FR	15	0.0	23.0	25.0	5.0	38.0	9.0
R	FR	16	0.0	30.0	31.0	4.0	24.0	10.0

R	FR	17	0.0	22.0	25.0	5.0	39.0	8.0
R	FR	18	0.0	25.0	32.0	0.0	35.0	8.0
R	FR	19	0.0	26.0	24.0	3.0	33.0	14.0
R	FR	20	0.0	22.0	27.0	5.0	38.0	8.0
R	FR	21	0.0	29.0	30.0	2.0	30.0	9.0
R	FR	22	2.0	35.0	27.0	2.0	26.0	8.0
R	FR	23	0.0	25.0	26.0	7.0	30.0	12.0
R	FR	24	1.0	37.0	28.0	4.0	28.0	1.0
R	FR	25	0.0	27.0	25.0	8.0	32.0	7.0
R	FR	26	0.0	31.0	24.0	3.0	28.0	14.0
R	FR	27	0.0	31.0	28.0	3.0	27.0	11.0
R	FR	28	0.0	30.0	34.0	0.0	25.0	10.0
R	FR	29	1.0	31.0	22.0	3.0	27.0	15.0
R	FR	30	0.0	33.0	22.0	9.0	26.0	10.0

Table S 20: Proportion of labelled videos falling under each political classification as a user who has watched 30 videos of the “Original class” and then 30 videos of the “New Class” begins to watch the top recommended video in the third stage of the experiment.

**Original Class**	**New Class**	**Video Number**	**Far Left**	**Left**	**Center**	**Anti-Woke**	**Right**	**Far Right**
FR	FL	1	58.0	10.0	11.0	6.0	9.0	6.0
FR	FL	2	60.0	9.0	9.0	10.0	7.0	6.0
FR	FL	3	55.0	18.0	6.0	6.0	5.0	9.0
FR	FL	4	52.0	11.0	15.0	14.0	1.0	6.0
FR	FL	5	43.0	18.0	12.0	8.0	8.0	11.0
FR	FL	6	49.0	27.0	8.0	7.0	5.0	3.0
FR	FL	7	55.0	16.0	11.0	4.0	5.0	9.0
FR	FL	8	49.0	16.0	17.0	5.0	8.0	5.0
FR	FL	9	51.0	16.0	7.0	6.0	7.0	13.0
FR	FL	10	48.0	14.0	12.0	9.0	9.0	9.0
FR	FL	11	33.0	17.0	14.0	7.0	19.0	9.0
FR	FL	12	39.0	35.0	9.0	6.0	9.0	2.0
FR	FL	13	49.0	19.0	15.0	1.0	4.0	10.0
FR	FL	14	33.0	30.0	12.0	4.0	12.0	9.0
FR	FL	15	48.0	27.0	15.0	2.0	6.0	2.0
FR	FL	16	41.0	19.0	11.0	5.0	12.0	12.0
FR	FL	17	50.0	17.0	11.0	9.0	6.0	7.0
FR	FL	18	41.0	19.0	9.0	7.0	16.0	9.0
FR	FL	19	43.0	26.0	6.0	13.0	6.0	7.0

FR	FL	20	37.0	22.0	17.0	7.0	13.0	5.0
FR	FL	21	46.0	27.0	10.0	0.0	8.0	8.0
FR	FL	22	39.0	22.0	8.0	8.0	14.0	9.0
FR	FL	23	41.0	21.0	19.0	3.0	7.0	9.0
FR	FL	24	51.0	16.0	9.0	4.0	16.0	3.0
FR	FL	25	41.0	17.0	21.0	5.0	7.0	9.0
FR	FL	26	48.0	18.0	5.0	4.0	11.0	14.0
FR	FL	27	46.0	21.0	8.0	8.0	4.0	11.0
FR	FL	28	52.0	18.0	10.0	7.0	7.0	7.0
FR	FL	29	55.0	22.0	6.0	4.0	6.0	7.0
FR	FL	30	43.0	24.0	12.0	5.0	5.0	10.0
FR	L	1	1.0	65.0	20.0	1.0	5.0	8.0
FR	L	2	0.0	62.0	30.0	1.0	3.0	3.0
FR	L	3	0.0	63.0	19.0	1.0	10.0	6.0
FR	L	4	0.0	63.0	32.0	1.0	1.0	2.0
FR	L	5	0.0	71.0	24.0	0.0	1.0	3.0
FR	L	6	1.0	60.0	28.0	1.0	7.0	3.0
FR	L	7	0.0	59.0	30.0	0.0	3.0	8.0
FR	L	8	1.0	58.0	29.0	2.0	4.0	6.0
FR	L	9	0.0	64.0	20.0	1.0	5.0	10.0
FR	L	10	0.0	68.0	18.0	1.0	5.0	8.0
FR	L	11	0.0	62.0	29.0	0.0	3.0	6.0
FR	L	12	1.0	65.0	24.0	1.0	4.0	4.0
FR	L	13	0.0	64.0	23.0	0.0	3.0	9.0
FR	L	14	1.0	63.0	25.0	3.0	3.0	5.0
FR	L	15	0.0	63.0	24.0	0.0	3.0	10.0
FR	L	16	0.0	53.0	34.0	0.0	6.0	7.0
FR	L	17	1.0	67.0	18.0	3.0	5.0	5.0
FR	L	18	0.0	65.0	23.0	2.0	5.0	5.0
FR	L	19	0.0	56.0	34.0	0.0	3.0	7.0
FR	L	20	0.0	57.0	32.0	1.0	0.0	10.0
FR	L	21	1.0	56.0	35.0	0.0	4.0	4.0
FR	L	22	1.0	46.0	41.0	2.0	2.0	6.0
FR	L	23	1.0	54.0	35.0	2.0	5.0	2.0
FR	L	24	0.0	58.0	35.0	0.0	3.0	4.0
FR	L	25	4.0	56.0	31.0	1.0	2.0	6.0
FR	L	26	2.0	52.0	38.0	0.0	2.0	6.0
FR	L	27	1.0	47.0	35.0	0.0	9.0	7.0
FR	L	28	0.0	55.0	30.0	2.0	6.0	6.0
FR	L	29	0.0	63.0	25.0	1.0	7.0	4.0

FR	L	30	0.0	60.0	28.0	3.0	2.0	6.0
FR	C	1	0.0	17.0	68.0	1.0	9.0	5.0
FR	C	2	0.0	14.0	80.0	2.0	5.0	0.0
FR	C	3	1.0	17.0	74.0	3.0	5.0	0.0
FR	C	4	0.0	18.0	75.0	3.0	3.0	1.0
FR	C	5	0.0	10.0	78.0	1.0	7.0	3.0
FR	C	6	0.0	16.0	78.0	2.0	3.0	0.0
FR	C	7	0.0	11.0	74.0	4.0	6.0	4.0
FR	C	8	1.0	11.0	75.0	3.0	6.0	3.0
FR	C	9	0.0	24.0	65.0	2.0	3.0	7.0
FR	C	10	0.0	16.0	69.0	1.0	9.0	6.0
FR	C	11	0.0	19.0	75.0	0.0	4.0	2.0
FR	C	12	0.0	15.0	71.0	1.0	4.0	9.0
FR	C	13	0.0	12.0	72.0	0.0	9.0	7.0
FR	C	14	0.0	14.0	70.0	1.0	12.0	2.0
FR	C	15	1.0	24.0	65.0	1.0	3.0	6.0
FR	C	16	0.0	21.0	63.0	1.0	9.0	5.0
FR	C	17	0.0	23.0	71.0	0.0	4.0	2.0
FR	C	18	0.0	17.0	73.0	1.0	3.0	5.0
FR	C	19	0.0	18.0	68.0	5.0	4.0	5.0
FR	C	20	2.0	16.0	71.0	1.0	5.0	4.0
FR	C	21	0.0	21.0	69.0	1.0	6.0	3.0
FR	C	22	0.0	20.0	74.0	1.0	2.0	2.0
FR	C	23	0.0	23.0	69.0	1.0	5.0	2.0
FR	C	24	0.0	25.0	70.0	0.0	1.0	4.0
FR	C	25	0.0	25.0	68.0	1.0	4.0	2.0
FR	C	26	1.0	24.0	62.0	2.0	7.0	2.0
FR	C	27	0.0	21.0	62.0	2.0	12.0	2.0
FR	C	28	0.0	23.0	66.0	5.0	5.0	2.0
FR	C	29	0.0	19.0	68.0	4.0	9.0	1.0
FR	C	30	0.0	24.0	65.0	1.0	6.0	4.0
FR	AW	1	2.0	4.0	7.0	56.0	18.0	13.0
FR	AW	2	0.0	13.0	7.0	57.0	12.0	10.0
FR	AW	3	0.0	10.0	7.0	57.0	17.0	9.0
FR	AW	4	2.0	3.0	2.0	70.0	16.0	8.0
FR	AW	5	0.0	5.0	6.0	61.0	16.0	11.0
FR	AW	6	0.0	1.0	5.0	72.0	12.0	9.0
FR	AW	7	1.0	8.0	7.0	58.0	14.0	12.0
FR	AW	8	0.0	5.0	8.0	69.0	13.0	5.0
FR	AW	9	0.0	2.0	6.0	64.0	14.0	14.0

FR	AW	10	0.0	8.0	8.0	69.0	12.0	3.0
FR	AW	11	0.0	8.0	6.0	67.0	12.0	8.0
FR	AW	12	1.0	6.0	15.0	51.0	18.0	8.0
FR	AW	13	0.0	4.0	8.0	58.0	19.0	10.0
FR	AW	14	0.0	10.0	10.0	55.0	17.0	8.0
FR	AW	15	1.0	7.0	17.0	48.0	12.0	15.0
FR	AW	16	0.0	7.0	12.0	68.0	9.0	4.0
FR	AW	17	0.0	14.0	14.0	57.0	9.0	6.0
FR	AW	18	0.0	16.0	7.0	54.0	18.0	5.0
FR	AW	19	0.0	11.0	8.0	59.0	17.0	6.0
FR	AW	20	0.0	5.0	12.0	68.0	10.0	4.0
FR	AW	21	1.0	12.0	16.0	54.0	8.0	9.0
FR	AW	22	0.0	13.0	10.0	57.0	14.0	6.0
FR	AW	23	0.0	12.0	19.0	46.0	12.0	11.0
FR	AW	24	0.0	11.0	12.0	59.0	14.0	4.0
FR	AW	25	0.0	6.0	8.0	71.0	11.0	5.0
FR	AW	26	2.0	12.0	3.0	59.0	17.0	7.0
FR	AW	27	0.0	12.0	9.0	59.0	13.0	7.0
FR	AW	28	0.0	13.0	7.0	54.0	14.0	11.0
FR	AW	29	0.0	10.0	15.0	55.0	16.0	3.0
FR	AW	30	0.0	9.0	12.0	55.0	14.0	9.0
FR	R	1	0.0	17.0	26.0	3.0	38.0	17.0
FR	R	2	1.0	19.0	28.0	1.0	37.0	12.0
FR	R	3	1.0	20.0	25.0	4.0	42.0	9.0
FR	R	4	0.0	16.0	25.0	8.0	34.0	17.0
FR	R	5	0.0	19.0	27.0	4.0	37.0	14.0
FR	R	6	0.0	22.0	31.0	0.0	34.0	12.0
FR	R	7	0.0	16.0	34.0	2.0	39.0	8.0
FR	R	8	1.0	23.0	33.0	1.0	31.0	10.0
FR	R	9	0.0	18.0	35.0	2.0	27.0	18.0
FR	R	10	0.0	24.0	26.0	1.0	34.0	15.0
FR	R	11	0.0	13.0	39.0	4.0	27.0	16.0
FR	R	12	3.0	17.0	32.0	1.0	37.0	11.0
FR	R	13	4.0	19.0	31.0	2.0	29.0	15.0
FR	R	14	5.0	27.0	32.0	1.0	22.0	12.0
FR	R	15	3.0	20.0	29.0	4.0	32.0	12.0
FR	R	16	1.0	24.0	25.0	6.0	32.0	11.0
FR	R	17	1.0	12.0	38.0	5.0	31.0	13.0
FR	R	18	2.0	17.0	35.0	0.0	35.0	12.0
FR	R	19	0.0	20.0	27.0	6.0	33.0	14.0

FR	R	20	0.0	18.0	29.0	0.0	34.0	19.0
FR	R	21	0.0	20.0	34.0	3.0	32.0	11.0
FR	R	22	0.0	17.0	35.0	3.0	26.0	20.0
FR	R	23	0.0	24.0	22.0	6.0	33.0	15.0
FR	R	24	0.0	20.0	35.0	2.0	28.0	15.0
FR	R	25	1.0	13.0	37.0	1.0	34.0	12.0
FR	R	26	0.0	21.0	34.0	1.0	36.0	7.0
FR	R	27	1.0	15.0	38.0	1.0	34.0	10.0
FR	R	28	0.0	21.0	32.0	4.0	31.0	12.0
FR	R	29	1.0	13.0	36.0	3.0	30.0	16.0
FR	R	30	0.0	19.0	34.0	0.0	37.0	10.0
FR	FR	1	2.0	5.0	18.0	5.0	28.0	42.0
FR	FR	2	0.0	3.0	12.0	9.0	18.0	58.0
FR	FR	3	0.0	4.0	4.0	4.0	13.0	74.0
FR	FR	4	0.0	0.0	21.0	0.0	16.0	63.0
FR	FR	5	0.0	4.0	4.0	0.0	19.0	73.0
FR	FR	6	0.0	4.0	12.0	4.0	35.0	46.0
FR	FR	7	7.0	0.0	7.0	7.0	14.0	64.0
FR	FR	8	0.0	7.0	27.0	0.0	20.0	47.0
FR	FR	9	0.0	15.0	15.0	5.0	15.0	50.0
FR	FR	10	0.0	0.0	6.0	6.0	24.0	65.0
FR	FR	11	0.0	17.0	6.0	6.0	17.0	56.0
FR	FR	12	0.0	27.0	14.0	9.0	14.0	36.0
FR	FR	13	5.0	0.0	16.0	0.0	16.0	63.0
FR	FR	14	6.0	6.0	24.0	6.0	6.0	53.0
FR	FR	15	0.0	11.0	21.0	0.0	16.0	53.0
FR	FR	16	0.0	5.0	20.0	0.0	10.0	65.0
FR	FR	17	0.0	0.0	11.0	4.0	4.0	81.0
FR	FR	18	0.0	14.0	7.0	0.0	21.0	57.0
FR	FR	19	4.0	0.0	8.0	0.0	38.0	50.0
FR	FR	20	0.0	12.0	12.0	0.0	19.0	56.0
FR	FR	21	0.0	5.0	5.0	5.0	16.0	68.0
FR	FR	22	0.0	10.0	14.0	10.0	14.0	52.0
FR	FR	23	0.0	12.0	4.0	4.0	21.0	58.0
FR	FR	24	0.0	15.0	10.0	5.0	5.0	65.0
FR	FR	25	0.0	10.0	14.0	0.0	14.0	62.0
FR	FR	26	0.0	0.0	25.0	0.0	12.0	62.0
FR	FR	27	0.0	14.0	5.0	0.0	5.0	76.0
FR	FR	28	0.0	14.0	14.0	5.0	9.0	59.0
FR	FR	29	0.0	6.0	6.0	12.0	24.0	53.0

FR	FR	30	0.0	7.0	14.0	7.0	10.0	62.0

Table S 21: Proportion of labelled videos falling under each political classification as a user who has watched 30 videos of the “Original class” and then 30 videos of the “New Class” begins to watch the top recommended video in the third stage of the experiment.

Supplementary Figures

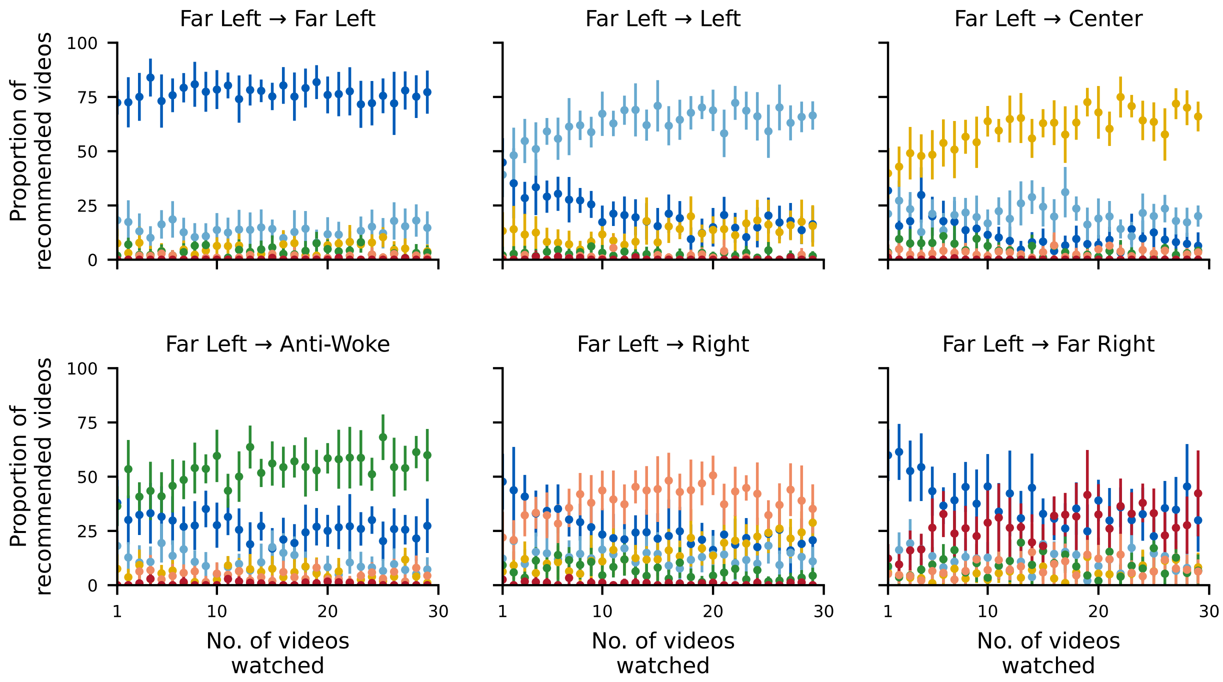

Figure S1: **Transitioning from a pure Far Left persona to different political personas.** Means and 95% confidence intervals of the proportion of different political classes for videos recommended to a user as they watch videos of the target political class. The title of each plot designates the original class of the user, with the arrow pointing to the target class.

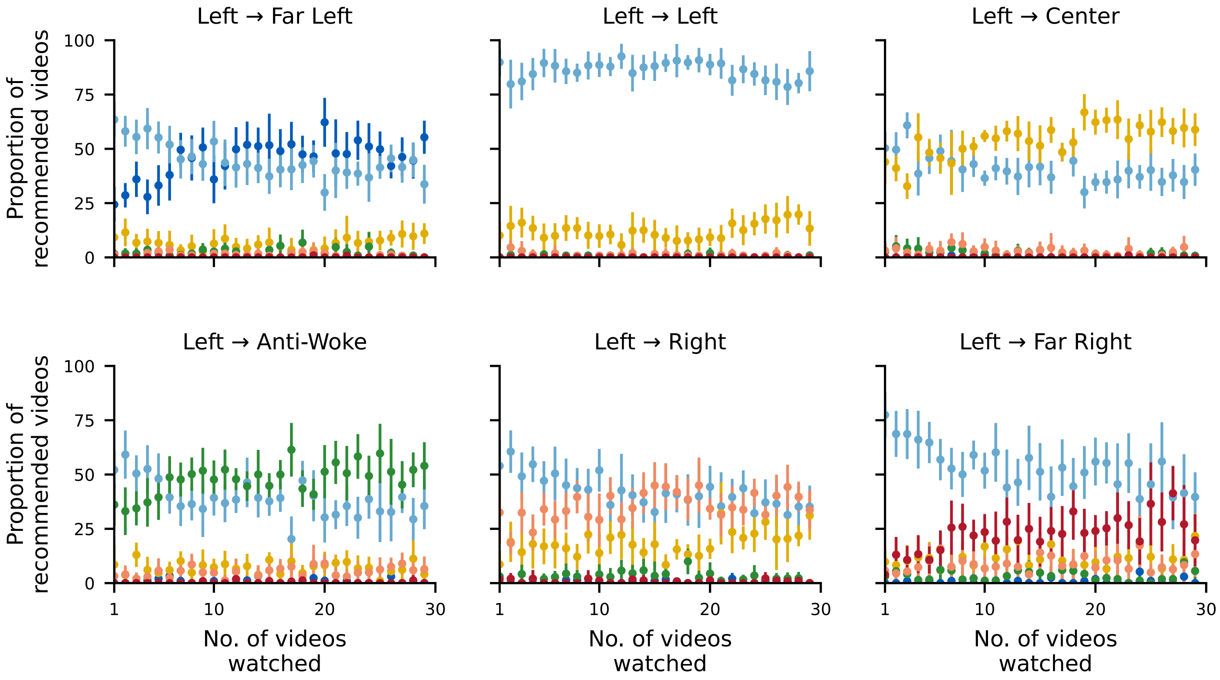

Figure S2: **Transitioning from a pure Left persona to different political personas.** Means and 95% confidence intervals of the proportion of different political classes for videos recommended to a user as they watch videos of the target political class. The title of each plot designates the original class of the user, with the arrow pointing to the target class.

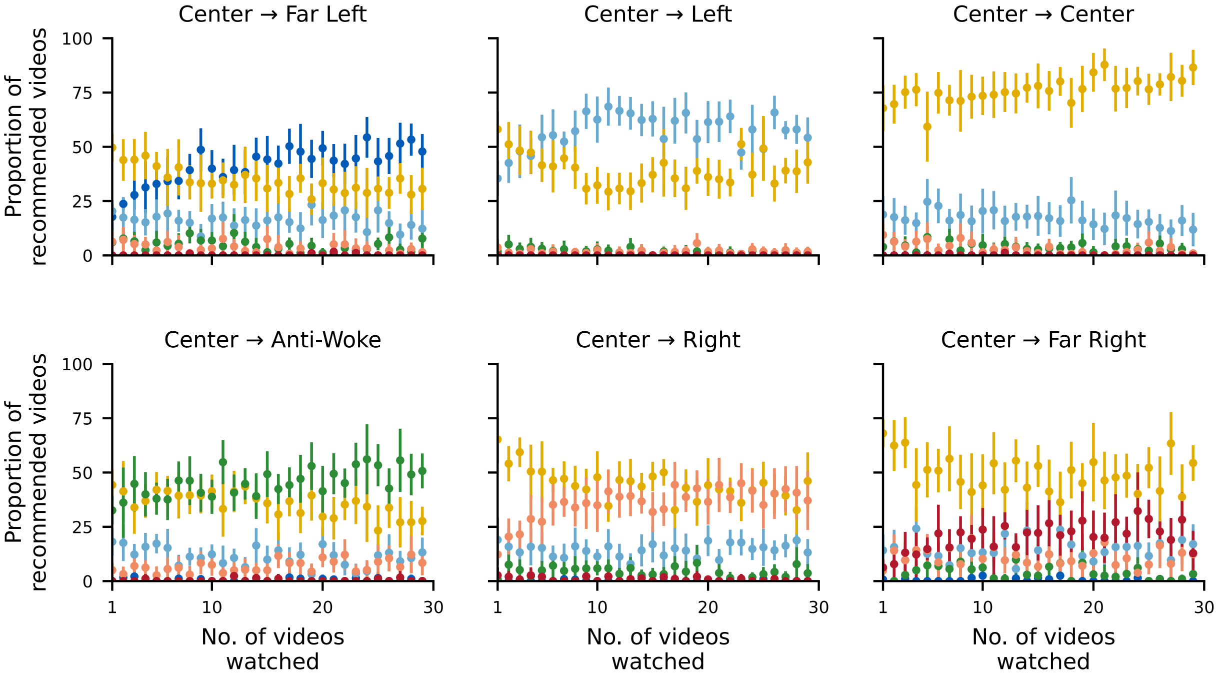

Figure S3: **Transitioning from a pure Center persona to different political personas.** Means and 95% confidence intervals of the proportion of different political classes for videos recommended to a user as they watch videos of the target political class. The title of each plot designates the original class of the user, with the arrow pointing to the target class.

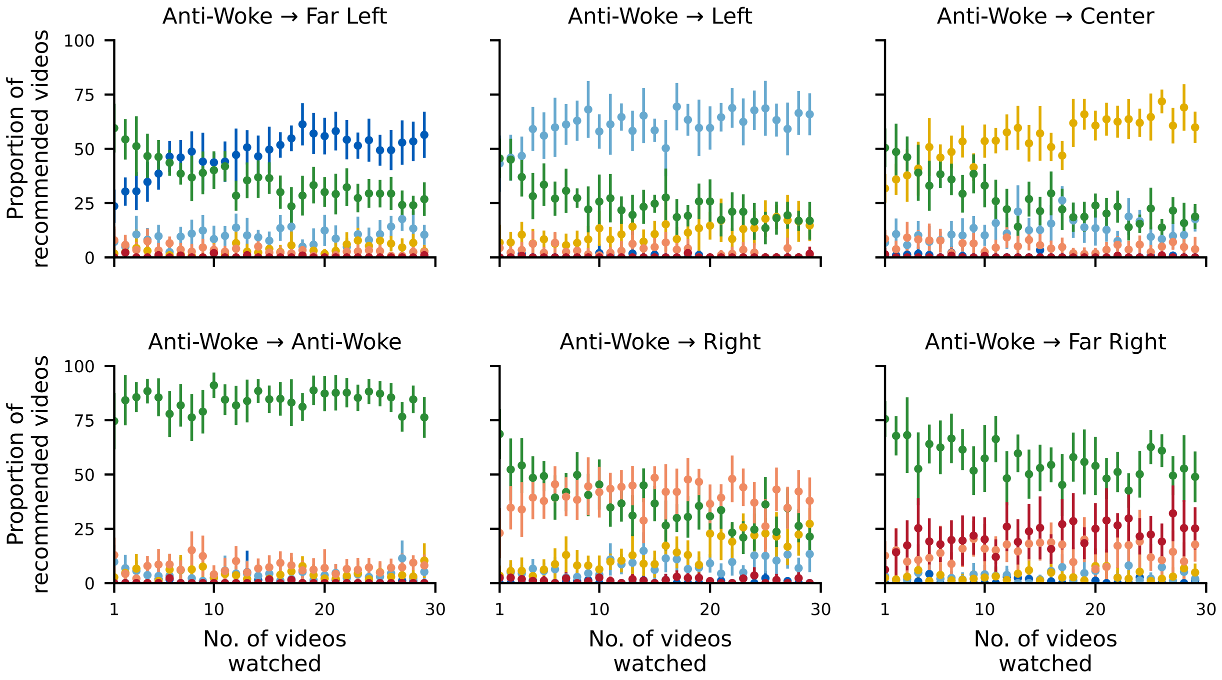

Figure S4: **Transitioning from a pure Anti-Woke persona to different political personas.** Means and 95% confidence intervals of the proportion of different political classes for videos recommended to a user as they watch videos of the target political class. The title of each plot designates the original class of the user, with the arrow pointing to the target class.

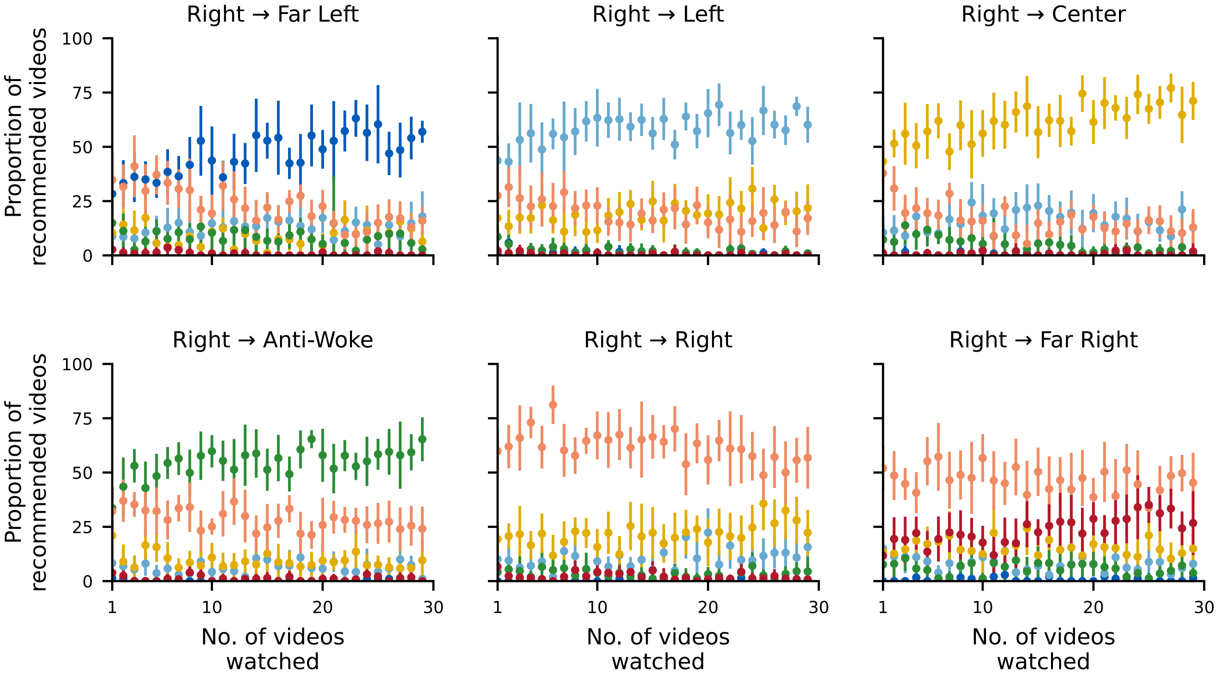

Figure S5: **Transitioning from a pure Right persona to different political personas.** Means and 95% confidence intervals of the proportion of different political classes for videos recommended to a user as they watch videos of the target political class. The title of each plot designates the original class of the user, with the arrow pointing to the target class.

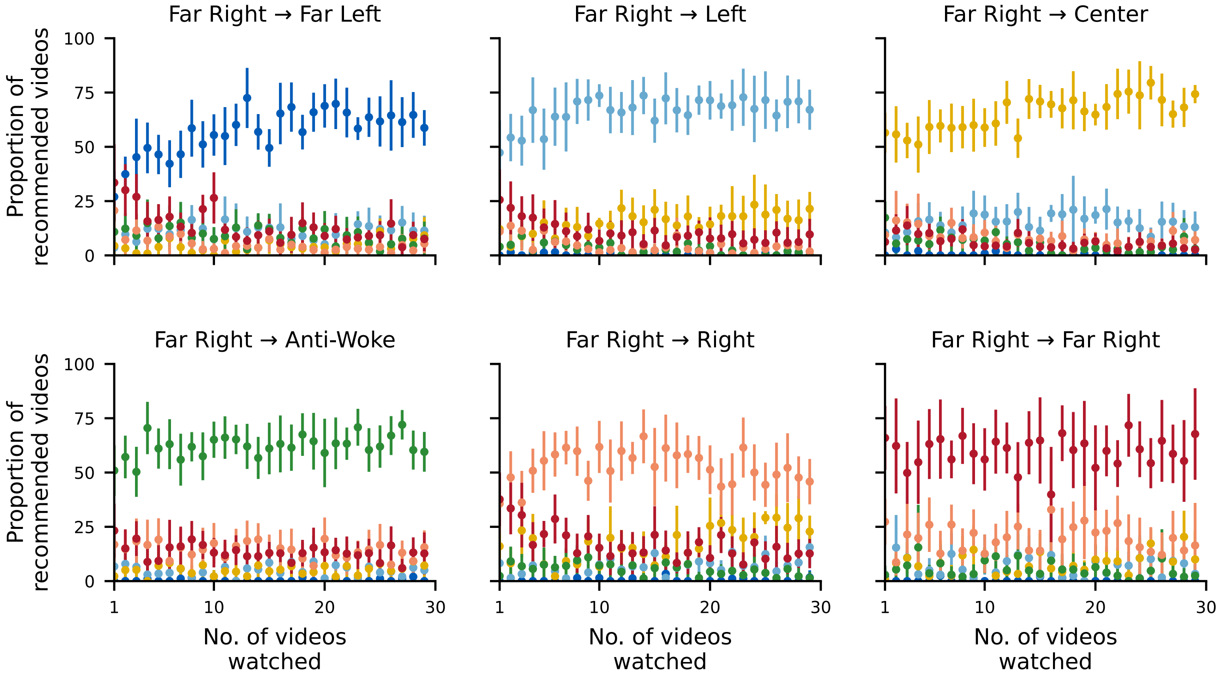

Figure S6: **Transitioning from a pure Far Right persona to different political personas.** Means and 95% confidence intervals of the proportion of different political classes for videos recommended to a user as they watch videos of the target political class. The title of each plot designates the original class of the user, with the arrow pointing to the target class.

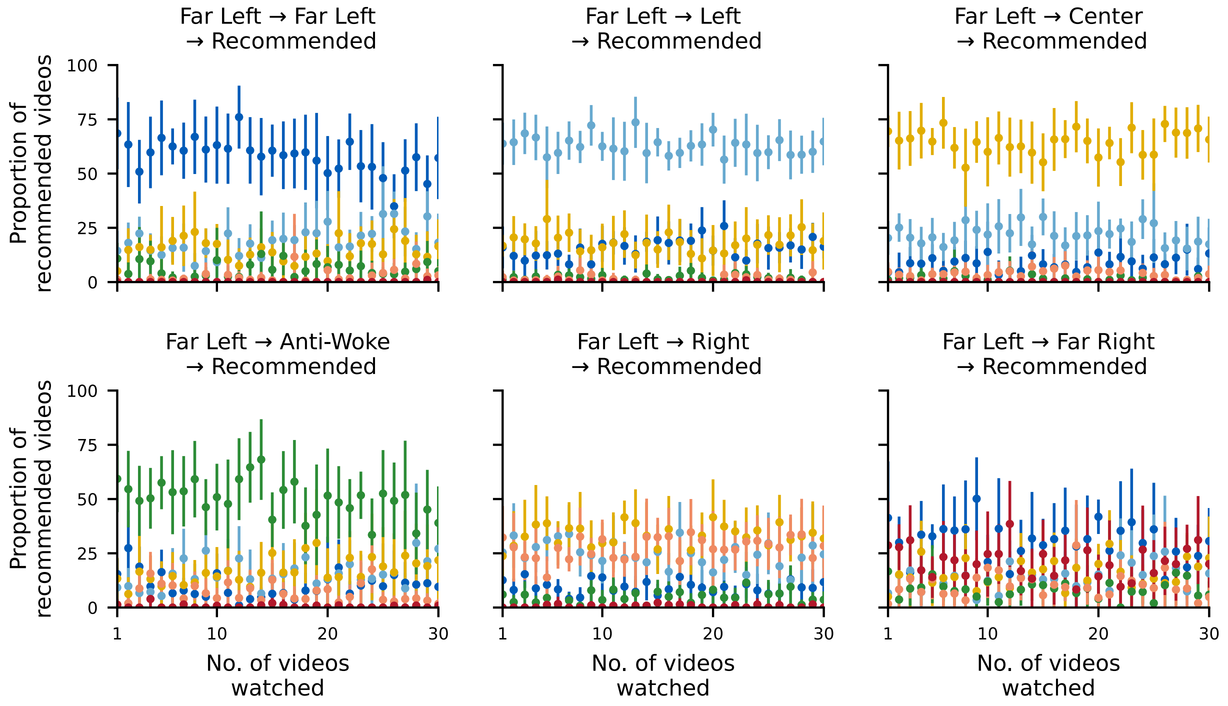

Figure S7: **Recommendation pathways after watching two sequences of 30 videos.** Means and 95% confidence intervals of the proportion of different political classes for videos recommended to a user as they watch recommended videos after watching two sequences of 30 videos, each of which fell under a particular political class. The first class designates the classification of the first sequence of the thirty videos, with the arrow pointing to the the classification of the second sequence of thirty videos.

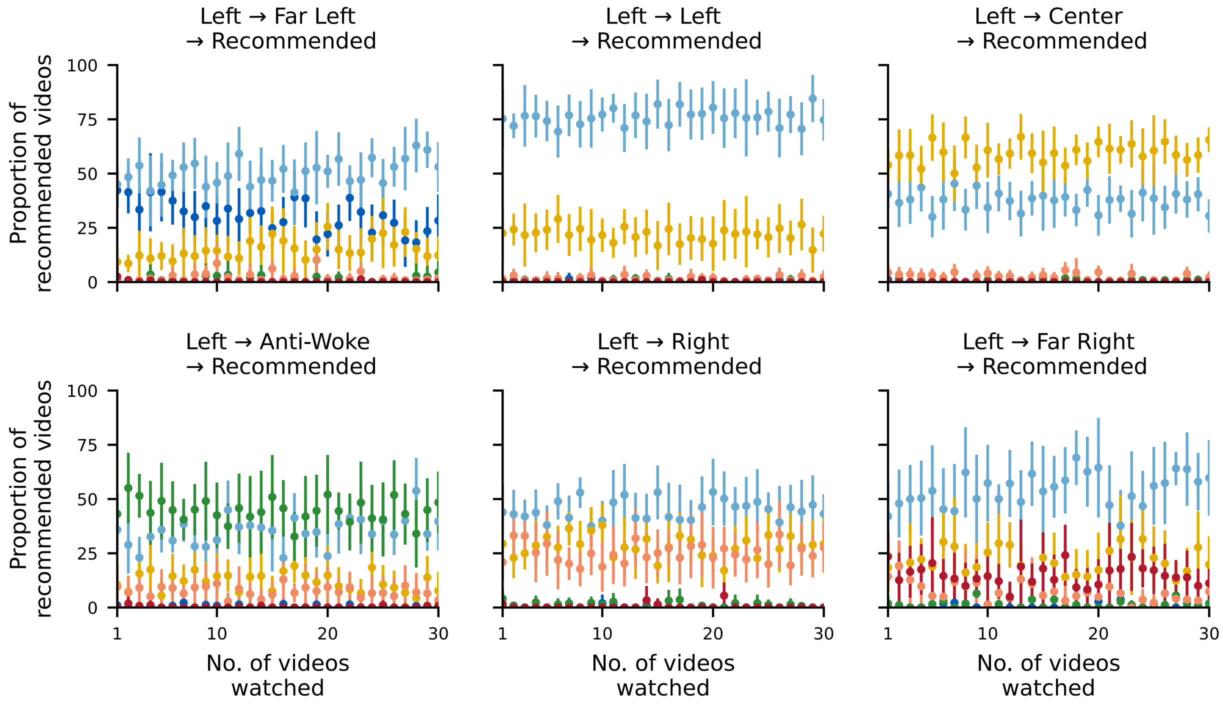

Figure S8: **Recommendation pathways after watching two sequences of 30 videos.** Means and 95% confidence intervals of the proportion of different political classes for videos recommended to a user as they watch recommended videos after watching two sequences of 30 videos, each of which fell under a particular political class. The first class designates the classification of the first sequence of the thirty videos, with the arrow pointing to the the classification of the second sequence of thirty videos.

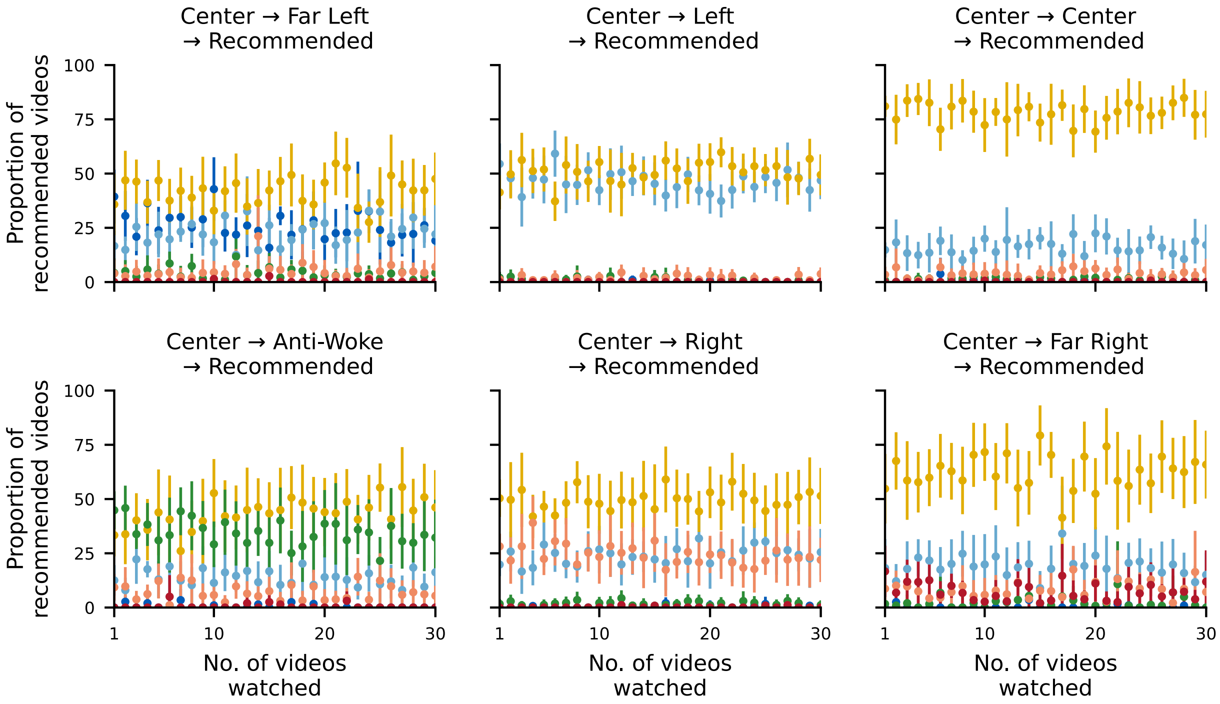

Figure S9: **Recommendation pathways after watching two sequences of 30 videos.** Means and 95% confidence intervals of the proportion of different political classes for videos recommended to a user as they watch recommended videos after watching two sequences of 30 videos, each of which fell under a particular political class. The first class designates the classification of the first sequence of the thirty videos, with the arrow pointing to the the classification of the second sequence of thirty videos.

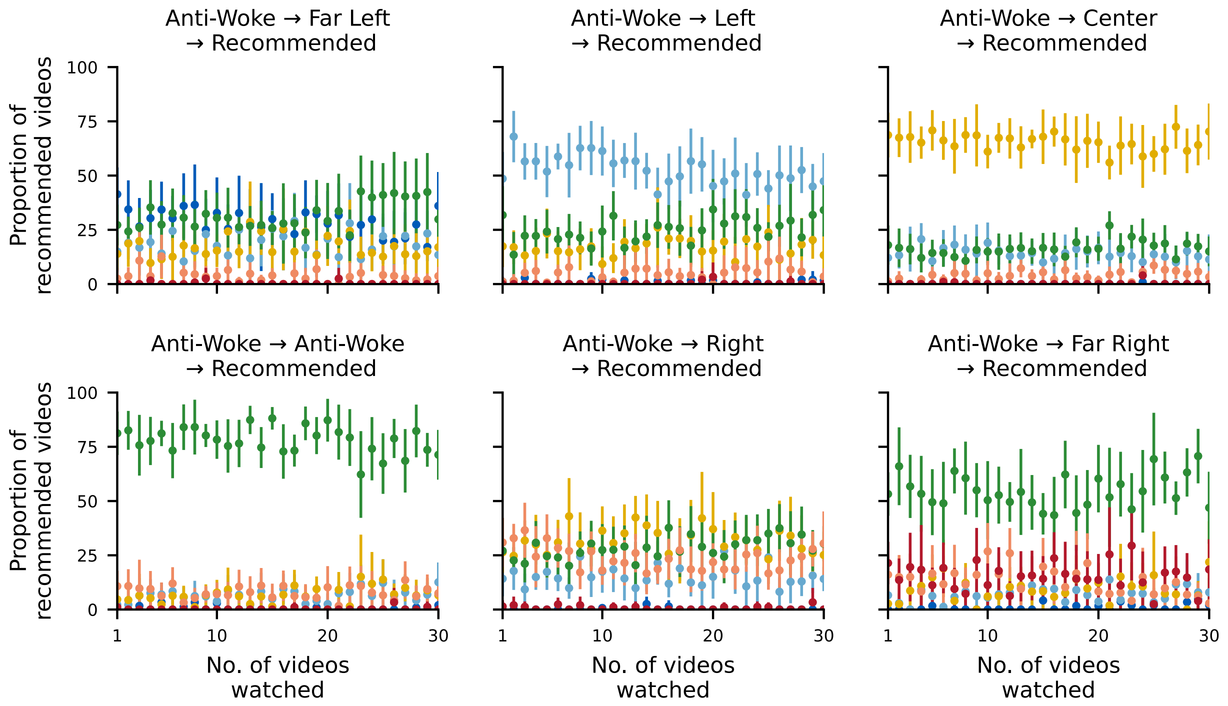

Figure S10: **Recommendation pathways after watching two sequences of 30 videos.** Means and 95% confidence intervals of the proportion of different political classes for videos recommended to a user as they watch recommended videos after watching two sequences of 30 videos, each of which fell under a particular political class. The first class designates the classification of the first sequence of the thirty videos, with the arrow pointing to the the classification of the second sequence of thirty videos.

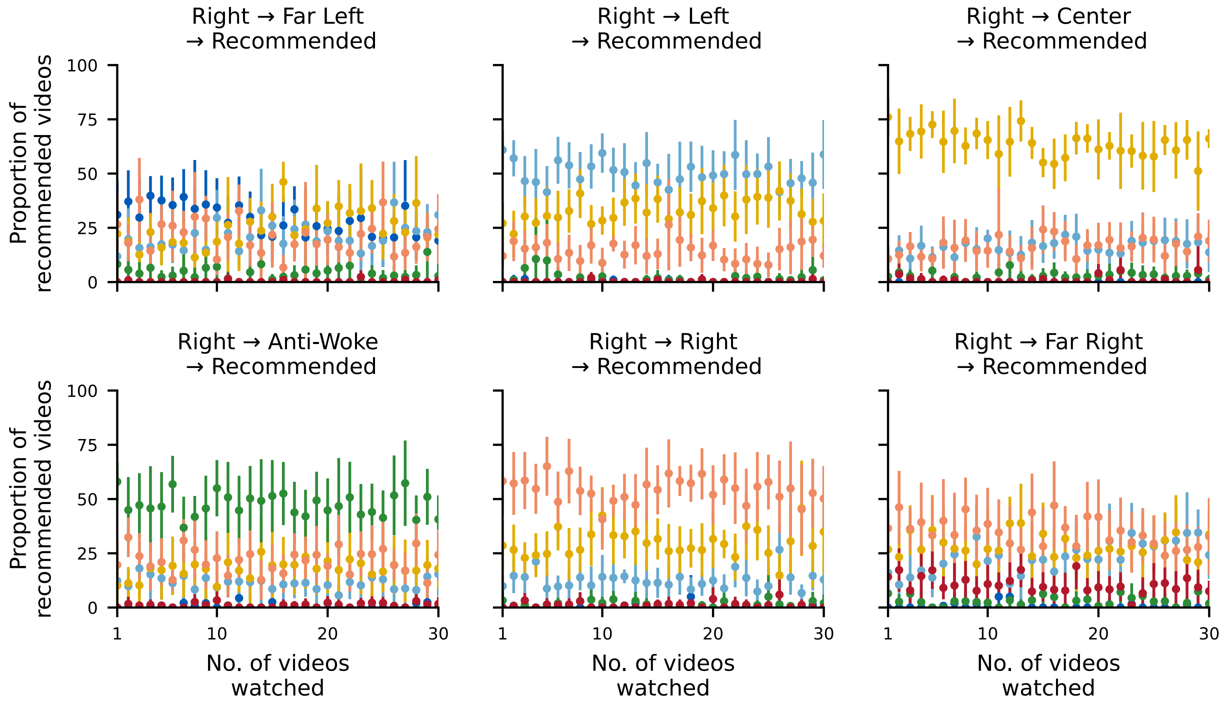

Figure S11: **Recommendation pathways after watching two sequences of 30 videos.** Means and 95% confidence intervals of the proportion of different political classes for videos recommended to a user as they watch recommended videos after watching two sequences of 30 videos, each of which fell under a particular political class. The first class designates the classification of the first sequence of the thirty videos, with the arrow pointing to the the classification of the second sequence of thirty videos.

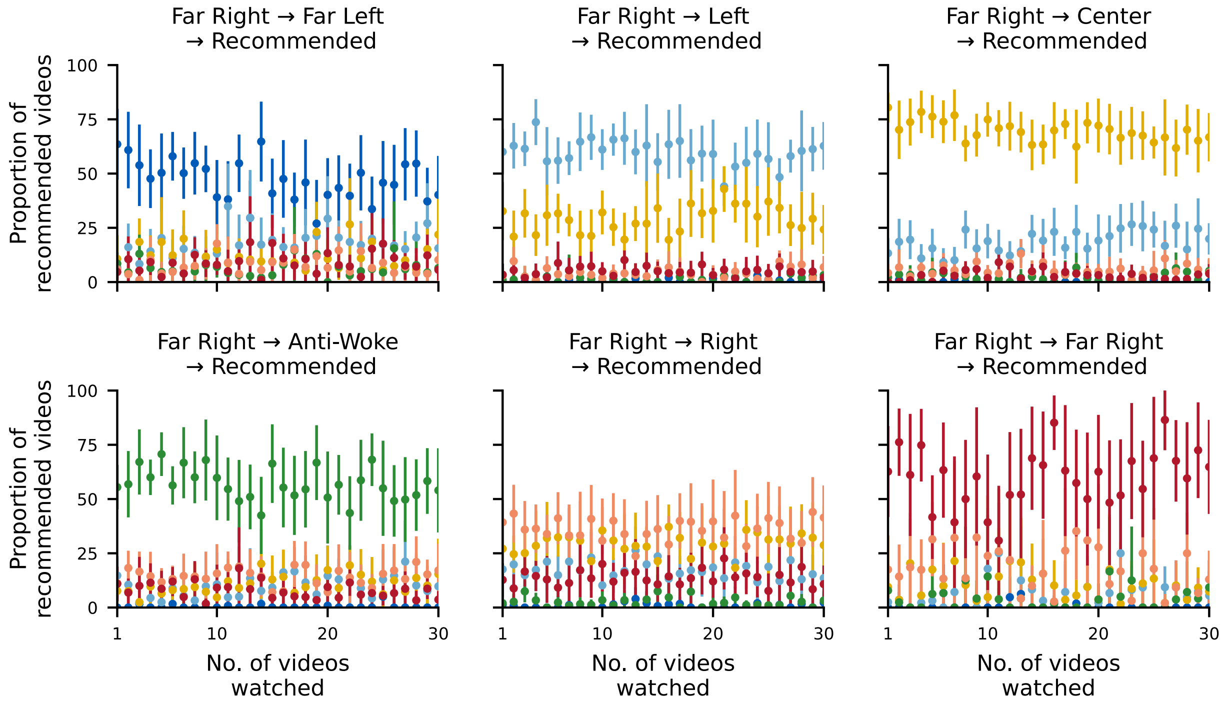

Figure S12: **Recommendation pathways after watching two sequences of 30 videos.** Means and 95% confidence intervals of the proportion of different political classes for videos recommended to a user as they watch recommended videos after watching two sequences of 30 videos, each of which fell under a particular political class. The first class designates the classification of the first sequence of the thirty videos, with the arrow pointing to the the classification of the second sequence of thirty videos.

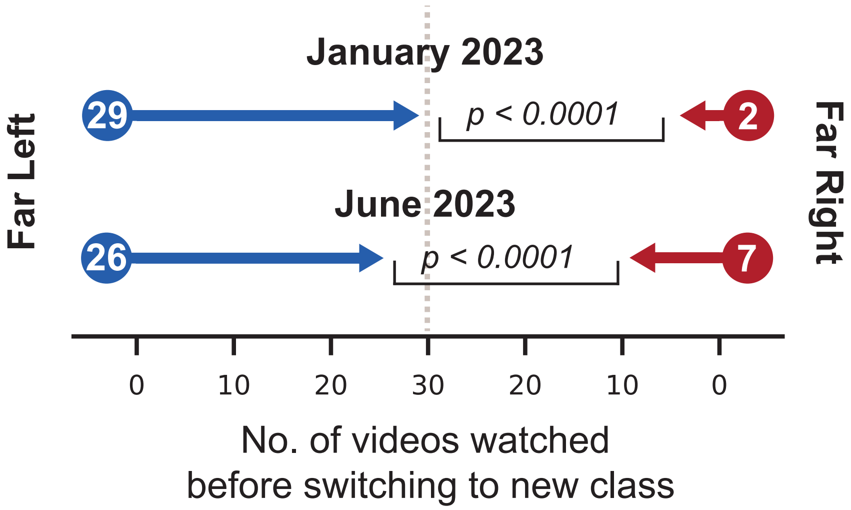

Figure S13: **Replicating the second stage of our experiment.** The average number of videos watched until the user reaches a point at which the proportion of recommended videos with the new class exceeds that of the original class, both in the original experiment (which took place in January 2023) as well as the replicated experiment (which took place in June 2023).
