# Supplementary material for: YouTube’s recommendation algorithm is left-leaning in the United States
Source: PNAS Nexus. 2023 Aug 14;2(8):pgad264. doi: 10.1093/pnasnexus/pgad264 (PMC10433241; doi:10.1093/pnasnexus/pgad264)
Supplement: pgad264_Supplementary_Data [file PNASNEXUS-PNASNEXUS-2023-00588R-s02.pdf › PNASNEXUS-PNASNEXUS-2023-00588R-s02.pdf]

Dear Associate Editors and Reviewers,

We would like to thank you for your feedback. Below, we list all of the changes made in the paper.

As suggested by the Associate Editor, we've significantly narrowed down the scope of the paper, stating explicitly that it is US-centric; the new title is: "Youtube's recommendation algorithm is left-leaning in the United States". The main text has also been modified accordingly.

**Reviewer #2 (Remarks to the Author):**

First, their response to whether the asymmetry in recommendation results stems from the algorithm's preferences or the difference in the number of videos in the two categories needs to be revised to support the question. The authors utilized the Bhattacharyya coefficient to measure the distance between the baseline video's distribution and recommended one. However, the reviewer's question is more related to the numerical asymmetry of the political video's regarding the video's political characteristics. To make a result that 'YouTube algorithm prefers left-leaning videos, and the experimental condition should be set with uniformly distributed political leanings. In that case, if there's a clear preference for Far left or Left-leaning videos, one might be able to conclude that 'the algorithm' itself is biased. However, the current version seems inadequate to conclude the algorithm's bias. Instead, the results seem to demonstrate the asymmetric recommendations in the political videos in the US environment.

**Response:**

With regards to the reviewer's comment on "the numerical asymmetry of the political video's regarding the video's political characteristics", we would like to clarify that we do not compute the Bhattacharyya coefficient of recommended videos against that of **baseline** recommendations, but rather against the distribution of videos in the **pre-labelled dataset**, which included over 11.5 million videos. This analysis allowed us to control for the numerical asymmetry with regards to the number of videos available in each political class in the pre-labelled dataset. Thus, if the left-leaning signal seen in our analysis were to stem from the distribution of videos from the pre-labelled dataset, the "closeness" of the videos recommended when watching Left videos would be closer to that of the pre-labelled dataset than their Right leaning counterparts. We apologize for not making this clear in the original manuscript, and we have now rectified this issue by writing the following:

> *"The Bhattacharyya coefficient quantifies the ``closeness" of two random statistical samples, which in turn allows us to quantify the distance between the distribution of videos recommended for each user against that of the pre-labelled dataset which includes over 11.5 million videos. This analysis allows us to control for the numerical asymmetry with regards to the number of videos available in each political class in the pre-labelled dataset. Thus, if the left-leaning signal seen in this analysis were to stem from the distribution of videos from the pre-labelled dataset, the ``closeness" of the videos recommended when watching Left videos would be closer to that of the pre-labelled dataset than their Right leaning counterparts. However, our results indicate that this is not the case, as users watching Far Left and Left videos are recommended a disproportionately larger proportion of videos falling under the same category when compared to those on the Right and Far Right. In other words, even when accounting for the fact that the Left political class contains more videos than the Right, we still find a statistically significant difference in the proportion of videos recommended."*

In regards to the scope of the title, we agree that our original claim may have been too broad. In light of your comment, we have now toned down our argument by stating that our findings hold in the US political context specifically throughout our paper and in the title of the manuscript.

Second, I appreciate the authors clarifying the IP issue in their experiment. However, diverse external elements can still affect the results, such as the type of mobile phones and their OS types, the regions related to IPs, etc. Controlling the effects and checking their influences would improve the validity of their results, arguing the YouTube algorithm's left-leaning bias.

**Response:**

We agree that the experiment was only done on the Android operating system, and that a reader may wonder whether the same would hold on iPhones. Having said that, we did not find any previous literature suggesting that recommendations made to users by the Youtube recommendation algorithm can be affected by the mobile phone type or the operating system these phones utilize. Nevertheless, we have added the following sentence to the future work section in the Discussion:

> *"Furthermore, while we run our experiment on the Android operating system and on a particular mobile device, namely the Xiaomi Redmi Go, one could explore the degree to which the operating system or type of device on which one consumes content on YouTube affects the types of recommendations they receive."*

On the other hand, with regards to regions related to IPs, we agree that this can affect the recommendation algorithm, which is why we used a VPN to run our experiment on IPs in the US region. Re-running this experiment with the users utilizing IPs of other countries would indeed be an interesting future line of research that addresses the political contexts of other countries. However, since our work is primarily in the US context, we address this comment by toning down our claim of YouTube's left-leaning bias, which in its original form, makes this claim generally. Instead, we make the claim that our results demonstrate the asymmetric recommendations in the political videos in the US environment specifically as recommended by the reviewer.

Other than these, I think the authors should elaborate more on their figure descriptions. In particular, it is difficult to grasp the experiment process in Fig. 1 with the current description. Also, will the sum over the same number of watched videos be 100 percent? Figure 3 needs more explanations on the caption to deliver the meaning of the results as well.

**Response:**

We agree with the reviewer in that the figure descriptions can be elaborated on further to improve clarity. As such, we have added additional detail to the figure descriptions of Figures 1 and 3 in the manuscript. In particular, for Figure 1, which describes the experimental process, we've have amended the figure caption to include the following:

*“**Fig. 1. Experiment setup.** An illustration of the different stages undertaken during our experiment. In the account creation stage, users are designated one of six political classes which denote the class of videos which they will watch in Stage 1. In Stage 1, each user watches 30 videos of a given political class. After each video is watched, the videos recommended to them are collected. After Stage 1, each user is designated a new political class, which will denote the class of videos which they will watch in Stage 2. Similarly, in Stage 2, watch 30 videos of a given political class, and the videos recommended to them will be collected. Finally, in Stage 3, each user will watch the top recommended video to them and subsequently, the set of recommended videos after each video is watched is collected.”*

Similarly, for Figure 3, the caption was changed to the following:

*“**Fig. 3. Distribution of recommendations made after watching videos of a particular political class**. (a) Proportion of recommended videos falling under each political class after each video is watched, with error bars representing 95% confidence intervals. The proportions of videos falling under each political class after each video watched sum up to 100%. (b) Category distribution of recommended videos after each video is watched,where the proportion of videos falling under each category sum up to 100%. The title of each plot indicates the political classification of the videos being watched.”*
